# Phenotypic and genetic associations of quantitative magnetic susceptibility in UK Biobank brain imaging

**DOI:** 10.1038/s41593-022-01074-w

**Published:** 2022-05-23

**Authors:** Chaoyue Wang, Aurea B. Martins-Bach, Fidel Alfaro-Almagro, Gwenaëlle Douaud, Johannes C. Klein, Alberto Llera, Cristiana Fiscone, Richard Bowtell, Lloyd T. Elliott, Stephen M. Smith, Benjamin C. Tendler, Karla L. Miller

**Affiliations:** 1 Wellcome Centre for Integrative Neuroimaging, FMRIB, Nuffield Department of Clinical Neurosciences, University of Oxford, Oxford, UK; 2 Oxford Parkinson’s Disease Centre, University of Oxford, Oxford, UK; 3 Donders Institute for Brain, Cognition and Behaviour, Centre for Cognitive Neuroimaging, Nijmegen, the Netherlands; 4 Sir Peter Mansfield Imaging Centre, School of Physics and Astronomy, University of Nottingham, Nottingham, UK; 5 Department of Biomedical and Neuromotor Sciences, University of Bologna, Bologna, Italy; 6 Department of Statistics and Actuarial Science, Simon Fraser University, Vancouver, British Columbia Canada

**Keywords:** Neuroscience, Biomarkers, Genetics, Research data

## Abstract

A key aim in epidemiological neuroscience is identification of markers to assess brain health and monitor therapeutic interventions. Quantitative susceptibility mapping (QSM) is an emerging magnetic resonance imaging technique that measures tissue magnetic susceptibility and has been shown to detect pathological changes in tissue iron, myelin and calcification. We present an open resource of QSM-based imaging measures of multiple brain structures in 35,273 individuals from the UK Biobank prospective epidemiological study. We identify statistically significant associations of 251 phenotypes with magnetic susceptibility that include body iron, disease, diet and alcohol consumption. Genome-wide associations relate magnetic susceptibility to 76 replicating clusters of genetic variants with biological functions involving iron, calcium, myelin and extracellular matrix. These patterns of associations include relationships that are unique to QSM, in particular being complementary to T2* signal decay time measures. These new imaging phenotypes are being integrated into the core UK Biobank measures provided to researchers worldwide, creating the potential to discover new, non-invasive markers of brain health.

## Abstract (web summary)

Wang et al. present a new open resource from the UK Biobank using quantitative susceptibility mapping, a neuroimaging marker sensitive to iron and myelin. They demonstrate a broad range of phenotypic and genetic associations in 35,885 participants.

## Main

Magnetic resonance imaging (MRI) of the brain visualizes anatomical structures on the scale of millimeters but is sensitive to microscopic tissue features. This sensitivity confers the potential to detect the earliest stages of disease for therapeutic development and disease monitoring. In many cases, it is difficult or impossible to design small cohorts that reliably capture asymptomatic early disease; a powerful alternative is to prospectively image healthy individuals at a large scale and track subsequent disease. The UK Biobank study is collecting brain images in 100,000 participants who are largely healthy when scanned^1^. Participants have been deeply phenotyped and genotyped and consented to long-term access to their health records. UK Biobank has identified relationships between brain imaging markers and phenotypes, including obesity^2^, vascular disease^3^ and aging^4^. It has also enabled major new insights into the genetic correlates of imaging phenotypes^5,6^, identifying genes with known links to psychiatric illness^7^, vascular disease^8^ and neurodegeneration^9^.

UK Biobank is not yet fully exploiting the available brain imaging data, particularly the susceptibility-weighted MRI (swMRI) scan collected using a gradient echo protocol. swMRI signals are influenced by iron, myelin and calcium content due to the shifted magnetic susceptibility (*χ*) of these constituents relative to tissue water^10^. The signal magnitude from swMRI has been analyzed to provide estimates of signal decay time (T2*)^1,11^, but the signal phase has not previously been analyzed. Recently developed algorithms for quantitative susceptibility mapping (QSM) transform swMRI phase data into quantitative estimates of *χ* (ref. ^12^). While derived from the same scan as T2*, QSM conveys distinct information. QSM estimates the mean *χ* within a voxel, reflecting bulk content of susceptibility-shifted sources like iron, whereas T2* reflects the variance of *χ*-induced magnetic field fluctuations, relating to compartmentalization of these same sources^13^. One key consequence of this is that paramagnetic substances (for example, iron) and diamagnetic substances (for example, myelin) have the opposite effect on *χ* in QSM but the same effect on T2*. QSM has been demonstrated to detect disease-relevant changes, such as iron accumulation in neurodegenerative disorders^14,15^, and to provide an index of microstructural changes to tissue in normal aging^16^. UK Biobank brain imaging thus creates unique opportunities to investigate QSM in previously unexplored territories, including as an early disease marker.

We developed a QSM pipeline for UK Biobank that was run on the current release of 35,273 participants (with repeat imaging in 1,368 participants) and produced imaging-derived phenotypes (IDPs) of *χ* in various brain structures. These data constitute an enhancement to the UK Biobank neuroimaging resource through the provision of QSM-derived *χ* estimates. To demonstrate the scientific value of this resource, we present phenotypic and genetic associations, focusing on examples that demonstrate the new information contributed by QSM. Our QSM processing has been incorporated into the core UK Biobank brain imaging processing pipeline^11^. QSM IDPs have been provided to UK Biobank (spatial *χ* maps will be provided subsequently) to be made available to researchers worldwide. We encourage interpretation and validation of specific associations of interest based on previous literature or follow-up studies. Our results demonstrate the richness of information in QSM data and the added value of QSM to the UK Biobank resource.

## Results

### Overview of QSM processing and image analyses

**Fig. 1:**
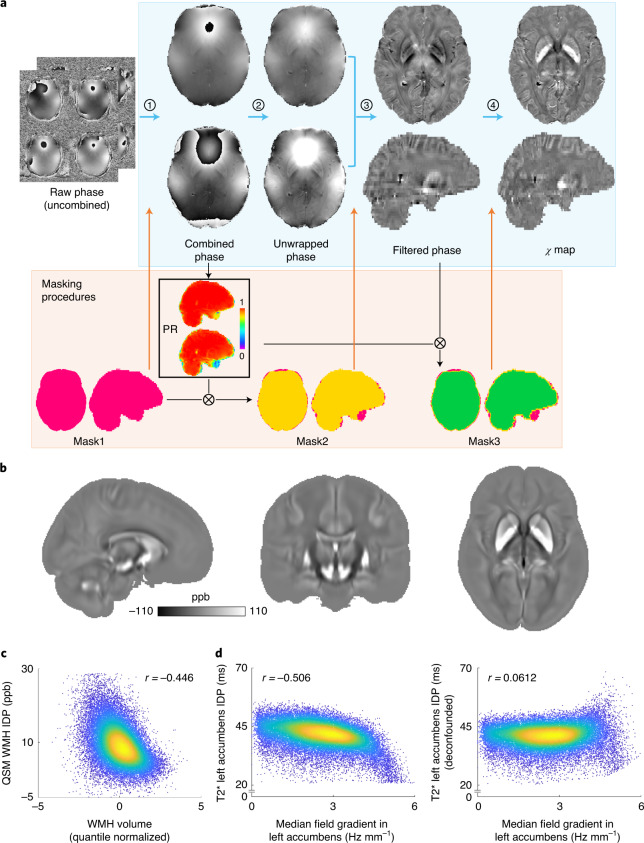
QSM processing and IDP analyses. **a**, QSM processing pipeline for UK Biobank swMRI data. Blue arrows indicate the main processing steps. Step 1, channel combination using MCPC-3D-S. Step 2, phase unwrapping using a Laplacian-based algorithm. Step 3, background field removal using V-SHARP. Step 4, dipole inversion using iLSQR. Black arrows indicate the brain mask evolution, and orange arrows indicate the brain mask applied at each step. Briefly, the brain mask provided by UK Biobank (Mask1, pink) was first used for the channel combination step. To exclude unreliable voxels in the vicinity of sinus cavities, the mask was subsequently refined using a ‘phase reliability’ map (PR, black box; Mask2, yellow). After background field removal, the output mask from V-SHARP was further refined using the phase reliability map, with the resulting mask (Mask3, green) used for dipole inversion. Full details about the pipeline are provided in Methods. **b**, QSM atlas generated by averaging *χ* maps (non-linearly registered to MNI space) from 35,273 individuals; ppb, parts per billion. **c**, Association between QSM WMH IDP and WMH volume IDP (*r* = –0.446). **d**, Example association between T2* left accumbens IDP and median field gradient measured in the left accumbens before (*r* = –0.506) and after (*r* = 0.0612) deconfounding based on a physical model (details in Supplementary Information, section 2); *n* = 35,273 individuals were used to compute Pearson correlation *r* values shown in **c** and **d**.

We conducted an extensive evaluation of existing algorithms for each QSM processing step to establish an automated QSM pipeline (Supplementary Information, section 1.1). The final pipeline is illustrated in Fig. 1a and detailed in Methods. Briefly, individual channel phase images for each echo are combined using MCPC-3D-S^17^ and unwrapped using a Laplacian-based algorithm^18^, and the two echoes are combined with weighted averaging.^19^. Brain-edge voxels with extremely large phase variance (primarily near sinuses) are detected and excluded. Background fields are removed using the variable kernel sophisticated harmonic artifact reduction for phase data (V-SHARP) algorithm^20^. Finally, *χ* maps are calculated using iLSQR^21^ and referenced to cerebrospinal fluid (CSF) in the lateral ventricles.

The pipeline was run on the 35,273 individuals with usable swMRI data, and each individual’s *χ* map was transformed to Montreal Neurological Institute (MNI) standard space. A QSM population average template (Fig. 1b) was produced by averaging all *χ* maps, and an ‘aging’ template (Supplementary Fig. 26b,d) was calculated as the difference between the average *χ* maps for groups of youngest (<52 years old) and oldest (>75 years old) individuals (each group had around 2,000 individuals) to visualize age-associated atrophy.

### IDP preparation

Median T2* IDPs in 14 major subcortical gray matter regions (accumbens, amygdala, caudate, hippocampus, pallidum, putamen and thalamus, both left and right) are already available in the current UK Biobank data release. We produced equivalent QSM-based IDPs, calculated as median *χ* values in these same 14 subcortical regions. We additionally extracted the median *χ* and T2* in the substantia nigra (left and right), bringing the total number of subcortical regions for both *χ* and T2* to 16. The UK Biobank also provides masks of white matter hyperintensities (WMHs) derived from the T2-weighted structurals^11^, which we used to derive the difference in *χ* or T2* between WMH lesions and normal-appearing white matter. We observed strong correlations between WMH IDPs (both QSM and T2*) and WMH volume (Fig. 1c). QSM and T2* WMH IDPs were thus additionally processed by regressing out the WMH volume, resulting in a total of 18 IDPs (16 subcortical and 2 WMH IDPs) for QSM or T2*.

We incorporated a new confound regressor based on a physical model^22^ that accounts for biases in T2* estimates introduced by macroscopic field gradients (Supplementary Information, section 2). As expected, this confound regressor correlated significantly with T2* IDPs (Fig. 1d) but not QSM (Supplementary Fig. 16) and as such was incorporated into phenotypic and genetic associations using T2* but not QSM IDPs.

### Reproducibility analysis

A reproducibility analysis using the two-timepoint data from 1,368 individuals demonstrated that subcortical QSM IDPs generally showed higher cross-scan correlation *r* than corresponding T2* IDPs (Supplementary Fig. 10b), particularly in the putamen, caudate, pallidum and substantia nigra (*r* > 0.8 for QSM and 0.6 < *r* < 0.8 for T2*).

### Associations between IDPs and non-imaging phenotypes

We performed univariate (pairwise) association analyses between 17,485 UK Biobank non-imaging phenotypes and QSM/T2* IDPs, where Pearson correlation *r* and *P* values were calculated. For the remainder of this manuscript, we refer to all non-imaging phenotypes as ‘phenotypes’ to distinguish them from IDPs. These phenotypes have been grouped into 17 categories, including early life factors (for example, birth weight and maternal smoking), lifestyle (for example, diet and alcohol consumption), physical/body measures (for example, body mass index (BMI) and blood assays), cognitive test scores (for example, numeric memory), health outcomes (for example, clinical diagnoses, as the International Classification of Diseases (ICD10) codes) and mental health variables (for example, major depression). A well-established set of confounds (including age and sex; Methods)^11,23^ have been regressed out from the associations with phenotypes.

**Fig. 2:**
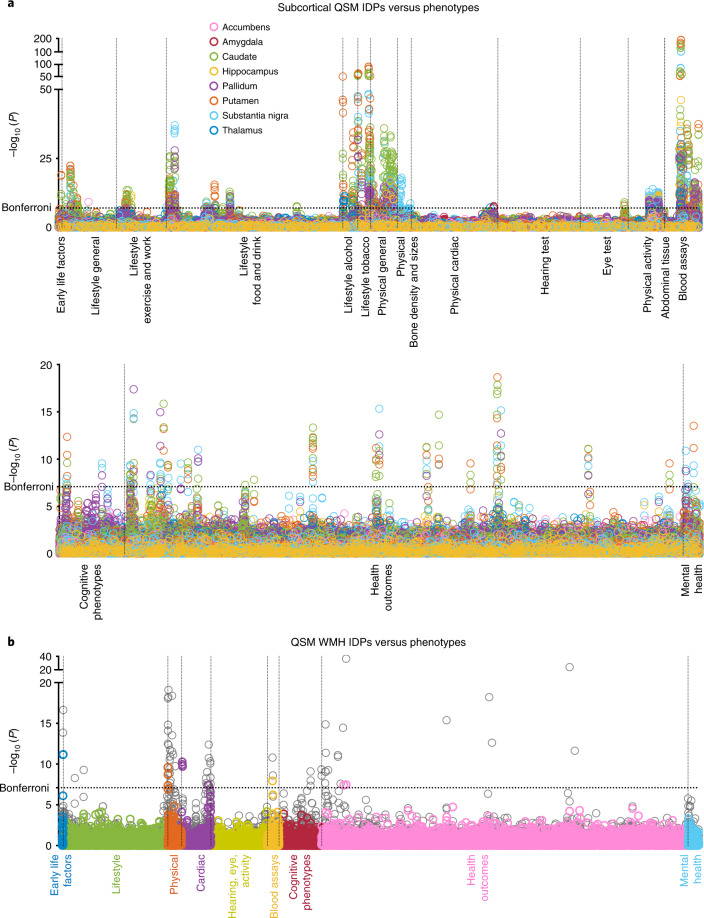
Visualization of univariate (two-sided) cross-participant association tests between 18 QSM IDPs and the 17,485 phenotypes in UK Biobank using *n* = 35,273 participants. The significance threshold was adjusted to account for multiple comparisons, and unadjusted –log_10_ (*P*) values are reported. Each circle represents a single IDP–phenotype association. The dashed horizontal line indicates the –log_10_ (*P*) Bonferroni-corrected threshold of 7.10. All associations above this line are considered significant. Dashed vertical lines are used to distinguish between different phenotype categories. **a**, Manhattan plots showing associations between 16 subcortical QSM IDPs and phenotypes in 17 categories. **b**, Manhattan plot showing associations between the QSM WMH IDPs and all phenotypes (separated into nine major categories). Shown behind (gray) are the associations without regressing out WMH volume.

As these phenotypic associations had widely varying degrees of freedom (due to varying amounts of missing data in phenotypes), *P* values were used to identify the strongest associations. As the reported associations are conducted with a broad range of variables with different (and often arbitrary) units, we normalized both parameters in a given association such that the Pearson correlation (*r* values) served as an effect size. Due to the large number of individuals in UK Biobank, statistically significant correlations often correspond to small *r* values. This is a common characteristic in population imaging^24^ and is in line with the associations observed in the original UK Biobank neuroimaging report^1^. The full set of 629,460 (17,485 phenotypes × 36 IDPs) correlations was corrected for multiple comparisons. In this work, all multiple comparison corrections were implemented by adjusting the significance thresholds, with *P* values unchanged. We follow the convention for Manhattan plots and display results using –log_10_ (*P*) (Fig. 2). Bonferroni correction for family-wise error control at *P*_corrected_ < 0.05 was applied, corresponding to a –log_10_ (*P*_uncorrected_) of 7.10. Additionally, a less conservative option for multiple comparison correction is false discovery rate (FDR), which for a 5% FDR resulted in a –log_10_ (*P*_uncorrected_) of >3.13. In this manuscript, we primarily focus on associations passing the Bonferroni-corrected threshold, according to which we identified statistically significant associations of 251 phenotypes with QSM IDPs and 224 phenotypes with T2* IDPs. The total number of significant associations is much larger than this, as this count pools multiple timepoint measurements of the same phenotype and multiple IDPs associating with the same phenotype. The full list of significant phenotypic associations is provided in Supplementary Table 1.

**Fig. 3:**
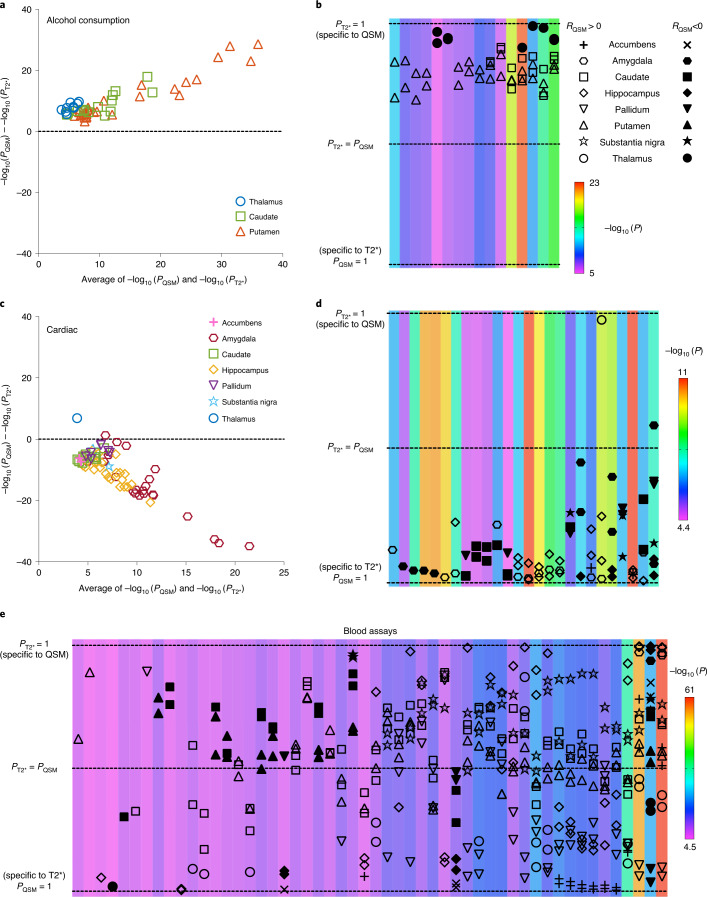
Example comparisons of (two-sided) phenotypic associations with QSM and T2* subcortical IDPs (region/phenotype pair shown if unadjusted *P*_QSM_ or *P*_T2*_ passed the Bonferroni-corrected threshold) using *n* = 35,273 participants. **a**–**e**, Here, we display results for alcohol consumption (**a** and **b**), cardiac (**c** and **d**) and blood assays (**e**) categories. **a**,**c**, Bland–Altman plot showing comparisons of –log_10_ (*P*) values for QSM and T2* associations with alcohol consumption (**a**) and cardiac (**c**) categories. **b**,**d**,**e**, Transformed Bland–Altman plot that aims to emphasize whether a given association is specific to QSM or T2* or is common to both. Each column represents one unique phenotype from the corresponding Bland–Altman plot, ordered from left to right by the number of associated regions. The vertical axis is given by the angle of each point in a Bland–Altman plot with respect to the *y* = 0 line. Hence, datapoints at the top (or bottom) of the plot represent an association that is highly specific to QSM (or T2*), and datapoints in the middle are phenotypes that associate with both QSM and T2* in a given brain region. The background color of each column represents the averaged –log_10_ (*P*) value for significant associations with that phenotype. Unlike the Bland–Altman plot, this visualization emphasizes the modality specificity over the strength of correlation. For example, it is more apparent in **b** than in **a** that thalamus–alcohol associations are highly specific to QSM. Here, the three categories reveal more QSM-specific (**a** and **c**), T2*-specific (**b** and **d**) and mixed (**e**) association patterns.

We compared the strength of QSM and T2* associations for each phenotype category (full results are presented in Supplementary Information, section 3). Associations in some phenotype categories (for example, alcohol consumption) are more specific to QSM IDPs (Fig. 3a,b), whereas other categories (for example, cardiac) are more specific to T2* IDPs (Fig. 3c,d). However, the majority of phenotype categories show a mixed pattern of associations, including both common and distinct associations (for example, blood assays; Fig. 3e). This overall picture agrees with the expectation that QSM and T2* measures do not trivially recapitulate the same tissue properties but together provide rich information from a single scan.

**Fig. 4:**
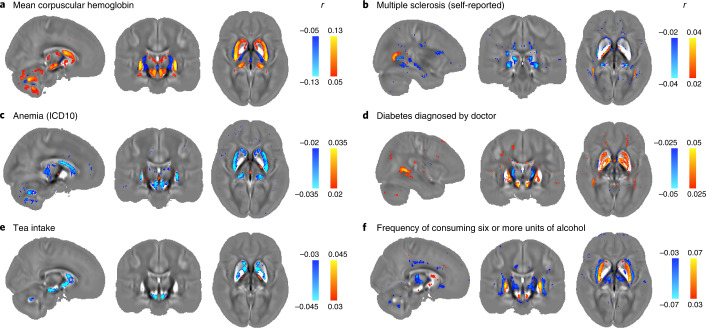
Voxel-wise association maps of six example phenotypes with *χ* maps (aligned in MNI space) from 35,273 participants. The Pearson correlation *r* is shown as color overlay (red–yellow for positive *r* and blue for negative *r*) on the population average *χ* map. **a**, Mean corpuscular hemoglobin identifies all subcortical regions captured by our IDPs as well as the red nucleus and cerebellar regions. Particularly, the putamen, caudate, substantia nigra and red nucleus exhibit homogeneous correlations across the entire region. **b**, Multiple sclerosis (self-reported) identifies subregions of the thalamus (including the pulvinar nucleus and lateral geniculate nucleus) as well as focal white matter regions, such as the optic radiation. **c**, Anemia (ICD10) identifies the putamen, caudate, red nucleus and cerebellar regions as well as subregions of the substantia nigra and thalamus. **d**, Diabetes diagnosed by doctor identifies subregions of the caudate, putamen, pallidum and substantia nigra in addition to white matter regions, including the splenium of the corpus callosum and optic radiations. **e**, Tea intake identifies subregions of the caudate, pallidum and substantia nigra. **f**, Frequency of consuming six or more units of alcohol identifies the putamen and subregions of the thalamus, caudate and substantia nigra.

Having identified phenotypes that associate with QSM IDPs, we conducted voxel-wise regressions with these same phenotypes into *χ* maps to investigate the spatial regions driving these associations. Fig. 4 shows voxel-wise associations of *χ* with six representative phenotypes. Voxel-wise association maps with representative associations in each phenotype category are provided in Supplementary Information, section 4. In general, voxel-wise association maps are highly symmetric, including extended homogeneous regions, more focal associations with subregions and associations with brain areas not included in the regions of interest (ROIs) used to generate QSM IDPs.

We now describe associations in specific phenotype categories in more detail, focusing on associations that recapitulate previous studies or are more specific to QSM IDPs. An overview of these categories is given in Table 1.

**Table 1:** Summary of association results in four phenotype categories

Phenotype category	Number of phenotypes	Brain regions (IDPs)	Phenotype/IDP associations used to derive voxel-wise maps in Fig. 4
Blood assays	33	All 16 subcortical regions, WMH IDPs	Mean corpuscular hemoglobin versus *χ* in right putamen (*r* = 0.16, –log_10_ (*P*) = 190.37)
Health outcomes	44	MS: thalamus, WMH IDPs; anemia: putamen, caudate, substantia nigra; diabetes: pallidum, substantia nigra, caudate, putamen	MS (self-reported) versus *χ* in WMH (*r* = −0.068, –log_10_ (*P*) = 36.67); anemia (ICD10) versus *χ* in left putamen (*r* = −0.048, –log_10_ (*P*) = 18.65); diabetes diagnosed by doctor versus *χ* in right pallidum (*r* = 0.047, –log_10_ (*P*) = 17.38)
Food and drink	22	Substantia nigra, pallidum, caudate, putamen, hippocampus	Tea intake versus *χ* in right substantia nigra (*r* = −0.069, –log_10_ (*P*) = 37.01)
Alcohol consumption	10	Putamen, caudate, thalamus	Frequency of consuming six or more units of alcohol versus *χ* in thalamus (*r* = −0.043, –log_10_ (*P*) = 9.94)

The Pearson correlation *r* and –log_10_ (*P*) values provided are unadjusted and calculated from two-sided association analyses.

### Phenotypic associations with QSM IDPs in four categories

#### Blood assays

Phenotypic associations with blood assays include hemoglobin, cell counts, cell morphology and blood constituents. The strongest of these are hemoglobin-related phenotypes, which show positive correlations with QSM IDPs (Fig. 2a and Supplementary Table 1). The voxel-wise map of association with mean corpuscular hemoglobin (Fig. 4a; the strongest phenotypic correlation) reveals spatially contiguous positive associations in all subcortical regions captured by our IDPs as well as the red nucleus and cerebellar regions. Here, the putamen, caudate, substantia nigra and red nucleus exhibit homogeneous correlations across the entire region, while voxels in the pallidum, hippocampus and thalamus localize to specific subregions. Hemoglobin-related blood measures are used clinically as a marker for an individual’s iron level^25^, and the positive sign of associations with hemoglobin measures is consistent with both QSM’s established relationship with iron, and the positive correlation with iron concentration from postmortem studies^26^. Associations with QSM WMH IDPs are distinct to subcortical regions, exhibiting specificity to red blood cells and hematocrit.

#### Health outcomes

QSM IDPs are associated with diagnoses of multiple sclerosis (MS), anemia and diabetes. The strongest associations in the health outcomes category are MS (self-reported) versus QSM WMH IDP (*r* = −0.068) and T2* WMH IDP (*r* = 0.064). Previous literature^27^ reported variations of *χ* in MS white matter lesions (relative to normal-appearing white matter) across disease stages. Notably, our WMH IDPs were calculated in all participants irrespective of diagnosis and thus likely represent a mixture of pathology (including MS) and normal aging. Furthermore, previous literature reported decreased *χ* in the thalamus (particularly pulvinar nucleus) for individuals with MS compared to healthy volunteers^28^. The association between QSM right thalamus IDP and MS (self-reported; *r* = −0.028, –log_10_ (*P*) = 6.85) is significant at the FDR-corrected threshold (–log_10_ (*P*) = 3.13) but is just below the Bonferroni-corrected threshold (–log_10_ (*P*) = 7.1). The voxel-wise association map with MS (self-reported; Fig. 4b) reveals spatially contiguous negative associations in subregions of the thalamus (including the pulvinar nucleus and lateral geniculate nucleus) as well as focal white matter regions, such as the optic radiation. This suggests that the subthreshold association at IDP level may be due to the use of ROIs covering the entire thalamus that dilute significance of results that are specific to subregions. Interestingly, previous literature has reported structural damage of the thalamic lateral geniculate nucleus in individuals with MS^29^, reflecting potential damage of the visual pathway in MS. Associations with self-reported and diagnosed anemia are consistent with a reduction in tissue iron^26^, finding negative correlations with *χ* and positive correlations with T2*. The voxel-wise association map with anemia (ICD10; Fig. 4c) reveals spatially contiguous negative associations in the putamen, caudate, red nucleus and cerebellar regions as well as subregions of substantia nigra and thalamus. QSM associations with diabetes include formal diagnosis, self-report and relevant medication (insulin and metformin). The voxel-wise association map with diabetes diagnosed by doctor (Fig. 4d) reveals spatially contiguous positive associations in the caudate, putamen, pallidum and substantia nigra regions in addition to white matter, including the splenium of the corpus callosum and optic radiations. Body iron overload in diabetes has been frequently reported^30^, with a recent brain imaging study finding increased *χ* in the caudate, putamen and pallidum in type 2 diabetes^31^, in agreement with our results. Finally, QSM WMH IDPs correlated with hypertension and measures of blood pressure; vascular risk factors (including hypertension) have been reported to have an effect on MS pathology^32^, which may result in changes of *χ* in WMH lesions.

#### Food and drink

QSM IDPs are associated with food and drink intake, including tea, coffee, meat and carbohydrate consumption as well as dietary supplements (Supplementary Table 1). The strongest associations relate to tea consumption, which correlates negatively with *χ*. Although no direct links between tea intake and *χ* measures have been described previously, polyphenols in both green and black tea have been reported as brain-permeable natural iron chelators that have demonstrated neuroprotective effects^33,34^. The voxel-wise association map with tea intake (Fig. 4e) reveals spatially contiguous negative associations in subregions of the caudate, pallidum and substantia nigra. This spatial pattern of correlations is in line with a previous rodent study in which black tea extract reduced oxidative stress levels in the substantia nigra and striatum^33^.

#### Alcohol consumption

Alcohol consumption correlated more strongly with QSM IDPs than T2* IDPs in all cases (Fig. 3a,b). The voxel-wise association map with frequency of consuming six or more units of alcohol (Fig. 4f) reveals spatially contiguous positive associations in the putamen and subregions of substantia nigra and caudate but also negative associations in subregions of the thalamus. These results recapitulate a previous study finding higher *χ* in the putamen, caudate and substantia nigra in individuals with alcohol use disorder^35^, which has been linked to abnormal body iron accumulation^36^. *χ* in the thalamus correlated with phenotypes relating to the quantity of alcohol consumption, in some cases (for example, frequency of consuming six or more units of alcohol) having no significant correlation with T2*. The thalamus is involved in the frontocerebellar circuit and Papez circuit, which are particularly affected by alcohol consumption^37,38^. Although no previous studies have linked *χ* in the thalamus with alcohol use disorder, neuroimaging studies have reported reductions in thalamic volume and connectivity in individuals with alcohol use disorder^37,38^.

### Heritability of QSM and T2* IDPs

**Fig. 5:**
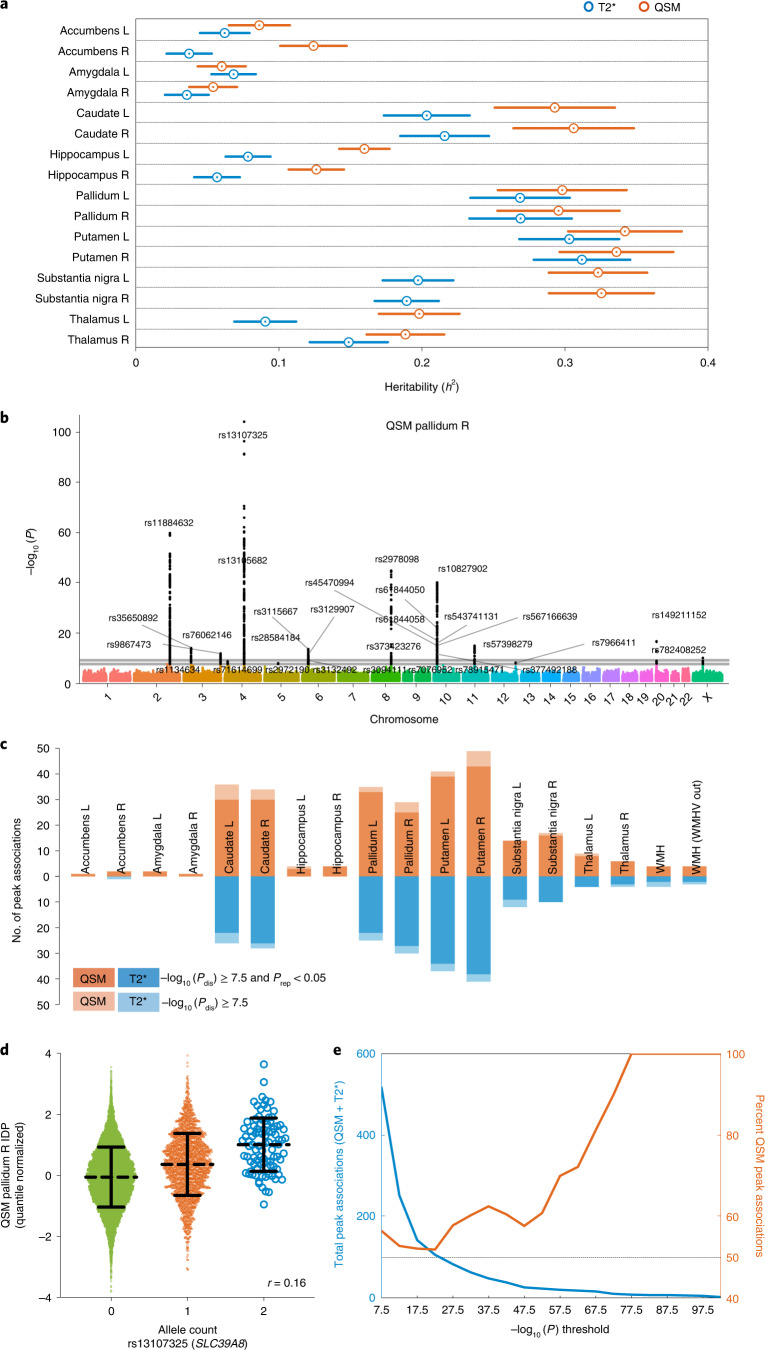
Heritability and genetic associations of QSM and T2* IDPs. **a**, Heritability estimates (*h*^2^) using *n* = 29,579 unrelated participants for subcortical QSM and T2* IDPs grouped according to regions. Circles indicate heritability estimates, and error bars indicate standard error; R, right; L, left. **b**, Example Manhattan plot of the GWAS for the QSM right pallidum IDP (two-sided, unadjusted –log_10_ (*P*) values for the discovery cohort *n* = 19,720). The lower gray horizontal line indicates the –log_10_ (*P*) threshold of 7.5, and the upper line indicates the Bonferroni threshold of 9.06. **c**, Stacked bar chart showing comparisons of the number of peak associations identified in GWASs (unadjusted –log_10_ (*P*_dis_) in the discovery cohort passing the threshold of 7.5 and unadjusted *P*_rep_ in the replication cohort passing the threshold of 0.05) for QSM versus T2* IDPs. **d**, Scatter plot showing the relationship between QSM right pallidum IDP versus allele count of rs13107325 (the strongest genetic association across all GWASs) using the discovery cohort (*n* = 19,720 individuals). The center dashed line depicts the group mean *χ* of each allele count with error bars indicating the standard deviation. **e**, Distribution of unadjusted –log_10_ (*P*) values of all peak associations identified in GWASs. The left *y* axis (blue line) is showing the total number of peak associations (both QSM and T2* IDPs) with –log_10_ (*P*) greater than the thresholds (*x* axis). The right *y* axis (orange line) is showing percentage of peak associations identified with QSM IDPs.

Following previous studies^5,6^, we use linkage score regression^39^ to estimate narrow sense heritability^40^ (*h*^2^) as the fraction of IDP variance that is explained by a linear combination of genetic variants. The theoretical range of *h*^2^ varies from 0 (independent of genotype) to 1 (entirely determined by genotype). Subcortical QSM and T2* IDPs are heritable, being more than 1 s.e. greater than 0 (Fig. 5a). In all but one IDP, QSM has higher heritability than T2*. *χ* in the putamen and substantia nigra showed the highest heritability (0.323–0.342), while T2* in the amygdala and accumbens showed two of the lowest heritability estimates (0.0357–0.0684). Heritability of all brain IDPs in UK Biobank was previously reported in the range of 0.000–0.405 (ref. ^6^). The heritability of *χ* in the right putamen (*h*^2^ = 0.342) is >98% among UK Biobank brain IDPs and roughly half the heritability of human height^40^.

### Genome-wide association studies (GWASs) of IDPs

We performed a GWAS for each QSM and T2* IDP following a previously described approach^5,6^ using the second release of over 90 million imputed genetic variants. Both aforementioned imaging confounds^11,23^ and additional genetic confounds^5^ were regressed out from the GWASs. Participants were divided into discovery (*n* = 19,720) and replication (*n* = 9,859) cohorts. The standard single-phenotype GWAS threshold (−log_10_ (*P*) = 7.5) and also a more stringent threshold after additional Bonferroni correction to account for the number of GWASs (18 × 2) performed (resulting in a Bonferroni threshold of −log_10_ (*P*) = 9.06) were used. We report the genetic variant with the strongest ‘peak’ association in each region of linkage disequilibrium (LD; Methods). Fig. 5b displays a Manhattan plot for the QSM right pallidum IDP. In total, QSM IDPs identified 292 peak associations (265 replicated), and T2* IDPs identified 225 peak associations (199 replicated). Fig. 5c provides a summary of peak associations from the set of GWASs. The strongest genetic association across all GWASs was found between the QSM right pallidum IDP and variant rs13107325, which is shown in Fig. 5d. Fig. 5e provides a summary of the distribution of −log_10_ (*P*) values of all peak associations identified in GWASs. Supplementary Information, section 5, includes Manhattan plots for all GWASs, and Supplementary Tables 2 and 3 provide the full list of peak associations.

We used the Peaks software^6^ to automatically generate clusters of peak associations between genetic variants and IDPs. A cluster is defined using the discovery cohort as a set of IDP–variant pairs for which all genetic variants are within a 0.25-cM distance of the top variant within the cluster. We classify a cluster as replicating if at least one of its IDP–variant pairs had nominal significance (*P* < 0.05) in the replication cohort. We used FUMA^41^ to map the genetic variants of each cluster to related genes.

We identified 89 distinct clusters, 80 of which replicated. Among the replicated clusters, 54 had associations with both QSM and T2* IDPs, 22 were unique to QSM IDPs, and 4 were unique to T2* IDPs. All clusters common to QSM and T2* IDPs replicated. Note that a cluster can include just a single genetic variant, which was the case for 11 replicated clusters. Table 2 provides a summary of 19 example clusters (the full list of clusters is given in Supplementary Table 4). Most replicating clusters are associated with genes, including 10 clusters with variants in exons (6 missense). Many clusters are associated with genes involved in functions with known relevance to tissue *χ*, including myelination, iron and calcium. Other clusters are associated with genes whose functions do not have expected relationships to tissue *χ*, including transcription factors, extracellular matrix and intracellular trafficking. Below, we describe select examples in detail.

**Table 2:** Information for the 19 example genetic clusters

Related function	Brain regions	Cluster no.	Lead IDP	*N*_pair_	rsID	MAF	Gene	Genetic function (cluster)	eQTL	−log_10_ (*P*_dis_)
Iron homeostasis	Pallidum, substantia nigra, putamen, caudate, hippocampus, thalamus, amygdala, accumbens	6	QSM pallidum (L)	15	rs11884632	0.256	*SLC40A1*	Downstream, intron	*WDR75*, *SLC40A1*, *C2orf88*, AC013439.4	71.271
		10	T2* putamen (L)	18	rs4428180	0.151	*TFP1*:*TF*	Intron	*TF*	42.458
		12	QSM SN (L)	7	rs9867473	0.571	*TFRC*	UTR3	*MUC20*, *MIR570*	14.562
		27	QSM putamen (R)	21	rs1800562	0.077	*HFE*	Exon (missense)	*RP1-221C16.8*, U91328.19	48.973
		66	QSM thalamus (L)	1	rs1131488	0.282	*HMBS*	Exon (synonymous)	*VPS11*, *HMBS*, *RP11-110I1.14*	7.8271
Metal ion transport	Caudate, putamen, pallidum, substantia nigra, accumbens, WMH	15	QSM pallidum (R)	18	rs13107325	0.070	*SLC39A8*	Exon (missense), intron	–	105.51
		47	QSM caudate (L)	24	rs4348791	0.395	*SLC39A12*	Upstream, intron	*SLC39A12*	100.55
Calcium homeostasis	Caudate, pallidum, substantia nigra, putamen	49	QSM caudate (R)	10	rs11012783	0.386	*CACNB2*	Intergenic	*CACNB2*, *SLC39A12-AS1*	75.579
		60	T2* caudate (R)	3	rs11013321	0.395	*CACNB2*	Intron	–	9.0318
		68	T2* putamen (R)	3	rs73192811	0.074	*TPCN1*	Intron	*RITA1*	10.406
		70	QSM putamen (R)	1	rs10842717	0.376	*ITPR2*	Intron	–	7.7763
		16	QSM pallidum (L)	12	rs13105682	0.057	*BANK1*	Intron	–	46.911
		18	QSM pallidum (L)	7	rs76062146	0.028	*BANK1*	Intron	–	14.353
		20	QSM pallidum (L)	2	rs71614699	0.012	*BANK1*	Intron	–	10.914
Myelin and glia	Thalamus, hippocampus, pallidum, putamen	11	QSM pallidum (L)	3	rs34457487	0.554	*MOBP*	Intron	–	15.932
		74	QSM putamen (L)	3	rs7203922	0.310	*PLLP*	Upstream, intron	–	10.575
		80	QSM thalamus (L)	4	rs1126642	0.043	*GFAP*	Exon (missense)	–	11.181
Extracellular matrix	Pallidum, substantia nigra, WMH	9	QSM pallidum (L)	5	^a^	0.017	*COL3A1*	Intron	–	9.5122
		25	QSM WMH	2	rs10052710	0.197	*VCAN*	Intron	–	18.745

Lead IDP and rsID represent the top IDP/variant pair in each group. *N*_pair_ indicates the number of IDP/variant pairs included in each genetic cluster. The –log_10_ (*P*_dis_) values provided are two-sided, unadjusted values from the main discovery cohort. Genetic function refers to the position of the cluster’s variants with regards to the corresponding gene.

SN, substantia nigra; R, right; L, left; UTR, untranslated region; MAF, minor allele frequency; eQTL, expression quantitative trait loci.

^a^2:189666936_ATTTGACACTCCTGATTCATCAC_A

#### Iron transport and homeostasis

**Fig. 6:**
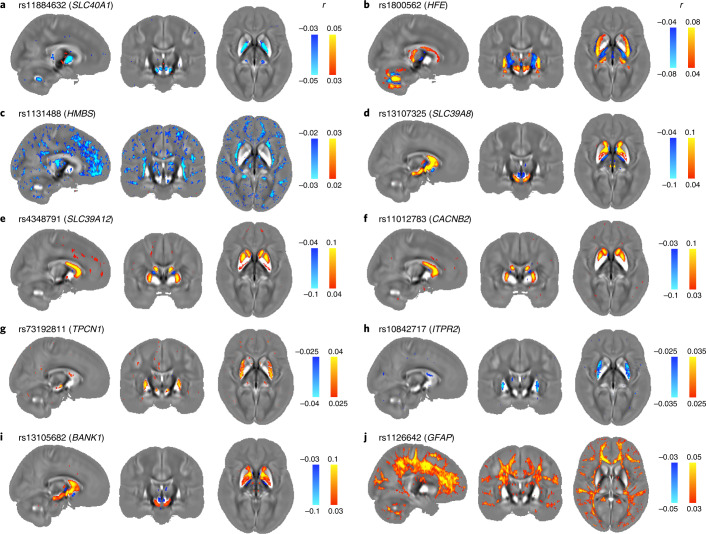
Voxel-wise association maps of top genetic variants of 10 genetic clusters with *χ* maps aligned in MNI space. The Pearson correlation *r* is shown as color overlay (red–yellow for positive *r* and blue for negative *r*) on the population average *χ* map. **a**, rs11884632 (*SLC40A1*) identifies the pallidum, subregions of the substantia nigra and thalamus, red nucleus and cerebellar nuclei. **b**, rs1800562 (*HFE*) identifies the putamen, red nucleus, cerebellar regions, subregions of the caudate, substantia nigra and thalamus. **c**, rs1131488 (*HMBS*) identifies subregions of the thalamus and dispersed white matter. **d**, rs13107325 (*SLC39A8*) identifies the caudate, substantia nigra and subregions of the pallidum. **e**, rs4348791 (*SLC39A12*) identifies the caudate and subregions of the putamen and pallidum. **f**, rs11012783 (*CACNB2*) identifies the caudate and subregions of the putamen. **g**, rs73192811 (*TPCN1*) identifies the putamen and subregions of the substantia nigra. **h**, rs10842717 (*ITPR2*) identifies the putamen. **i**, rs13105682 (*BANK1*) identifies the caudate, substantia nigra and subregions of the pallidum. **j**, rs1126642 (*GFAP*) identifies subregions of the thalamus and widespread white matter regions.

Multiple clusters are related to genes implicated in iron transport and homeostasis. Cluster 6 is composed of eight genetic variants related to the ferroportin gene (*SLC40A1*). The voxel-wise association map with rs11884632 (*SLC40A1*; Fig. 6a) includes the pallidum, subregions of the substantia nigra and thalamus, red nucleus and cerebellar nuclei. Ferroportin exports iron from cells, and mutations in *SLC40A1* lead to hemochromatosis (Mendelian Inheritance in Man (MIM) 606069). Three functionally related clusters identified associations with the transferrin gene (*TF*; cluster 10), the transferrin receptor gene (*TFRC*; cluster 12) and the homeostatic iron regulator gene (*HFE*; cluster 27, including a missense variant). The voxel-wise association map with rs1800562 (*HFE*; Fig. 6b) includes the putamen, red nucleus, cerebellar regions, subregions of the caudate, substantia nigra and thalamus. The voxel-wise association map with rs4428180 (*TF*) shows a similar pattern of association to rs1800562 (*HFE*), and rs9867473 (*TFRC*) shows a similar pattern of association to rs11884632 (*SLC40A1*; Supplementary Fig. 29). The TF protein delivers iron to proliferating cells via TFRC, an interaction that is modulated by the HFE protein to regulate iron absorption. Mutations in *HFE* lead to hereditary hemochromatosis (MIM 235200), while mutations in *TF* lead to hereditary atransferrinemia (MIM 209300). The variants we identified have been previously associated with transferrin levels^42^, iron biomarkers^43^ and Alzheimer’s disease^44^. Interestingly, associations with variants related to *SLC40A1* (iron export) and *HFE* (iron absorption) had opposite signs in corresponding regions, in line with their biological functions (Fig. 6a,b). Cluster 66 comprises a single association of QSM in the left thalamus with a potentially deleterious exonic variant (synonymous, CADD: 17.8) in the *HMBS* gene. The voxel-wise association map with this variant (rs1131488, *HMBS*) identifies subregions of the thalamus and dispersed white matter (Fig. 6c). *HMBS* encodes an enzyme from the heme biosynthetic pathway, and *HMBS* mutations are associated with acute intermittent porphyria and leukoencephalopathy, which exhibit MRI anomalies in the thalamus and cerebral white matter^45^.

#### Metal ion transporters

There are also clusters associated with genes from the SLC39 family of solute carriers, which transport divalent metal cations such as Zn^2+^ and Fe^2+^. Cluster 15 is composed of four genetic variants associated with QSM and T2* IDPs in multiple subcortical structures and WMHs. The top one (rs13107325) is a missense variant of *SLC39A8* (*ZIP8*). The voxel-wise association map with this variant (Fig. 6d) identifies the caudate, substantia nigra and subregions of the pallidum. *SLC39A8* encodes a transmembrane transporter protein for zinc and iron. This genetic variant has been linked to multiple traits, including metabolic syndrome^46^, Parkinson’s disease^47^, schizophrenia^47^, alcohol consumption^48^ and brain morphology^5,49^. Cluster 47 is composed of 11 genetic variants related to *SLC39A12* (*ZIP12*). The voxel-wise association map for the top variant (rs4348791; Fig. 6e) identifies the caudate and subregions of the putamen and pallidum. *SLC39A12* encodes a zinc/iron transmembrane transporter that is highly expressed in the brain; low expression of *SLC39A12* leads to impaired neural development^50^.

#### Calcium homeostasis

Seven clusters are related to calcium channels and regulation. Clusters 49 and 60 contain variants related to *CACNB2*. The voxel-wise association map with rs11012783 (*CACNB2*; Fig. 6f) identifies the caudate and subregions of the putamen. *CACNB2* encodes a subunit of voltage-gated calcium channels that regulate calcium influx from the extracellular space^51^. Variants in *CACNB2* have been associated with autism, bipolar disorder, depression and schizophrenia^52^. Cluster 68 includes two intronic variants in *TPCN1*, and cluster 70 includes one genetic variant in an intron of *ITPR2*. Voxel-wise maps exhibit a similar spatial pattern in the putamen for rs73192811 (*TPCN1*; Fig. 6g) and rs10842717 (*ITPR2*; Fig. 6h) but with opposite signs. Both *TPCN1* and *ITP2* encode calcium channels that control the release of calcium from intracellular spaces. Clusters 16, 18 and 20 are related to *BANK1*. The voxel-wise association map with rs13105682 (*BANK1*; Fig. 6i) reveals spatially contiguous associations in the caudate, substantia nigra and subregions of the pallidum. *BANK1* encodes a protein that regulates calcium mobilization from intracellular stores that is primarily related to the immune system but is also expressed in the brain. Variants in *BANK1* have been related to working memory task-related brain activation^53^.

#### Glia and myelin

We observed associations with genetic variants in two genes that encode structural constituents of myelin sheaths. Cluster 74 includes genetic variants related to *PLLP*, which encodes the myelin protein plasmolipin, and cluster 11 includes genetic variants located in an intron of *MOBP*, which encodes the myelin-associated oligodendrocyte basic protein. Cluster 80 includes a missense variant of *GFAP*, which encodes an intermediate filament protein that is highly specific for cells of astroglial lineage. The voxel-wise association map with rs1126642 (*GFAP*; Fig. 6j) reveals spatially contiguous associations in subregions of the thalamus and widespread white matter regions. Mutations in *GFAP* lead to Alexander disease, a genetic disorder characterized by fibrinoid degeneration of astrocytes, demyelination and white matter anomalies (MIM 203450). *GFAP* variations have also been associated with white matter microstructure phenotypes, cognitive and mental health traits^49^.

#### Extracellular matrix

Many genetic associations do not have obvious, direct links to magnetic susceptibility contrast. For example, cluster 25 includes a single genetic variant (rs10052710) located in an intron of *VCAN* that is associated with *χ* in WMHs (both with and without regressing out WMH volume). *VCAN* encodes versican, a major component of the extracellular matrix in multiple tissues, including the brain, and is highly expressed in the brain during development^54^. In addition to its structural role, versican can also interact with inflammation and the immune response^54^, and its expression is altered in MS lesions^55^. This and other variants in *VCAN* have been previously associated with multiple brain phenotypes, in particular to white matter microstructure metrics derived from diffusion MRI^5,49^.

## Discussion

### QSM in population imaging

Unlike the other brain imaging modalities in UK Biobank, which have been (or are being) collected or collated previously in thousands of participants^56–58^, QSM has previously been limited to smaller-scale studies. This UK Biobank QSM resource is approximately two orders of magnitude greater than the largest existing QSM dataset^59^. The number of participants, coupled with the breadth of linked data, including genetics, extensive phenotyping and health outcomes, is expected to open up new avenues of investigation for QSM. At the time of scanning, most UK Biobank participants are largely healthy, with the cohort age range designed to reflect a broad range of health outcomes in the coming decades. Hence, this cohort is particularly appropriate for identifying early markers of age-associated pathology. For example, in the imaged cohort, thousands of participants are expected to develop Alzheimer’s and Parkinson’s disease by 2030 (ref. ^60^). A notable future QSM resource is the Rhineland Study, which is collecting swMRI phase images in 20,000 individuals at 3T (ref. ^57^); while QSM has not yet been produced in this study, the complementary age range (≥30 years old) will ultimately enable new investigations on its own and in combination with UK Biobank data.

Producing accurate and reproduceable *χ* maps for a dataset at this scale requires a robust, fully automated QSM processing pipeline. We investigated available algorithms for each processing stage to identify an optimal QSM pipeline for the UK Biobank swMRI data (Supplementary Information, section 1.1). This includes a new algorithm to automatically detect and exclude voxels with extremely large phase variance, a major source of imaging artifacts (details provided in Supplementary Information, section 1.1). In our evaluations, we observed considerable variability of *χ* estimates across different combinations of background field removal and dipole inversion algorithms. Dissemination of our pipeline will thus be crucial for harmonization of our IDPs with data acquired in new settings, such as clinical scanners. This will, for example, enable stratification of individuals using classifiers^61^ or nomograms^62^ derived from UK Biobank data. To date, two ongoing coronavirus disease 2019 brain imaging studies have already adopted our QSM pipeline (C-MORE/PHOSP and COVID-CNS)^63^ to process their brain swMRI data.

Using the two-timepoint data from 1,368 participants (Supplementary Fig. 10), we found high cross-scan correlations (*r* > 0.8) with *χ* in four subcortical regions (the putamen, caudate, substantia nigra and pallidum). These correlations are higher than the corresponding T2* IDPs (0.6 < *r* < 0.8). These four regions show the highest heritability among all QSM and T2* IDPs (Fig. 5a), also representing some of the highest heritability values across all brain IDPs in UK Biobank^6^. Notably, these four regions also have the highest *χ* among all ROIs and are reported to contain the highest iron concentrations in the brain^64^. *χ* has demonstrated a strong, positive linear relationship with iron concentration in postmortem brain tissue^26^. These observations suggest that in structures where iron is the dominant *χ* source, QSM provides an accurate, reproduceable proxy for tissue iron levels.

### Phenotypic and genetic associations with QSM

The associations described in detail above represent approximately 5% of identified phenotypic associations, leaving a rich set of phenotypes to be explored further. Some of these phenotypes have previously established links to *χ* measures in the brain (for example, cognitive scores^65^), but many phenotypes lack an obvious interpretation. In many cases, the relationship may be indirect; for example, the apparent relationship between *χ* in the caudate and putamen with use of sun/UV protection could relate to socioeconomic factors^1^. In addition to the extensive literature looking at QSM in neurological conditions, there is a more limited literature in mental health conditions, such as depression^66^. Our results supplement this literature, identifying associations between mental health related risk factors, including seen doctor (general practitioner) for nerves, anxiety, tension or depression, risk taking and ever taken cannabis (Supplementary Fig. 27).

We described example associations for 19 of the 89 genetic clusters that have a plausible link between gene function and tissue *χ*. However, we also observed many associations with genetic variants related to biological functions that are not known to be directly related to tissue *χ*. This includes immune response, regulation of gene expression and cell function (Supplementary Information, section 6). This rich set of associations will require additional studies to understand the genetic architecture of magnetic susceptibility in the brain.

In addition to providing insight into ‘true’ associations, voxel-wise maps can help identify spurious associations. For example, several apparent associations at the IDP level seem to be driven by structural atrophy. While all ROI-based IDPs have the potential to be sensitive to atrophy, QSM represents an extreme of image contrast, with opposite signs of *χ* in gray and white matter. This causes small errors in gray–white boundaries to be amplified into detectable apparent alterations in *χ*. For example, QSM in the substantia nigra has an apparent association with total bone mineral density, but the voxel-wise map exhibits thin, intense associations with a positive–negative pattern at the gray–white boundary that likely reflects atrophy (Supplementary Fig. 26a,b). A similar spatial pattern of opposing positive and negative correlations exists for BMI in the primary motor and somatosensory areas (Supplementary Fig. 26c,d).

### Iron and calcium in quantitative swMRI

Overall, the most consistent and strongest pattern of associations identified in our study relate swMRI to iron, both phenotypically (for example, hemoglobin content) and genetically (for example, iron homeostasis). Iron is involved in many fundamental biological processes in the brain, including neurotransmitter production, myelin synthesis and metabolic processes^67^. Iron homeostasis is essential to normal brain function, with elevated iron causing oxidative stress and cellular damage^67^. It remains unclear whether abnormal iron accumulation in the brain is a cause or consequence of neurodegeneration in diseases such as Alzheimer’s, Parkinson’s and MS^67^.

We also identified multiple associations with variants in genes encoding calcium channels. While calcification has been shown to alter tissue *χ* (*χ* values in calcified lesions have been validated using computed tomography attenuation values^68^), little is known about the impact of other calcium forms on tissue *χ*. Calcium is essential for many aspects of cell function, including division, differentiation, migration and death^51^ as well as neurotransmitter and hormone release. Perturbations in calcium homeostasis, including dysregulation of calcium channel activity, have been reported in many neurodegenerative disorders^69^.

These results suggest that the UK Biobank resource can play a key role in the development of swMRI-based biomarkers of iron and calcium. In particular, UK Biobank has unique value for investigating early, asymptomatic disease in individuals who go on to develop neurological conditions. swMRI measures could provide predictive markers for preventative stratification, treatment monitoring and imaging-based screening.

### T2* and QSM in UK Biobank

In UK Biobank, the swMRI protocol was only allocated for 2.5 min, placing limitations on the achievable data quality. While swMRI protocols in research studies often can acquire isotropic resolution and multiple echoes, the UK Biobank swMRI protocol was limited to two echoes with anisotropic resolution (0.8 × 0.8 × 3 mm). The use of anisotropic voxels is in line with established practice for detecting cerebral microbleeds^70^, which was the primary motivation for including swMRI in UK Biobank. The further ability to process these data for T2* and QSM is a valuable but secondary aim for which the protocol incurs compromises. QSM involves a single-parameter fit (*χ*) that can be calculated from the phase at a single echo, although our pipeline explicitly uses both echoes. One limitation of the T2* estimates in UK Biobank is that they are based on the minimum of two echo times, making estimates more sensitive to noise than protocols with more echoes. The use of the thick slices in UK Biobank swMRI scans might lead to systematic underestimation of absolute *χ* compared to using thinner slices^71^. Nevertheless, we found moderate to good reproducibility with QSM IDPs. In all but one ROI, QSM IDPs showed higher reproducibility than T2* IDPs.

Our reproducibility analysis showed lower cross-scan consistency for T2* IDPs than for QSM IDPs (Supplementary Fig. 10b). In previous literature^72,73^, T2* data calculated using multiecho (≥5) protocols showed similar reproducibility to QSM data; one possible reason for this difference is the use of a dual-echo protocol in UK Biobank.

Estimation of T2* is also biased by the presence of macroscopic field gradients induced by air/tissue interfaces or poor shim quality^22^. This bias is exacerbated by the thick slices used in UK Biobank^22^. If not corrected, this can lead to spurious correlations driven by participant-wise variations in field homogeneity rather than tissue *χ* (Supplementary Fig. 15). We introduced deconfounding of macroscopic field gradient for T2* IDPs (see Supplementary Information, section 2). As expected, background field gradients do not correlate with our *χ* estimates (Supplementary Fig. 16), and this confound is not needed for QSM.

Despite these potential shortcomings of swMRI in UK Biobank, our results demonstrate that QSM and T2* contribute unique information, as reflected in distinct patterns of associations. Because diamagnetic and paramagnetic constituents manifest differently in QSM and T2* data, a combination of QSM and T2* data may be able to disentangle co-occurring changes of tissue iron and myelin content in neurodegeneration^74^.

QSM is an emerging technique with considerable potential to quantify important tissue constituents. The associations reported here for *χ* estimates with a broad range of phenotypes and genetic variants provide good agreement with the literature and demonstrated the complementarity of *χ* to the existing UK Biobank neuroimaging resource. This new QSM resource provides opportunities for new investigations that could lead to new application of QSM in basic and clinical neuroscience.

## Methods

### Ethics

UK Biobank has approval from the North West Multi–centre Research Ethics Committee to obtain and disseminate data and samples from the participants (http://www.ukbiobank.ac.uk/ethics/), and these ethical regulations cover the work in this study. Written informed consent was obtained from all participants.

### MRI data acquisition and participant information

UK Biobank will scan 100,000 individuals over the course of the study. Here, we used data from 35,885 participants in the UK Biobank early 2020 release who had swMRI data collected. Participants were 53.11% female and 45–82 years old (64.04 ± 7.5 years old) at the time of imaging. Of these participants, 1,368 (51.39% female, 65.22 ± 7.2 years old at the time of second imaging) were recruited for a repeat scan approximately 2 years (2.25 ± 0.12 years) after the first imaging session. A detailed overview of the neuroimaging acquisition protocols used in UK Biobank brain imaging has been previously described^1^.

swMRI scans were acquired at four different sites using identical 3T Siemens Skyra MRI scanners (software platform VD13) with 32-channel head receive coils. swMRI data were acquired using a three-dimensional (3D) dual-echo gradient echo sequence with the following parameters: voxel size = 0.8 × 0.8 × 3 mm^3^, matrix size = 256 × 288 × 48 (whole-brain coverage), echo times (*TE*_1_/*TE*_2_) = 9.4/20 ms, repetition time (TR) = 27 ms, in-plane acceleration = 2 and total scan time = 2 min 34 s. Magnitude and phase data from each receive channel were saved for offline coil combination, as described below.

The UK Biobank brain imaging protocol includes T1- and T2-weighted structural acquisitions that are used in our processing pipeline^11^. Specifically, the T1-weighted structural scan is used to align participants into a standard space atlas for the definition of ROIs and other masks, and T2 fluid attenuated inversion recovery (FLAIR) data are used to generate ventricle masks used in QSM referencing. Specifically, brain region masks and image registration references were derived from the T1-weighted structural scans acquired using a 3D MPRAGE protocol (voxel size = 1 × 1 × 1 mm^3^, matrix size = 208 × 256 × 256, inversion time (TI)/TR = 880/2,000 ms, in-plane acceleration = 2, total scan time = 4 min 54 s)^11^. Ventricle and WMH masks were derived from T2-weighted FLAIR scans (3D SPACE, voxel size = 1.05 × 1 × 1 mm^3^, matrix size = 192 × 256 × 56, TI/TR = 1,800/5,000 ms, in-plane acceleration = 2, total scan time = 5 min 52 s)^11^. Both T1- and T2-weighted structural data were processed using the UK Biobank image processing pipeline^11^.

The quality control (QC) pipeline developed for UK Biobank brain imaging^11^ was applied to all individuals included in this study. Several QC measures related to T1-weighted, T2 FLAIR and swMRI data were used here. For example, individuals were excluded if registration from T1 space to standard space failed or had unusable T2 FLAIR data, for example, due to excessive head motion, atypical structure and/or anatomical abnormalities. Full details of the QC pipeline have been previously described^11^. Finally, 35,273 individuals were selected in this study whose data were deemed suitable for QSM analysis based on QC measures.

### QSM processing pipeline

QSM consists of several steps (Fig. 1a). For each step, many different algorithms have been proposed^12,75,76^. To ensure the robustness of our QSM pipeline and select the optimal pipeline for the UK Biobank protocol, we performed extensive evaluations of established algorithms for each step of the QSM pipeline. Details of these evaluations are described in Supplementary Information, section 1.1. The final QSM pipeline for UK Biobank swMRI data is as follows.

To generate a map of the image phase, we first need to combine phase images from individual coil channels. The challenge is that each coil channel has a different unknown phase offset that will lead to phase cancellation (and resulting artifacts) if it is not first removed. Our pipeline combines phase images across channels using the MCPC-3D-S approach^17^, generating channel-combined phase images free from any phase cancellation artifacts for each echo.

Estimation of *χ* using dipole inversion (see below) is highly sensitive to masking. The brain masks used in the standard UK Biobank image processing pipeline^14^ were refined for QSM to detect and remove voxels in the vicinity of sinus cavities (Supplementary Fig. 3a). These regions have extremely strong field variations due to the air/tissue interface that induce artifacts in the *χ* maps. For each echo, we generated a phase reliability map as described in Supplementary Information, section 1.1, which is used to refine the brain mask. These refined masks remove regions with sharp phase gradients, which introduces artifacts to *χ* estimates in neighboring tissue and where *χ* cannot be reliably estimated.

The channel-combined phase images were unwrapped using a Laplacian-based algorithm provided by the STI Suite toolbox^18^. The unwrapped phase images were subsequently combined into a single phase data via a weighted sum^19^ over the two echoes to increase signal-to-noise ratio, with weighting factors $\frac{{TE_{\mathrm{1/2}} \cdot e^{ - TE_{\mathrm{1/2}}/{\mathrm{T}}2^ \ast }}}{{\mathop {\sum }\nolimits_{n = 1}^2 TE_{{\mathrm{}}n} \cdot e^{ - TE_{{\mathrm{}}n}/{\mathrm{T}}2^ \ast }}}$ (ref. ^19^) (T2* was set as 40 ms for all participants). This effectively weights each echo by its predicted signal-to-noise ratio.

The echo-combined phase data was then filtered to remove the background field contributions. Our pipeline uses the V-SHARP algorithm^20^ in the STI Suite toolbox with a maximum kernel size of 12 mm. The phase reliability mapping step described in Supplementary Information, section 1.1, was applied to the brain mask output by V-SHARP, excluding voxels that had phase reliability values lower than 0.7 for the first echo or 0.6 for the second echo (empirically determined). The *χ* map was generated from the V-SHARP filtered phase data using the refined brain mask. Dipole inversion was performed using the iLSQR algorithm^21^ in the STI Suite toolbox.

QSM provides a measure of relative, rather than absolute, *χ* (ref. ^77^). The presence of an unknown offset in the estimated map for any individual is problematic for comparison of *χ* values across participants. Therefore, the final step of our QSM pipeline is to subtract a reference *χ* value from the *χ* map for each participant. As described in Supplementary Information, section 1.2, we compared the use of three widely used reference regions^77^: (1) mean *χ* across the whole brain, (2) CSF and (3) a white matter region (forceps minor). We evaluated each reference region using cross-scan correlations from 1,368 participants who were scanned at two timepoints. These analyses indicated that CSF-referenced QSM IDPs showed the highest cross-scan consistency. The *χ* map in the original swMRI space was referenced to CSF by subtracting this reference *χ* value.

### IDP generation

In this study, QSM-based IDPs were generated relating to eight subcortical structures (accumbens, amygdala, caudate, hippocampus, pallidum, putamen, substantia nigra and thalamus, left and right) and WMH lesions. Masks for each subcortical structure in T1 space (excluding the substantia nigra) have been previously generated using FMRIB’s Integrated Registration and Segmentation Tool (FIRST)^78^ as part of the UK Biobank image processing pipeline. Masks for the substantia nigra used in this study were derived from a substantia nigra atlas^79^ in MNI152 space. Masks for white matter and WMH lesions (derived from Brain Intensity AbNormality Classification Algorithm (BIANCA) processing^11,80^) were provided to us in T1 space from the UK Biobank image processing pipeline alongside white matter masks generated using FMRIB’s Automated Segmentation Tool (FAST). CSF-referenced *χ* maps were transformed to both individual’s native T1 space and MNI152 space using the provided transformations for each individual from UK Biobank^11^.

FIRST-generated subcortical masks were eroded (two-dimensional 3 × 3 kernel) and used to extract subcortical regions on CSF-referenced *χ* maps in the T1 space of each individual. The median *χ* value (across extracted voxels) was calculated as a separate IDP for each of the left/right subcortical regions. The substantia nigra mask was used to extract the substantia nigra region on CSF-referenced *χ* maps in the MNI152 space of each individual. To account for the morphological variations of substantia nigra across participants, the substantia nigra mask was refined by excluding voxels that had negative *χ* values, with median *χ* values subsequently calculated as a separate IDP for left and right substantia nigra.

For the WMH IDP, the WMH mask was first used to extract the mean *χ* value in lesions. We then extracted the median *χ* value from normal-appearing white matter, defined as the intersection of FAST- and BIANCA-based white matter mask with the WMH voxels removed. The difference between the estimated *χ* values of WMH and normal white matter was calculated as an IDP. This approach aims to isolate *χ* properties that are unique to lesions as opposed to more global properties of white matter *χ*.

While most of the QSM IDPs use the same spatial ROIs as existing T2* IDPs, the previous pipelines did not include T2* of substantia nigra or WMH. We thus generated four new T2* IDPs for substantia nigra (left and right) and WMH using the same approach described above. This enabled a direct comparison between QSM and T2* for 18 IDPs.

### Outlier and confound removal

Each IDP’s *N*_participants_ × 1 vector had outliers removed. Outliers were defined as being greater than six times the median absolute deviation from the median. The remaining distribution of IDPs was then quantile normalized, resulting in it being Gaussian distributed with a mean of 0 and an s.d. of 1.

A recently expanded set of imaging confounds has been proposed in UK Biobank brain imaging^11,23^, including age, head size, sex, head motion, scanner table position, imaging center and scan date-related slow drifts. The effect of two example demographic variables (age and sex) on QSM IDPs is demonstrated in Supplementary Information, section 1.3. Unless stated otherwise, the full set of imaging confounds were regressed out from the quantile normalized IDPs before any further analyses. This was crucial to avoid biased or spurious associations between IDPs and other non-imaging measures^1,23^.

As reported in the literature, T2* estimates are biased by the presence of macroscopic field gradients (for example, induced by the air/tissue interface), particularly when imaging voxels are large (in our case, using thick slices)^22^. To reduce such confounding effects on association analyses with UK Biobank T2* IDPs, we first modeled the relationship between macroscopic field gradients and R2* (1/T2*) measurement errors using both simulated and UK Biobank data^22^. This produced a macroscopic field gradient confound variable specific to each participant and brain region, which was generated and regressed out from T2* IDPs. No association was found between the field gradient confounds and QSM IDPs as expected, and thus, this particular confound was only applied to T2* data. Details of this additional deconfounding for T2* IDPs are described in the Supplementary Information, section 2.

We also observed strong negative correlations between QSM/T2* WMH IDPs and the WMH volume IDP provided by UK Biobank (Fig. 1d). This may indicate partial volume effects on the *χ* and T2* estimates within lesions. QSM and T2* WMH IDPs were thus additionally processed by regressing out the WMH volume.

### Associations between IDPs and non-imaging measures

We investigated 17,485 phenotypes from UK Biobank. Each of the 17,485 phenotypes used here had data from at least 40 individuals. The phenotypes spanned 17 groups of variable types, including early life factors (such as maternal smoking around birth), lifestyle factors (such as diet, alcohol and tobacco consumption), physical body measures (such as BMI, bone density and blood assays), cognitive test scores, health outcomes (such as clinical diagnosis (ICD10) and operative procedure (OPCS4) codes) and mental health variables (such as bipolar and major depression status)^81^. These variables were automatically curated using the FMRIB UK Biobank Normalisation, Parsing And Cleaning Kit (FUNPACK) software to ensure that all phenotype variables (both quantitative and categorical) were numeric vectors and that resulting correlation coefficients were easy to interpret.

To investigate pairwise associations between IDPs and phenotypes, univariate statistics were performed using Pearson correlation across all QSM/T2* measures (including 16 subcortical IDPs and 2 WMH IDPs with/without regressing out WMH volume) and 17,485 phenotypes (quantile normalized and fully deconfounded). As UK Biobank phenotypes have varying amounts of missing data, the full set of associations of phenotypes against IDPs had widely varying degrees of freedom. Therefore, it is important to consider *P* values (and not just correlation *r*); *P* values were calculated and used to identify the strongest associations. Bonferroni multiple comparison correction across the full set of 629,460 (17,485 × 36) correlations was applied, resulting in a –log_10_ (*P*) threshold of 7.10 (for *P*_corrected_ < 0.05). Additionally, a less conservative option for multiple comparison correction is FDR^82^, which for a 5% FDR resulted in a –log_10_ (*P*) threshold of 3.13.

### Associations between IDPs and genetic variants

We performed GWASs (univariate correlations) for all QSM and T2* IDPs by following a previously described approach^5,6^. This was performed using the Spring 2018 UK Biobank release of imputed genetic data. From all individuals with an available *χ* map, we selected a subset of 29,579 unrelated individuals with recent UK ancestry (to avoid confounding effects from population structure or complex cross-participant covariance). We divided this set into a discovery cohort of 19,720 individuals and a replication cohort of 9,859 individuals. We applied QC filtering to the genetic data, including a minor allele frequency of ≥0.01, an imputation information score of ≥0.3 and a Hardy–Weinberg equilibrium *P* value of ≥10^−7^, which resulted in a total of 17,103,079 genetic variants (which are primarily single-nucleotide polymorphisms). IDPs had both imaging and genetic confounds regressed out as performed in Elliott et al.^5^, including the above-described imaging confounds and 40 population genetic principal components (supplied by UK Biobank). IDPs were normalized (resulting in zero mean and unit variance) after the original Gaussianization and deconfounding. GWAS was performed using the BGENIE software^83^.

Manhattan plots for each of the GWASs were produced, plotting the –log_10_ (*P*) value for each genetic variant. The standard single-phenotype GWAS threshold (–log_10_ (*P*) = 7.5)^5,6^ as well as an additional Bonferroni multiple comparison-corrected (accounting for 36 GWASs) threshold (–log_10_ (*P*) = 9.06) are shown in the plots. Peak associations with –log_10_ (*P*) values exceeding 7.5 were extracted and annotated using a method described in Elliott et al.^5^; that is, in a region of high LD, we only report the genetic variant with the highest association with the IDP because the associations in the local region are most likely all due to a single genetic effect^5,6,83^.

After performing the GWAS, we used Peaks software^6^ to automatically generate clusters of peak associations for all IDPs as previously described^5,6^. A cluster is a set of IDP/variant pairs for which all variants are within a 0.25-cM distance of the IDP/variant pair with the highest –log_10_ (*P*) value in the cluster. Additionally, we used FUMA^41^ to map genetic variants identified in peak associations to related genes and to identify expression quantitative trait loci and chromatin mappings/interactions for these variants.

Following the approach described previously^5^, we examined the heritability (*h*^2^) of each subcortical IDP using LD score regression (LDSC)^39^ on all available individuals (combining discovery and reproduction cohorts). LD scores were sourced from the European population of the 1,000 Genomes Project^84^. Due to limitations in 1,000 Genome Project LD score files, the X chromosome is not considered in this analysis.

### Voxel-wise associations with phenotypes and genetic variants

The MNI152 space versions of CSF-referenced *χ* maps from all individuals were also combined into a four-dimensional (4D) MNI152 *χ* matrix (of size 182 × 218 × 182 × *N*_participants_). Each of the voxel vectors in the 4D MNI152 *χ* matrix had outliers removed and was quantile normalized and fully deconfounded.

We performed voxel-wise correlations between the *χ* maps and non-imaging measures (both phenotypes and genetic variants) that had been identified to have significant associations with QSM-based IDPs. Interrogating *χ* maps at a voxel-wise level can provide further insight into the spatial localization of associations and can potentially identify additional associated areas, which were not captured by the original IDPs either because a given brain region was explicitly not included or because heterogeneity within a larger brain region diluted an association with a subregion. Univariate Pearson correlations were performed between fully deconfounded *χ* data vectors (voxels across the 4D MNI152 *χ* matrix) and phenotype or genetic variant data vectors, resulting in 3D correlation maps.

### Reporting summary

Further information on research design is available in the Nature Research Reporting Summary linked to this article.

## Online content

Any methods, additional references, Nature Research reporting summaries, source data, extended data, supplementary information, acknowledgements, peer review information; details of author contributions and competing interests; and statements of data and code availability are available at 10.1038/s41593-022-01074-w.

## Supplementary information

Supplementary Information

Reporting Summary

Supplementary Table Supplementary Tables 1–4.

## Data Availability

The UK Biobank brain imaging data from the early 2020 release of 35,885 participants (and 1,368 participants’ repeat imaging data) were used in this study. Permission to use the UK Biobank Resource was obtained via material transfer agreement (https://www.ukbiobank.ac.uk/media/yfob3gln/access_031_f-applicant-mta-data-only-v1-1.pdf) as part of Data Access Application 8107. All imaging data (including raw images, derived maps and IDPs), phenotypes and genetics data are made available by UK Biobank via their standard data access procedure (see http://www.ukbiobank.ac.uk/register-apply). Information on average time from application submission to data release can be found at https://www.ukbiobank.ac.uk/enable-your-research/apply-for-access. The full set of GWAS summary statistics generated in this study can be downloaded from https://www.fmrib.ox.ac.uk/ukbiobank/gwas_resources/index.html. LD measurements used in this study were obtained from the 1,000 Genomes Project (https://data.broadinstitute.org/alkesgroup/LDSCORE/).

## Code availability

The image processing pipelines of the MRI data in the UK Biobank project can be found at http://www.fmrib.ox.ac.uk/ukbiobank or https://git.fmrib.ox.ac.uk/falmagro/UK_biobank_pipeline_v_1. Custom-written MATLAB code for QSM processing is freely available at https://git.fmrib.ox.ac.uk/cwang/uk_biobank_qsm_pipeline and will be added to the next version of the core UK Biobank brain imaging processing pipeline.

The following software packages were used in this work:

• MATLAB R2019a

• FMRIB Software Library (FSL) v6.0, https://fsl.fmrib.ox.ac.uk/fsl/fslwiki

• FSLNets v0.6, https://fsl.fmrib.ox.ac.uk/fsl/fslwiki/FSLNets

• STI Suite v3.0 (including Laplacian phase unwrapping, V-SHARP and iLSQR algorithms), https://people.eecs.berkeley.edu/~chunlei.liu/software.html

• MCPC-3D-S v1.6, https://github.com/korbinian90/ASPIRE

• MEDI toolbox v2020, http://pre.weill.cornell.edu/mri/pages/qsm.html

• Mixture modeling v1.0, https://github.com/allera/One_Dim_Mixture_Models

• FUNPACK v1.0, https://git.fmrib.ox.ac.uk/fsl/funpack

• Peaks v1.0, new software for extracting clusters from multiphenotype GWASs, https://github.com/wnfldchen/peaks

• bgenie v1.3, software for efficient GWASs on high-dimensional phenotype data, https://jmarchini.org/bgenie/

• LDSC v1.0.1, software for heritability analysis from summary statistics (linkage score regression), https://github.com/bulik/ldsc/
