# Supplementary material for: Phenotypic and genetic associations of quantitative magnetic susceptibility in UK Biobank brain imaging
Source: Nat Neurosci. 2022 May 23;25(6):818–31. doi: 10.1038/s41593-022-01074-w (PMC9174052; doi:10.1038/s41593-022-01074-w)
Supplement: Supplementary file 1 — Supplementary Information [file 41593_2022_1074_MOESM1_ESM.pdf]

nature portfolio

https://doi.org/10.1038/s41593-022-01074-w

Supplementary information

Phenotypic and genetic associations of quantitative magnetic susceptibility in UK Biobank brain imaging

In the format provided by the authors and unedited

Supplementary material

Section 1: QSM pipeline evaluations

1.1 Evaluation of processing algorithms

Quantitative susceptibility mapping (QSM) consists of several steps, including combination of phase data from individual channels, unwrapping of channel-combined phase data, removal of macroscopic ('background') field inhomogeneities, and estimation of voxel-wise magnetic susceptibility (χ) through dipole inversion. For each step, many different algorithms have been proposed. To ensure the robustness of our QSM pipeline and to select the optimal pipeline for the UK Biobank protocol, we carried out extensive evaluations of established algorithms (from widely-used and publicly-available toolboxes) for each step using data from 150 subjects selected to represent the age range of the whole cohort (45-82yo). Visual inspections and quantitative analyses (including group mean, cross-subject variance of spatial χ maps and IDPs) were used in the evaluations. The details of QSM pipeline evaluations are summarized in the Supplementary Table 5:

Supplementary Table 5. Summary of QSM pipeline evaluations

Name of the processing step	No. of subjects used	Algorithms evaluated	Evaluation methods
multi-channel coil-combination	150	1. phase difference<br>2. MCPC-3D-S	visual inspections[1]
phase unwrapping	150	1. PRELUDE<br>2. Laplacian-based	visual inspections
background field removal	150	1.V-SHARP<br>2. PDF<br>3. LBV<br>4. iRSHARP<br>5. iHARPERELLA	1. visual inspections<br>2. quantitative analyses[2]
dipole inversion	150	1. iLSQR<br>2. STAR-QSM<br>3. MEDI<br>4. fast-TFI	1. visual inspections<br>2. quantitative analyses
referencing	1,368 with repeat scans	1. whole brain<br>2. cerebrospinal fluid<br>3. a white matter region (forceps minor)	cross-scan consistency (correlation)

[1] Visual inspection includes observations of remaining large-scale field inhomogeneities (due to incomplete background field removal) and streaking artefacts. [2] quantitative analyses include cross-subject consistency of both spatial χ maps (in standard space) and χ values in regions of interest (subcortical IDPs).

Combination of multi-channel phase data

Robust combination of multi-channel phase data is crucial for performing QSM. Different channels have different phase offsets ($\varphi_{0,j}$) associated with the respective coil-sensitivity field.

If left unaccounted for, these offsets will lead to signal cancelation and open-ended fringe lines in the resulting coil-combined phase maps. As UK Biobank swMRI protocol collects two echoes (TEs = 9.4 and 20 ms) without performing reference scans (for coil sensitivity estimation), only a handful of algorithms can be used for coil combination. In this work, we compared two different coil combination methods: (1) phase difference and (2) MCPC-3D-S[1].

Although MCPC-3D-S is slower than the phase difference method (due to multiple additional steps including phase unwrapping, here performed using PRELUDE[2]), it generates coil-combined phase data with higher SNR verses the phase difference method, as shown in **Supplementary Fig. 1**.

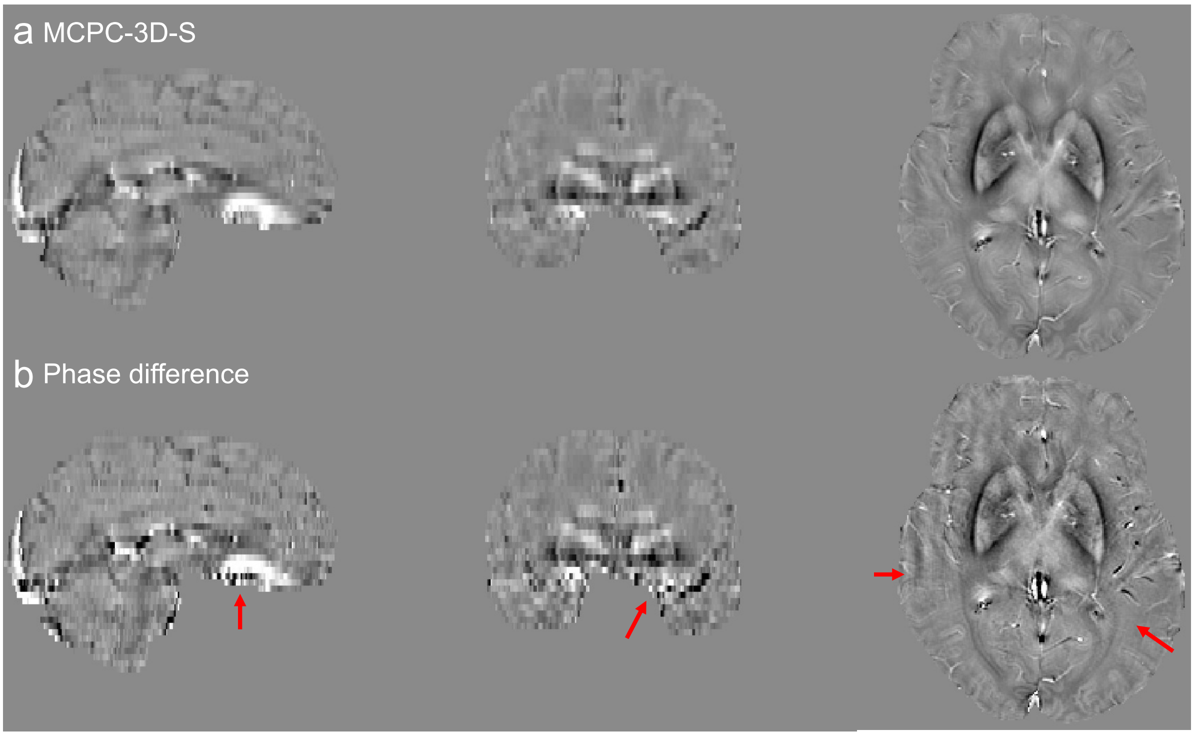

Supplementary Figure 1 Example filtered phase data from a single UK Biobank subject using MCPC-3D-S **(a)** and phase difference **(b)** channel combination. Overall, MCPC-3D-S (a) generated images with higher SNR and fewer imaging artefacts (red arrows) than phase difference (b).

Unwrapping of channel-combined phase

There are a number of algorithms which have been developed for phase unwrapping. In this study, we compared a path-based (FSL's PRELUDE [2]) and a Laplacian-based[3] (from the STI suite toolbox https://people.eecs.berkeley.edu/~chunlei.liu/software.html) algorithm. Overall, the Laplacian-based algorithm demonstrated rapid phase unwrapping with smoother phase variance in (noisier) regions with large phase jumps verses PRELUDE, as shown in **Supplementary Fig. 2**. Laplacian-based algorithms perform a degree of background field removal when unwrapping phase maps, which restricts their use when quantitative phase values are required (e.g. the coil-combination step). However, as unwrapped phase data are subsequently filtered in QSM (to remove background field contributions), this does not restrict their use for generating χ maps.

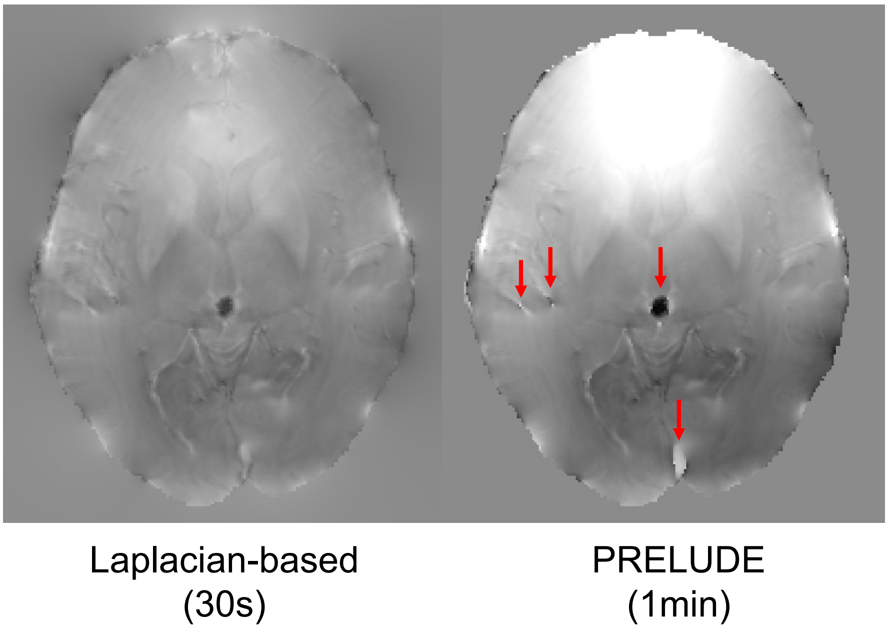

Supplementary Figure 2 Unwrapped phase image from an example UK Biobank subject. Red arrows indicate boundaries with large phase jumps in the unwrapped phase map using PRELUDE, which are not present using the Laplacian-based algorithm.

Phase reliability maps

Unreliable phase estimates can lead to artefacts and large-scale image inhomogeneities on resulting χ maps. To address this, a phase reliability map was estimated per subject to identify and remove these voxels. As shown in Supplementary Fig. 3a, channel-combined phase images were first converted to complex data (assuming unit magnitude values) and convolved with a 3D spherical kernel. This spherical kernel was normalised to account for the fraction of each voxel contained in the kernel, i.e., voxels at the kernel centre have higher intensity while voxels at kernel edge have lower intensity. The phase reliability map was subsequently derived by calculating the magnitude of the convolved complex data: regions of strong phase variation were indicated by low magnitude values due to phase cancellation from the convolution step, while regions with relatively homogeneous phase had magnitude values close to one, as shown in **Supplementary Fig. 3**. The threshold of the phase reliability map for each echo was empirically determined (0.6 for first echo and 0.5 for second echo before background field removal; 0.7 for first echo and 0.6 for second echo before dipole inversion), to exclude unreliable voxels (predominantly in the vicinity of sinus cavities). An additional step was then applied to the refined brain mask to fill any isolated holes (in 3D) in the middle of the bran that were not connected to the sinus cavities using the MATLAB function "imfill". The performance of phase reliability masking is demonstrated in Supplementary Fig. 3b. Based on a similar concept, a recently-proposed approach aims to suppress severe artefacts caused by these voxels at brain edges by restoring reliable phase information, rather than removing voxels[5].

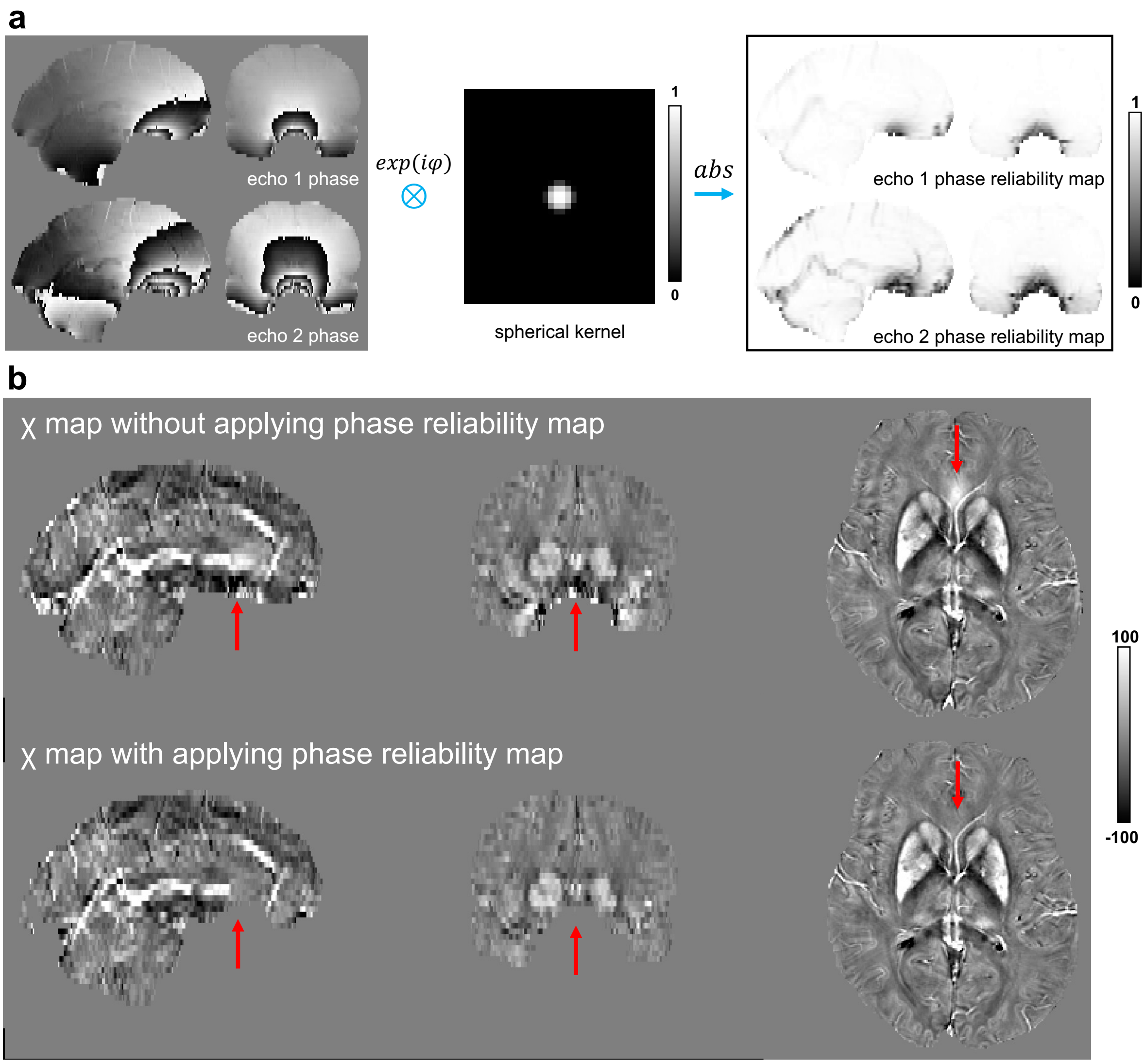

Supplementary Figure 3 (a) Phase reliability map pipeline for each echo in a single UK Biobank subject. ⊗ represents convolution in complex space, where phase data (φ) were converted to exp ($i \cdot \varphi$). Phase reliability map was calculated as the magnitude (absolute value) of the convolved complex data **(b)** Example χ map generated without (top) and with (bottom) the phase reliability map correction. Red arrows indicate regions with strong phase variations that induced large field inhomogeneities. The phase reliability correction removes voxels with large phase variations at the brain edge, resulting in χ maps with fewer artefacts.

Background field removal and dipole inversion

Removal of background field contributions and subsequent dipole inversion are the final two steps required to generate χ maps, with a number of different algorithms proposed for each step. Here, we evaluated combinations of these algorithms based on the final χ map. These evaluations used both quantitative and qualitative metrics, including the observation of large-scale inhomogeneities and streaking artefacts on χ maps, and cross-subject consistency of both χ maps (in standard space) and χ values in regions of interest (subcortical structures).

We compared 5 different background field removal algorithms (V-SHARP[6], PDF[7], iHARPERELLA[8], iRSHARP[9] and LBV[10]), 3 different dipole inversion algorithms (iLSQR[11], STAR-QSM[12] and MEDI[13]) and 1 single-step background field removal and dipole inversion algorithm (fast TFI[14]) resulting in the evaluation of 16 ($3 \times 5 + 1$) different combinations.

Background field removal:

Here we present evaluations using group averaged χ maps over the 150 subjects for the different background field removal algorithms. Although evaluations were performed using all of the proposed dipole inversion algorithms, for conciseness here we display results using iLSQR only. Details of our dipole inversion algorithm evaluations are provided in the following section.

Supplementary Fig. 4 compares V-SHARP with PDF, iHARPERELLA, iRSHARP, and LBV. Overall, we found that V-SHARP provided the best performance, generating χ maps without observable large-scale inhomogeneities and a low cross-subject standard deviation on resulting χ maps.

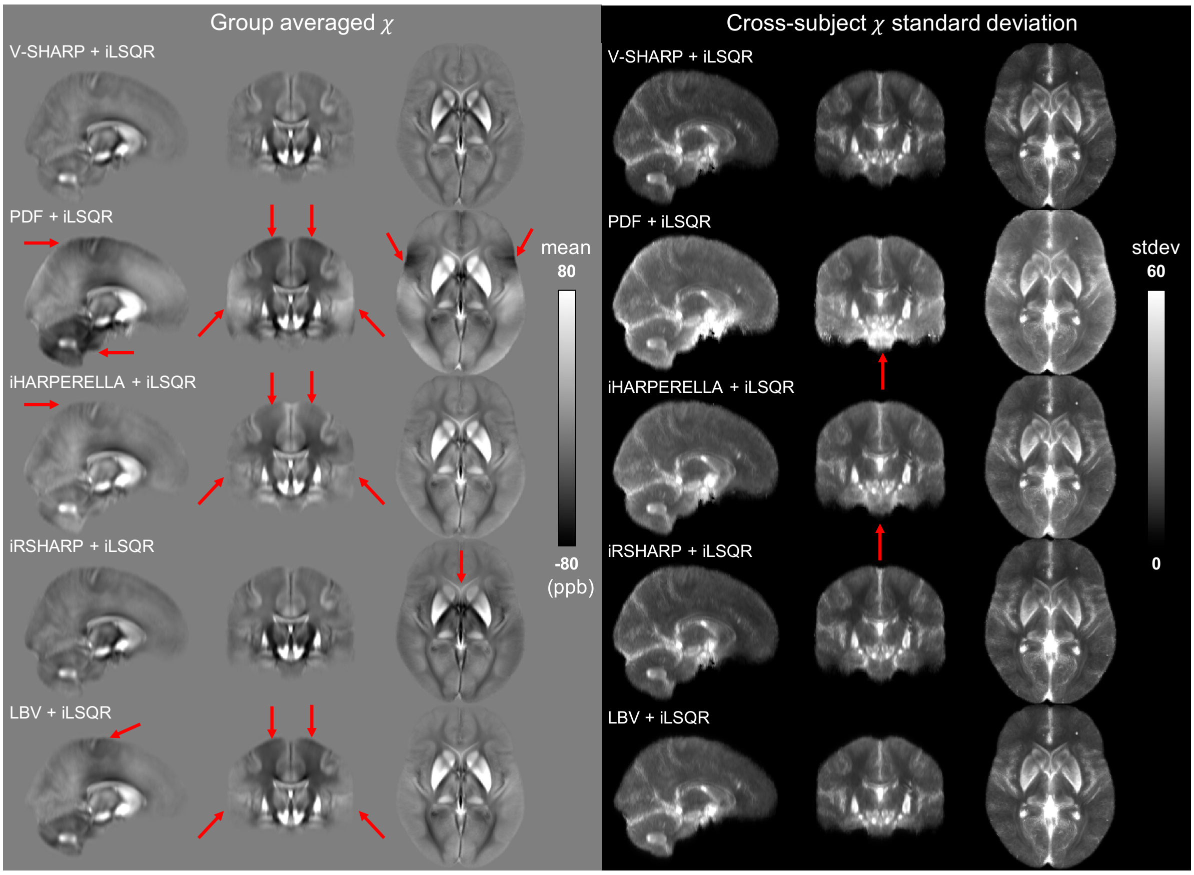

Supplementary Figure 4 Group averaged χ maps (left) and standard deviation (stdev) maps (right) from 150 subjects. χ maps were produced using V-SHARP/PDF/iHARPERELLA/iRSHARP/LBV and iLSQR. Red arrows highlight regions of large-scale inhomogeneity (consistent across subjects) and cross-subject variation on resulting χ maps when compared to V-SHARP and iLSQR. Although iRSHARP and V-SHARP are derived from the same technique (SHARP), for UK Biobank data the V-SHARP implementation was found to remove more field inhomogeneities in brain regions in close vicinity to our regions of interest, including the caudate, putamen and pallidum.

Dipole inversion algorithms:

Here we present evaluations using group averaged χ maps over the 150 subjects for the different dipole inversion algorithms. Although evaluations were performed using all of the proposed background field removal algorithms, again for conciseness here we display results using V-

SHARP only, which was the method adopted for background field removal (**Supplementary Fig. 4**). Comparisons between V-SHARP + iLSQR, STAR-QSM and MEDI, in addition to the single-step fast TFI algorithm are shown in **Supplementary Fig. 5**.

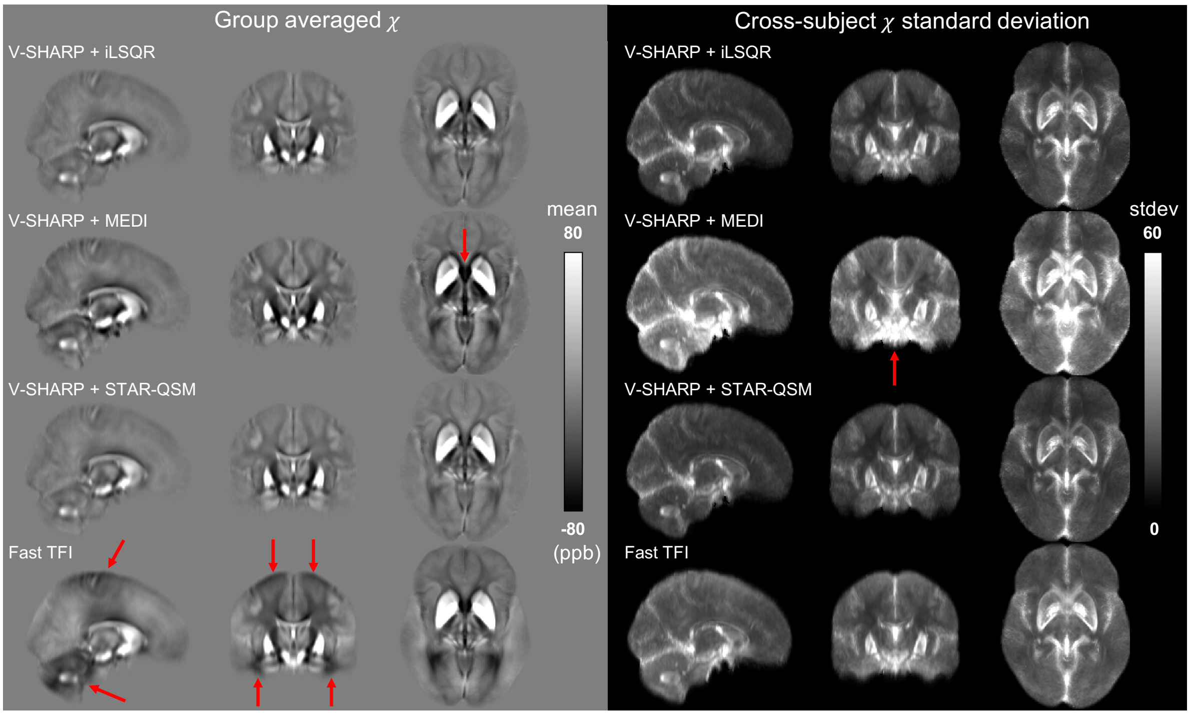

Supplementary Figure 5 Group averaged χ maps (left) and standard deviation (stdev) maps (right) from 150 subjects. χ maps were produced using V-SHARP and iLSQR/STAR-QSM/MEDI, in addition to the single-step fast TFI algorithm. Red arrows highlight regions of large-scale inhomogeneity (consistent across subjects) and cross-subject variation on resulting χ when compared to V-SHARP and iLSQR. The group averaged χ maps using iLSQR and STAR-QSM visually appeared of very similar quality.

STAR-QSM and iLSQR produced similar appearing χ maps. We therefore carried out an expanded comparison using phase data without the phase reliability map correction, to simulate the circumstances where voxels with large field variations remained in the dataset. **Supplementary Fig. 6** demonstrates that iLSQR outperformed STAR-QSM in such a situation.

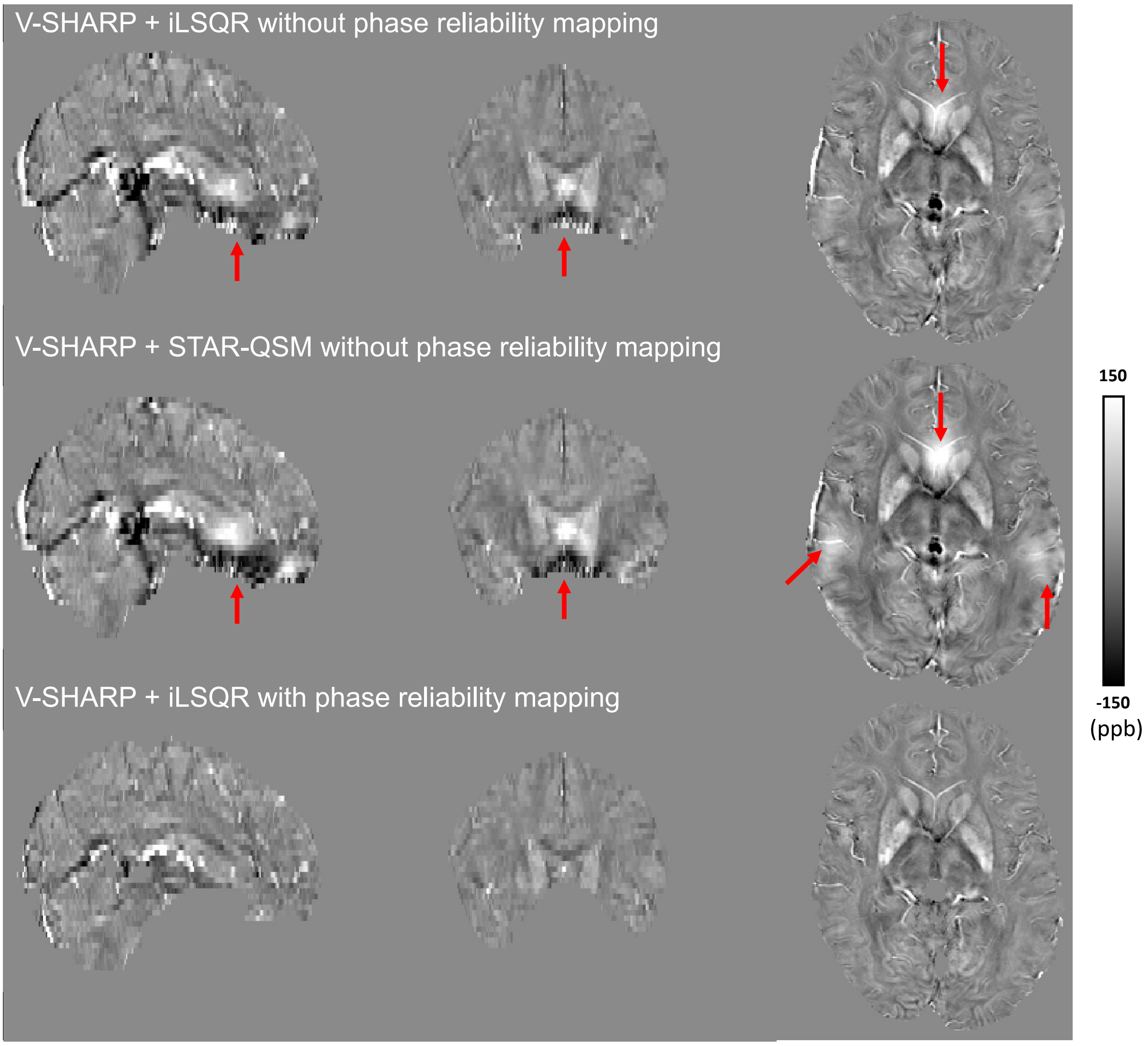

Supplementary Figure 6 Example χ map from a single UK Biobank subject using V-SHARP and iLSQR/STAR-QSM without the phase reliability map correction (**Fig S3**). Overall, iLSQR demonstrated improved suppression of streaking artefacts and image inhomogeneities on resulting χ maps (top) verses STAR-QSM (middle), important if the phase reliability correction failed to remove all voxels with unreliable phase information. Remaining V-SHARP and iLSQR image inhomogeneities are subsequently eliminated with the phase reliability correction (bottom).

Finally, we evaluated the different background field and dipole inversion algorithms by calculating the median χ in 14 subcortical ROIs (thalamus, caudate, putamen, pallidum, hippocampus, amygdala, accumbens, left and right separated) (**Supplementary Fig. 7**). Although different combinations of algorithms showed varying degrees of artefacts on χ maps (**Supplementary Figs. 4 and 5**), median χ measures in the subcortical ROIs are broadly in similar ranges. The combination of V-SHARP and iLSQR generally showed the smallest within-ROI variance.

Our comparisons determined that the combination of MCPC-3D-S, Laplacian-based phase unwrapping, masking with the phase reliability correction, V-SHARP and iLSQR was the optimal pipeline for UK Biobank swMRI data.

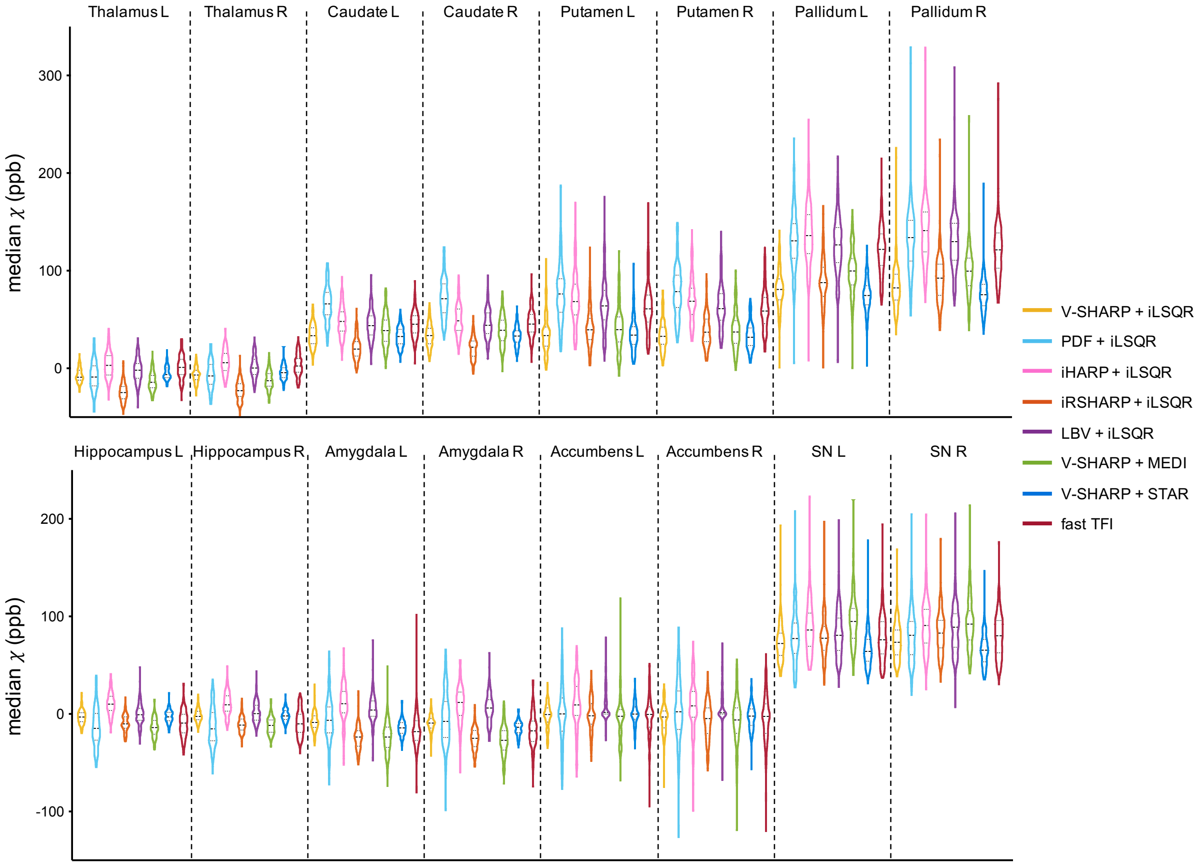

Supplementary Figure 7 Comparison of median χ values derived from χ maps for 8 combinations of algorithms. Here, violin plots are used to visualize the distribution of median χ estimates in 16 ROIs of 150 subjects. For each ROI, different combinations generated median χ values covering a broadly similar range. The combination of V-SHARP and iLSQR generally showed the smallest within-ROI variance.

1.2 evaluations of reference region for χ maps

The ROI masks for cerebrospinal fluid (CSF) and the white matter region (forceps minor) are shown in **Supplementary Fig. 8.**

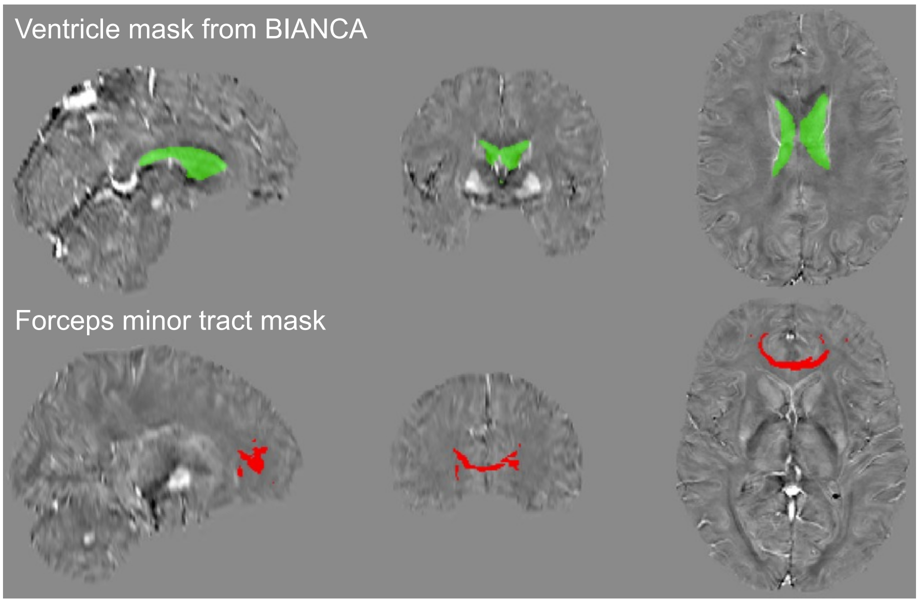

Supplementary Figure 8 Ventricle and forceps minor tract masks used for the CSF and white matter reference, overlayed on an example χ map (single subject). Ventricle masks for each subject were previously generated as part of UK Biobank, extracted from the T2 FLAIR data using BIANCA[16]. The forceps minor tract mask was derived from the diffusion MRI data using AutoPtx[17].

The χ distribution in the ventricle mask used for CSF referencing (**Supplementary Fig. 9a**) contained a bimodal (assuming to be Gaussian and inverse gamma) distribution, which likely corresponds to CSF and calcification of the choroid plexus. To extract the CSF component of this distribution, we used an in-house mixture modelling algorithm[18] (**Supplementary Fig. 9b**). The mean of the central Gaussian distribution was considered to represent CSF voxels, and was used as the CSF χ reference.

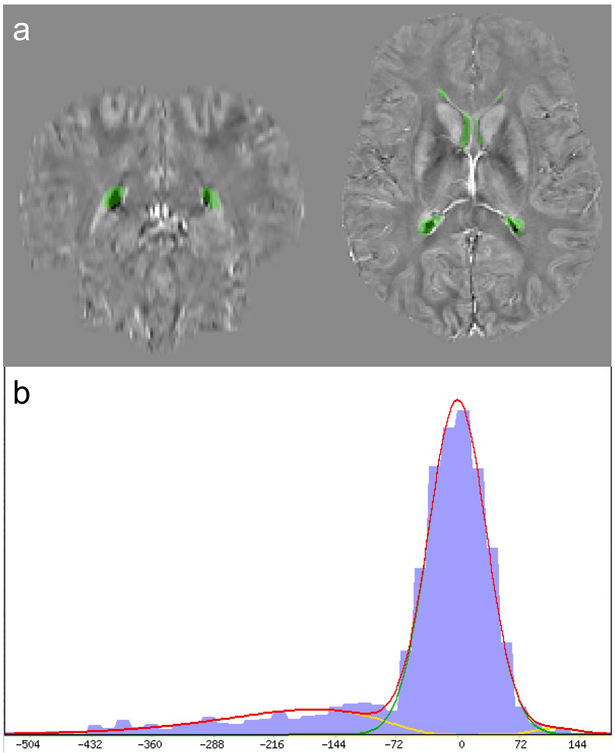

Supplementary Figure 9 (a) Example ventricle mask overlaid on top of a χ map for a single subject. **(b)** distribution of χ in the ventricle mask. The negative voxels likely correspond to calcification of

the choroid plexus, resulting in the left "tail" (negative inverse gamma) of the χ distribution. The CSF reference χ estimate was measured as the mean of the main Gaussian distribution.

Evaluation of the three reference regions were performed using UK Biobank subjects who had undergone scanning at two time points (1,368 subjects in total) with second imaging session performed approximately 2 years (2.25±0.12y) after the first imaging session. Specifically, we compared cross-scan consistency of χ maps referenced to these three different regions (**Supplementary Fig. 10**) with the assumption that an ideal reference would maximise the cross-scan consistency for χ estimates.

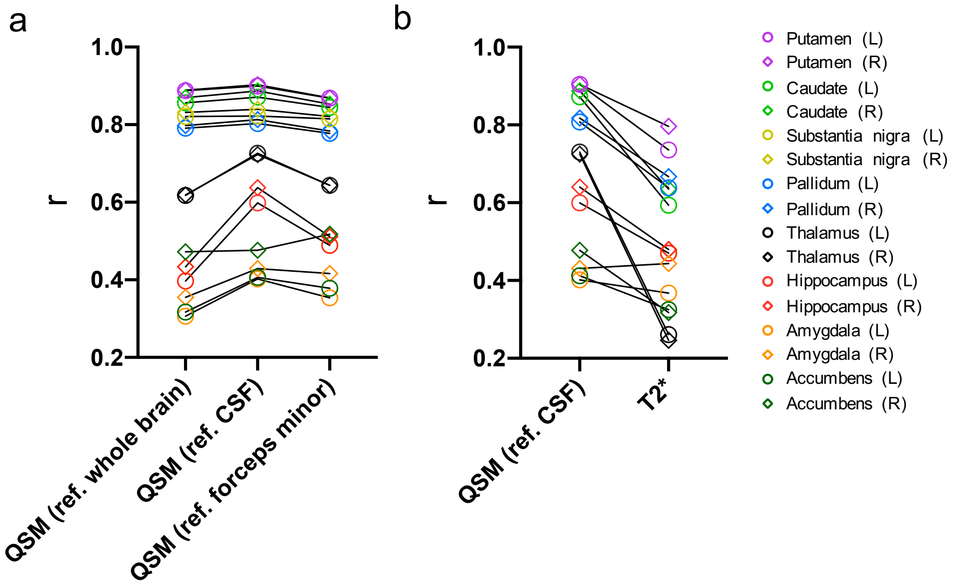

Supplementary Figure 10 Correlation between QSM IDPs obtained at the first and second imaging visit using n=1,368 subjects. (a) Overall, the CSF referenced IDPs yielded the highest Pearson correlation r values (most evident in the thalamus and hippocampus), indicating that CSF is the most robust reference among the three regions. (b) QSM IDPs typically display higher reproducibility verses the T2* IDPs. Note "QSM (ref. whole brain) was calculated by subtracting the mean of whole brain from the χ map generated by iLSQR.

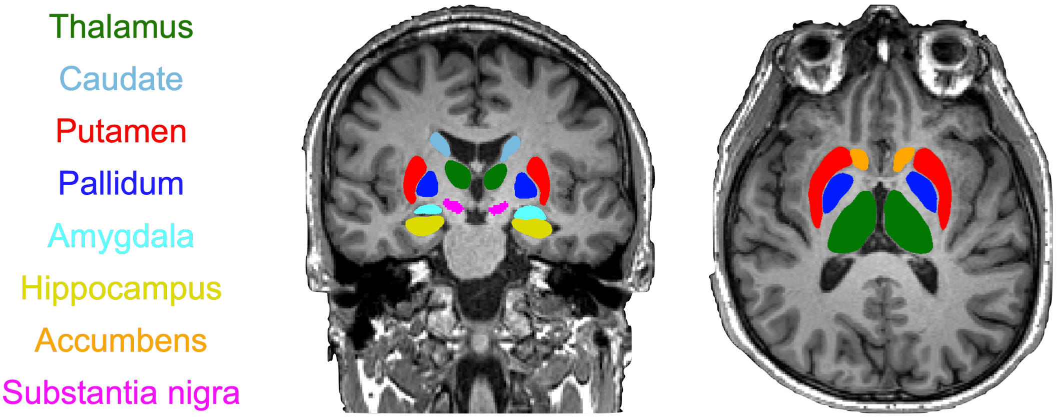

Supplementary Figure 11 Illustration of subcortical ROI masks in T1 space for an example subject. Note that mask for substantia nigra was derived from an MNI atlas and the IDP for substantia nigra was calculated in MNI space. The mask was transformed to T1 space here for visualisation in a single subject.

Section 1.3: Effects of age and sex on subcortical QSM IDPs

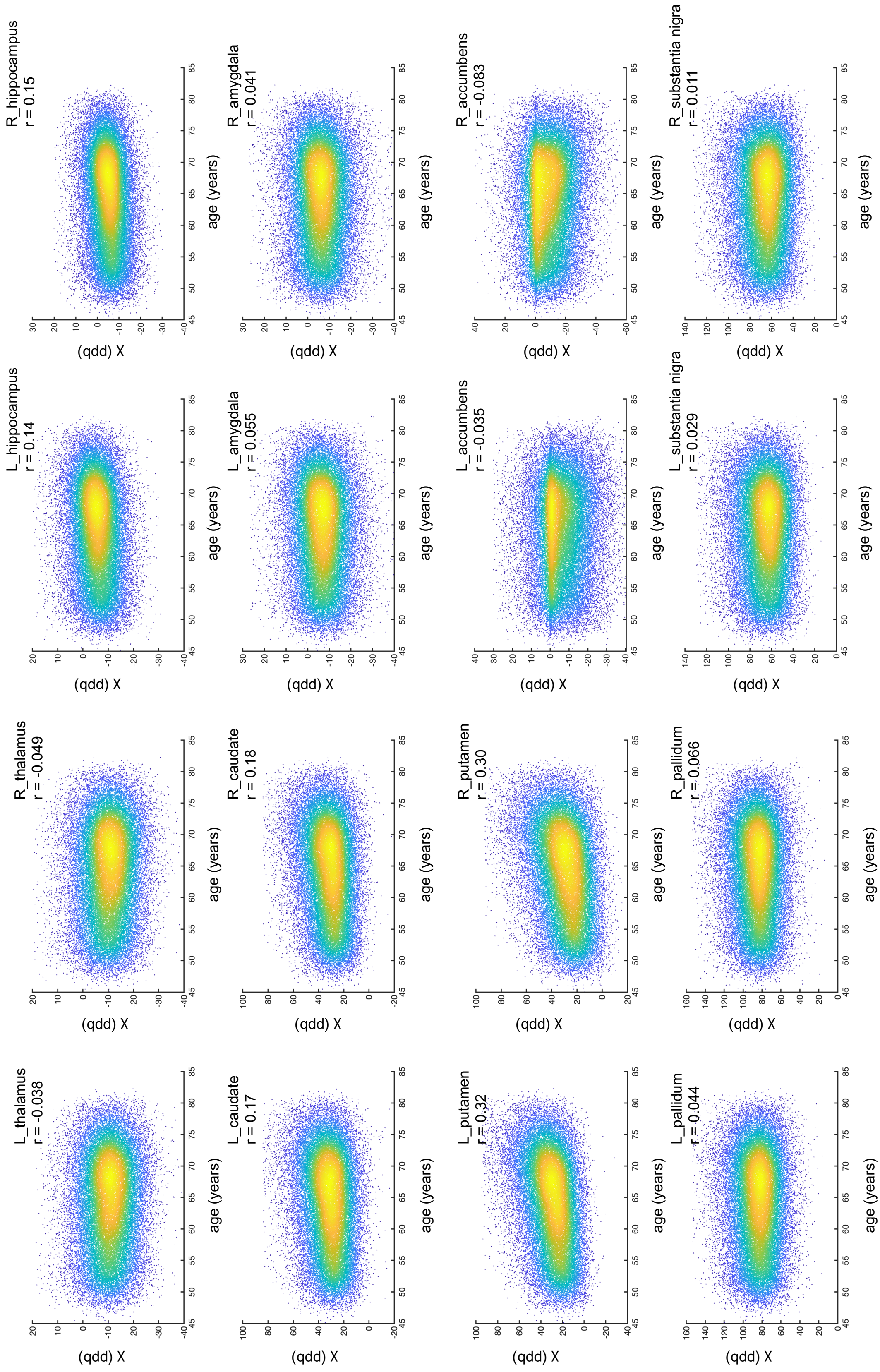

Supplementary Figure 12 Pearson correlations between subcortical QSM IDPs (in ppb, without deconfounding) and age using a total of n=35,273 subjects. Putamen showed the strongest association with age which is in line with a previous study by Persson et al.[19].

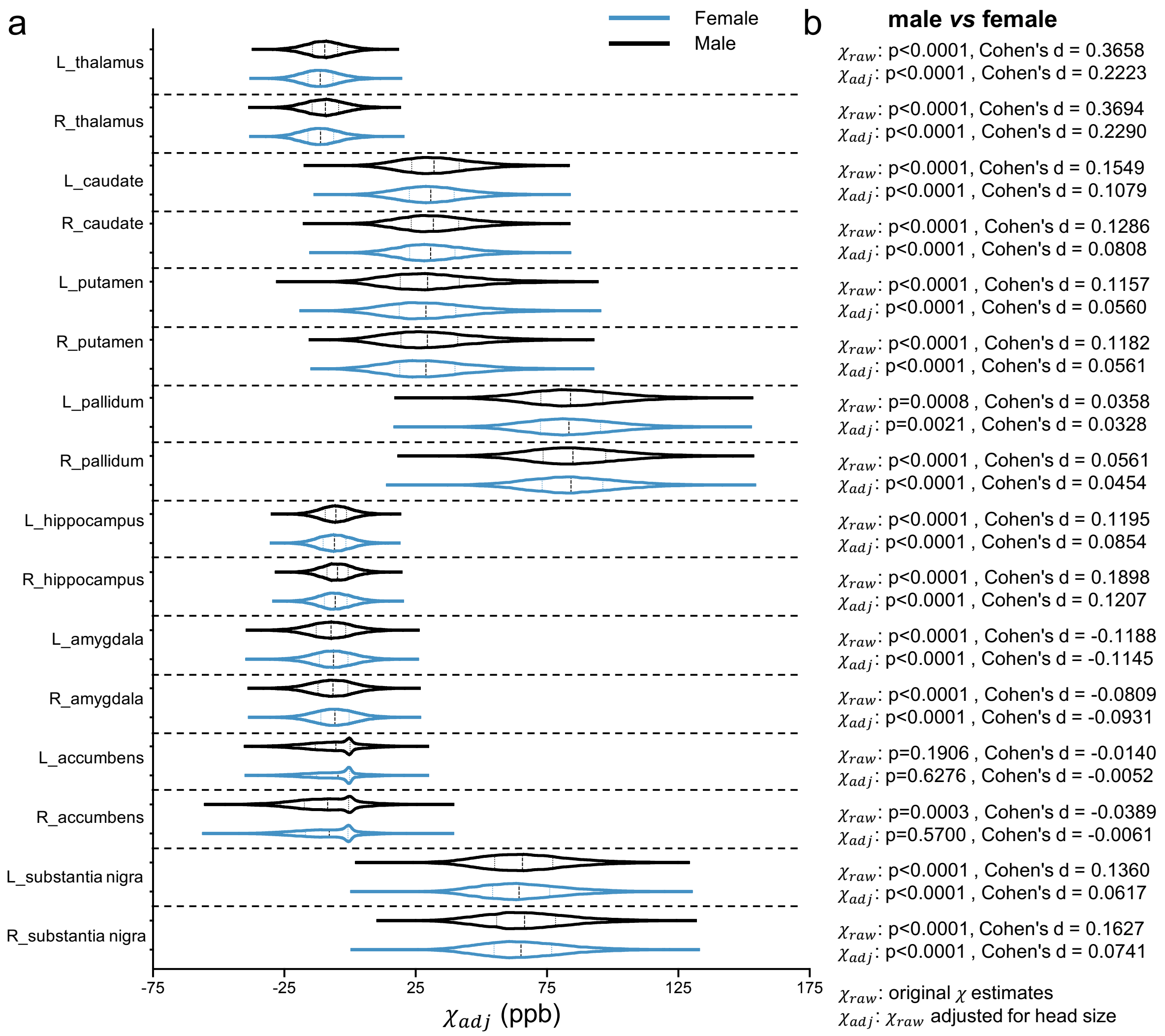

Supplementary Figure 13 Effect of sex on 16 subcortical QSM IDPs (in ppb) plotted using a violin plot (a). Note the QSM IDPs presented here were adjusted for head size *(χ_{adj})* which was reported as a major confound driving the apparent differences between sexes. The central line represents median, the two surrounding lines represent first and third quartiles and the width of the curve corresponds with the approximate frequency of data points in each region. Effect sizes (Cohen's d) and p values are reported in (b) for group differences in both raw QSM IDPs and IDPs adjusted for head size. The reported p-values are from two-tailed t test using a total of n=35,273 subjects.

Section 2: Additional deconfounding for T2* IDPs

Estimation of a biologically-meaningful T2* reflecting cellular compartments is confounded by the presence of macroscopic field gradients induced by air/tissue interfaces or poor magnetic field shim quality[20]. In large-cohort epidemiological studies, this could lead to spurious correlations driven by subject-wise variations in field homogeneity (for example, geometry of the sinuses, or other anatomical structures outside the brain), rather than cellular phenomena. In the presence of macroscopic field gradients, the gradient-echo signal can be modelled as[20]:

$$\mathrm{S}(\mathrm{TE}, r_0) = \int_{R^3} S_0\, e^{-R2^*(r)TE} e^{j2\pi\gamma\Delta B_0(r)TE} \cdot SRF(r - r_0)dr \qquad [5]$$

where $S(TE, r_0)$ is the measured signal at echo time TE and location r_0, $R2^*(r)$ is the spatial distribution of $R2^*$ $(= 1/T2^*)$, $\gamma\Delta B_0(r)$ is the magnetic field offset (in Hz), and $SRF(r - r_0)$ is the spatial response function (SRF) of a voxel centered at r_0. As spatial resolution along the slice direction (z-dimension) is typically lower than the in-plane dimensions, Eq. [5] can be simplified as[20]:

$$S(TE, z) = \int_R S_0\, e^{-R2^*(z)TE} e^{j2\pi\gamma\Delta B_0(z)TE} \cdot SRF(z - z_0)dz \qquad [6]$$

Here, we describe our approach to estimate and deconfound for the impact of macroscopic field gradients on the UK Biobank T2* analysis.

Simulated impact of macroscopic field gradients – 2D and 3D acquisitions:

Simulations are based on the procedure by Hernando et al.[20]. Assuming a linear through-slice field variation ($\Delta B_0(z) = \Delta B_0(z_0) + G[z - z_0]$), for a 2D acquisition the SRF is a boxcar function (**Supplementary Fig. 14a**, first row – left). This leads to:

$$S(TE, z) = S_0 e^{-R2^*TE} e^{j2\pi\gamma\Delta B_0(z_0)TE} \cdot \Delta z \cdot sinc(\gamma G_z \Delta z TE) \qquad [7]$$

where G_z is the macroscopic field gradient along the slice direction and Δz is the slice thickness. For 2D acquisitions with an ideal slice profile, the measured signal is thus modulated by $sinc(\gamma G \Delta z TE)$ (**Supplementary Fig. 14a**, first row - right), with larger field gradients (G) or thicker slices (Δz) leading to faster signal decay. For 3D acquisitions, the SRF is a sinc-like function (**Supplementary Fig. 14a**, second row - left), leading to a measured signal modulated by a boxcar-like function (**Supplementary Fig. 14a**, second row - right).

Simulated impact of macroscopic field gradients – UK Biobank swMRI protocol:

The UK Biobank swMRI protocol is a 3D sequence with 3 mm slice thickness, incorporating k-space filtering (windowing) to remove Gibbs ringing and improve SNR. This leads to an SRF consisting of a rapidly-decaying sinc-like function (**Supplementary Fig. 14a**, third row - left), with the measured signal modulated by a smoothed boxcar-like function (**Supplementary Fig. 14a**, third row - right).

To estimate the bias on T2* estimates on UK Biobank swMRI data, we first simulated the impact of macroscopic field gradients using the UK Biobank swMRI protocol (**Supplementary Fig. 14a**, third row). We subsequently used these simulations to model the relationship between estimated R2* (s^{-1}) (as demonstrated by Eq. [5-7], calculation in R2* is more straightforward than in T2*) and Gz (Hz/mm) (**Supplementary Fig. 14b**), using this relationship to estimate parameters to inform the UK Biobank T2* deconfounding (**Supplementary Fig. 14c**).

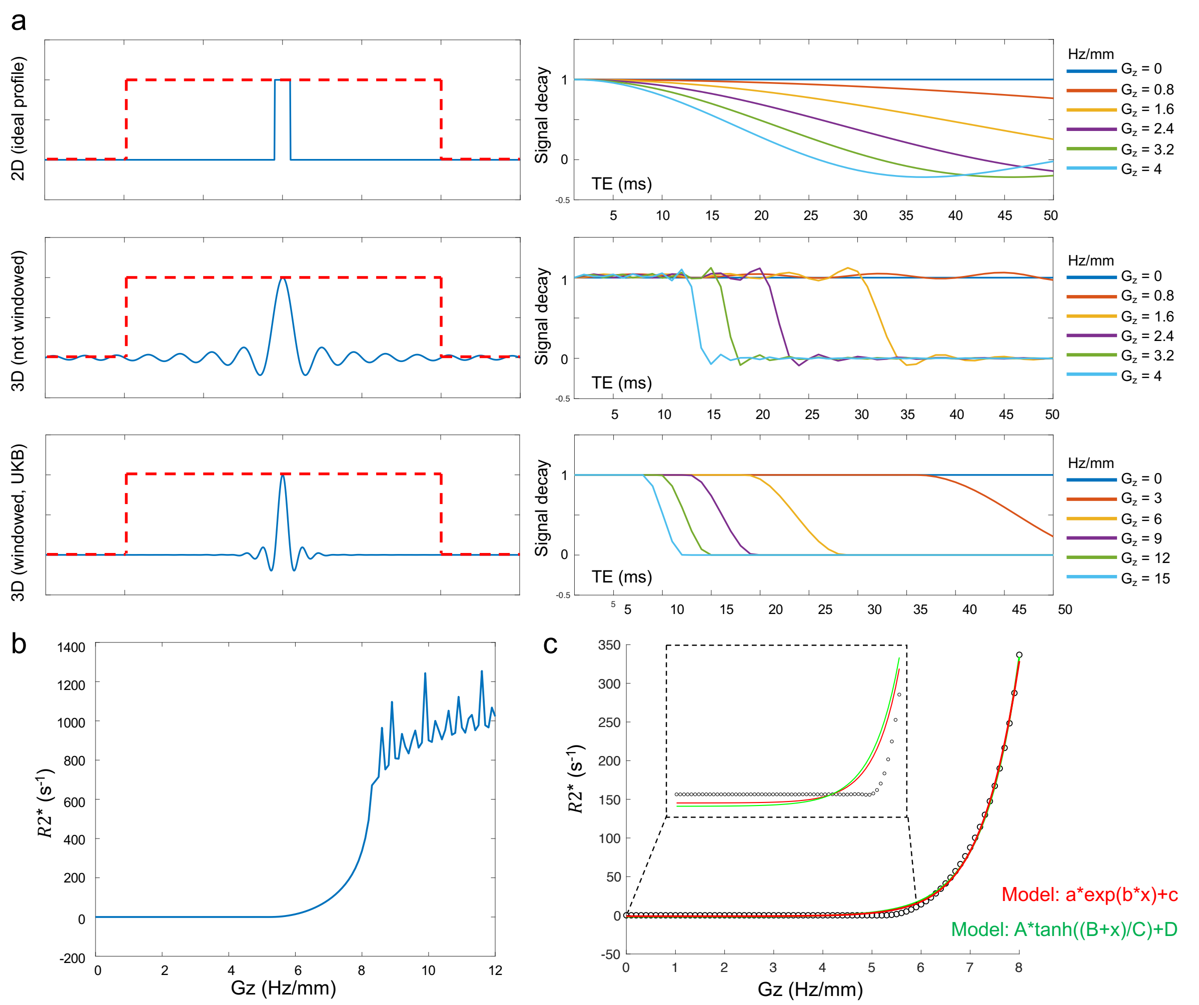

Supplementary Figure 14 (a) Simulated signal modulation in the presence of a linearly varying macroscopic field gradient (Gz), based on the procedure proposed by Hernando et al.[20]. On the left, the signal amplitude is indicated as a boxcar function (red line, representing a large homogeneous object), and the SRF (blue line) is displayed for 3 different imaging scenarios: 2D (10 mm slice – first row), 3D (10 mm slice – second row), and the UK Biobank swMRI protocol (3D, 3mm slice with k-space windowing – third row). One the right, the corresponding signal modulation is displayed as a function of TE and Gz. The presence of macroscopic field gradients (Gz > 0) leads to faster signal decay. **(b)** Simulated R2* estimates based on the UK Biobank protocol, as a function of Gz. The R2* estimate is dominated by noise when Gz > 8 Hz/mm, arising due to the negligible signal available for the second UK Biobank swMRI echo (20ms). **(c)** Two models were used to fit to the R2*-Gz curve over the range [0 8] Hz/m: *a*exp(b*x)+c* and *A*tanh((B+x)/C)+D*. Both models yielded similar fitted curves with $R^2 > 0.99$. Here, a = 0.0052, b = 1.384, c = -1.6924; A= 922.45, B = -9.0029, C = 1.3161, D = 921.23.

Experimental macroscopic field gradient deconfounding:

To reduce the confounding effect of field gradients on the association analyses with T2* IDPs, we generated macroscopic field gradient maps along the slice-direction (**Supplementary Fig. 15**) for all UK Biobank subjects. Median gradient magnitude values were subsequently calculated in each subcortical ROI as a summary measure of the background field gradient, used to develop an additional set of confounds when performing our association analysis. The distribution of median gradient magnitude (Gmag) for subcortical ROIs is shown in **Supplementary Fig. 16.**

To generate the macroscopic field gradient maps, the two-echo coil-combined phase data from each subject was unwrapped using PRELUDE and averaged to generate $\gamma\Delta B_{averaged}$. PRELUDE was chosen as it does not remove any background field components (which could bias field gradient estimates). $\gamma\Delta B_{averaged}$ was subsequently filtered using V-SHARP, $\gamma\Delta B_{V\_SHARP}$, with the macroscopic field of each subject estimated as:

$$\gamma\Delta B_{background} = \gamma\Delta B_{averaged} - \gamma\Delta B_{V\_SHARP} \quad [8]$$

Gmag was subsequently generated by taking the gradient magnitude (along the z-direction) of $\gamma\Delta B_{background}$ (**Supplementary Fig. 15**).

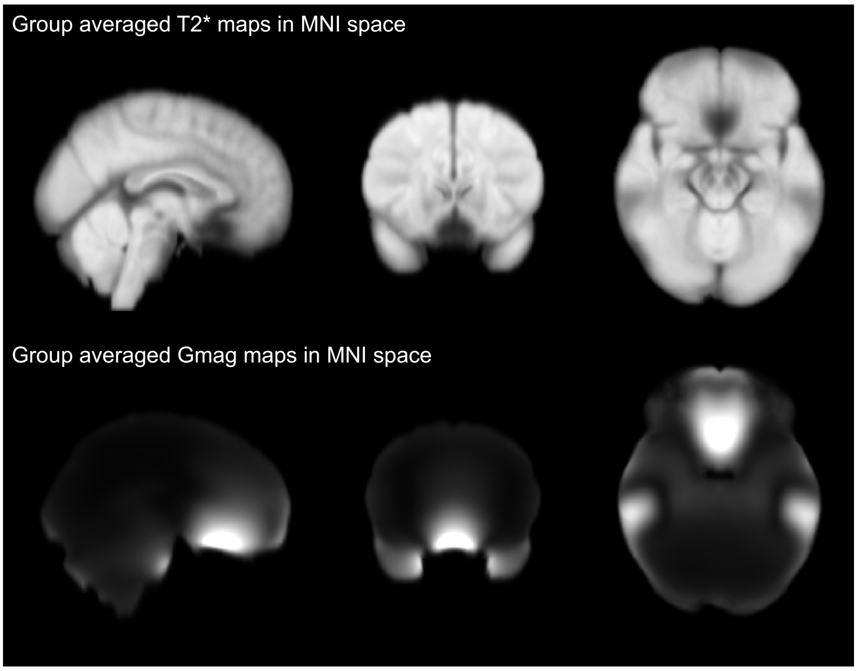

Supplementary Figure 15 Group average T2* map (top) and Gmag (bottom) over 200 randomly selected subjects. The sinus cavity and ear canals are the major sources of variations in background fields due to air/tissue interfaces.

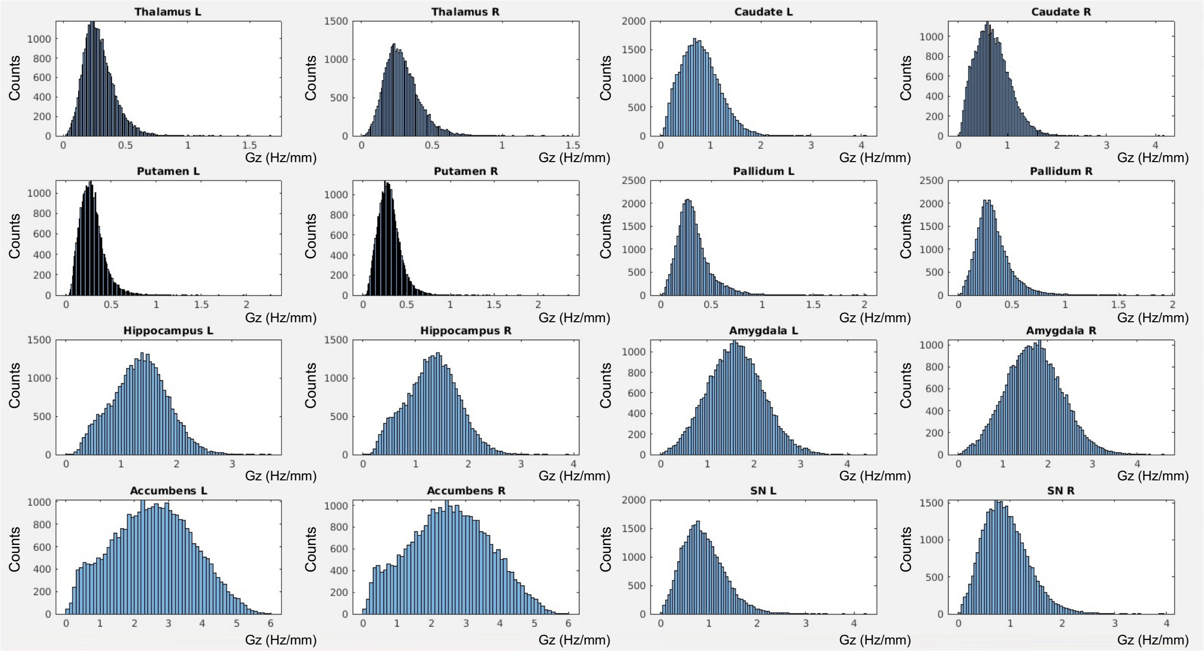

Supplementary Figure 16 Histograms of median Gmag in 16 ROIs across all UK Biobank subjects. Only the accumbens, amygdala and hippocampus regions contain a wide Gmag distribution exceeding 2 Hz/mm.

We subsequently modelled the voxelwise relationship between macroscopic field gradients (**Supplementary Fig. 15**) and R2* (1/T2*) (**Supplementary Fig. 17**) using data from 150 UK Biobank subjects with large variances in Gmag, to produce a set of confound to account for the background field gradient. To achieve this, we evaluated a series of different models (based on the simulations in **Supplementary Fig. 14c**) to identify the relationship between R2* and Gmag, as shown in **Supplementary Fig. 17**. The tanh model (*A*tanh((B+x)/C)+D*) model showed the best performance, defining *B*=-6.06 and *C*=1.87, similar to the fitting parameters estimated using the simulated data (**Supplementary Fig. 14c**, *B* = -9.00, *C* = 1.32). We used these fitting parameters establish a linear relationship between the R2* and median Gmag measures, setting $Gmag_{deconf} = tanh((-6.11+Gmag)/1.87)$.

Changes in the voxelwise R2* estimates manifest in regions with large macroscopic field gradients (**Supplementary Fig. 17**). From the median subcortical ROI analysis (**Supplementary Fig. 16**), only the accumbens, amygdala and hippocampus are confounded by large macroscopic field gradients (Gmag > 2 Hz/mm). Therefore, we only performed deconfounding (linear regression) for T2* IDPs in the accumbens, amygdala and hippocampus.

As shown in **Supplementary Fig. 18**, spurious associations between T2* accumbens IDPs and sinus/nasal-related phenotypes (including ICD10 codes related to *Nasal polyp* and *Chronic sinusitis*) are dramatically reduced after deconfounding for macroscopic field gradients.

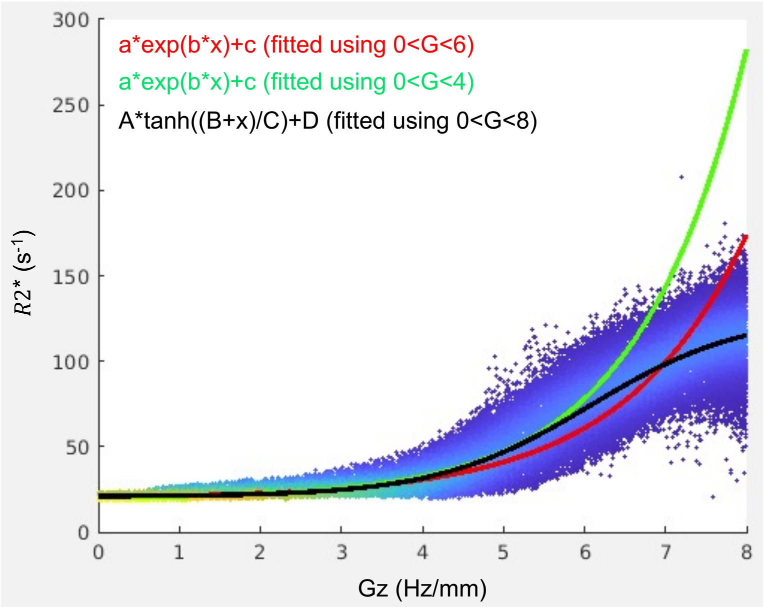

Supplementary Figure 17 Relationship between R2* and Gmag using voxel-wise data from selected 150 UK Biobank subjects with large variances in Gmag, fitting with an exponential and tanh model. The data scatter displays median R2* measures averaged across Gmag bins, with the colour representing the scatter density (blue – low density, green – high density). As the majority of median Gmag estimates were in the range 0<Gmag<4 Hz/mm, the exponential model *(a*exp(b*x)+c)* was fit to data in the range of 0<G<4 Hz/mm (green line) and of 0<G<6 Hz/mm (red line). The tanh function *(A*tanh((B+x)/C)+D)* (black line) showed the best performance across the whole range, particularly when Gmag > 6 Hz/mm.

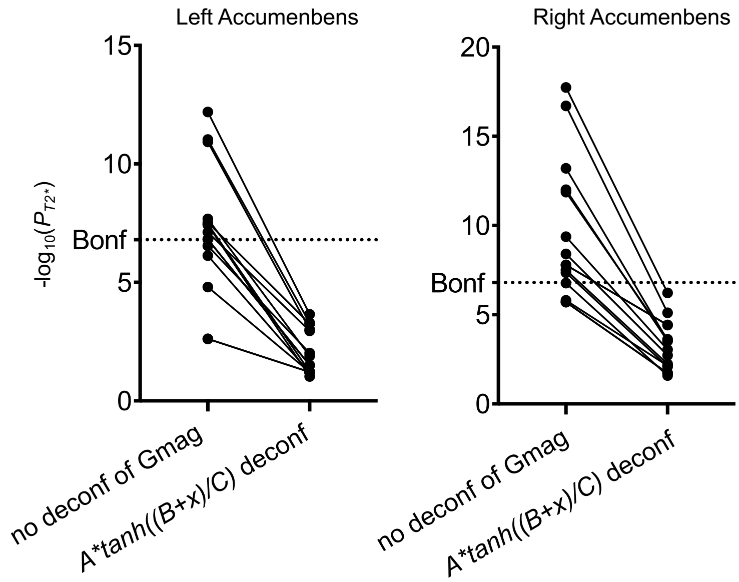

Supplementary Figure 18 Unadjusted -log$_{10}P$ values calculated in univariate (two-sided) cross-subject association between T2* accumbens IDPs and sinus/nasal-related phenotypes using n=35,273 subjects. Dashed horizontal line represents Bonferroni-corrected significance threshold. Here, each point represents one association with one unique phenotype. These associations are considered spurious as accumbens regions are in the vicinity of the sinus and no previous literature has linked T2* in accumbens with sinus conditions. Deconfounding using the "tanh" model was able to dramatically reduce these associations to non-significant.

No association was found between the macroscopic field gradients and QSM data (**Supplementary Fig. 19**), and thus this particular confound was only applied to T2* data.

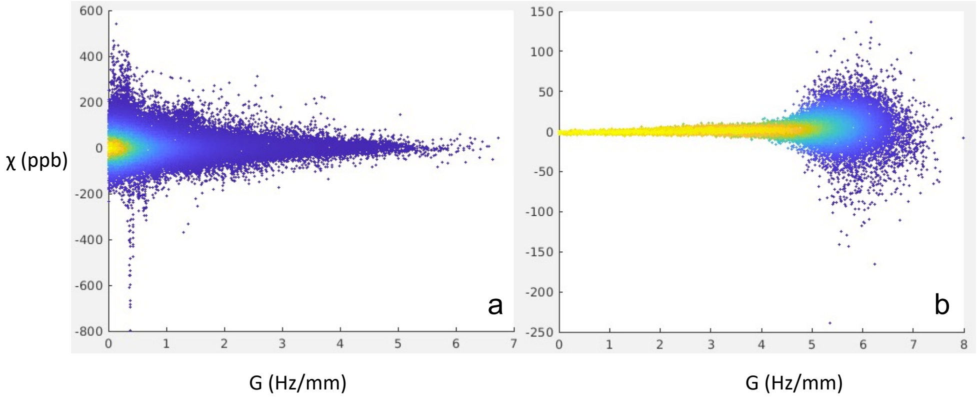

Supplementary Figure 19 Relationship between χ measures and macroscopic field gradient Gmag. **(a)** χ vs Gmag in voxel-wise data from the same set of 150 subjects as used for R2* vs Gmag in **Supplementary Fig. 17** and **(b)** median value in each bin of Gmag in **(a)**.

Section 3: comparisons of phenotypic association between QSM and T2* IDPs

Supplementary Figures 20-25 are comparisons of univariate (two-sided) phenotypic associations with QSM and T2* subcortical IDPs (ROI/phenotype pair shown if unadjusted P_{QSM} or P_{T2*} passed the Bonferroni-corrected threshold) using n=35,273 subjects.

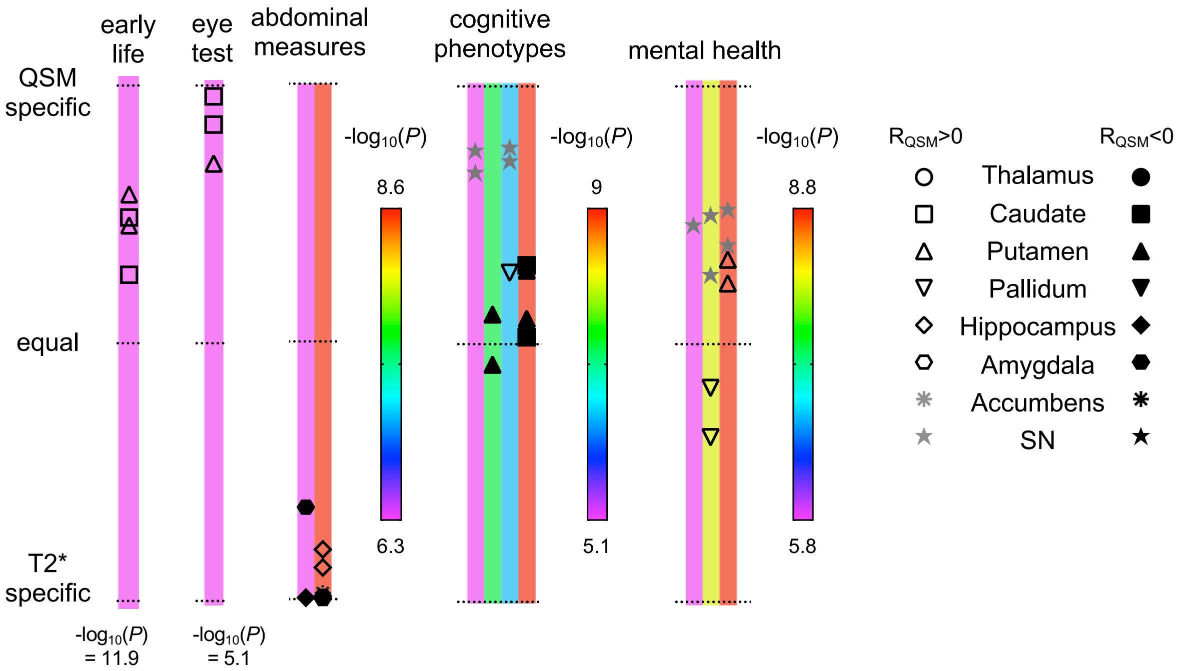

Supplementary Figure 20 Transformed Bland-Altman plot for the 5 categories that showed the smallest number of associations (early life factors, eye test, abdominal measures, cognitive phenotypes and mental health).

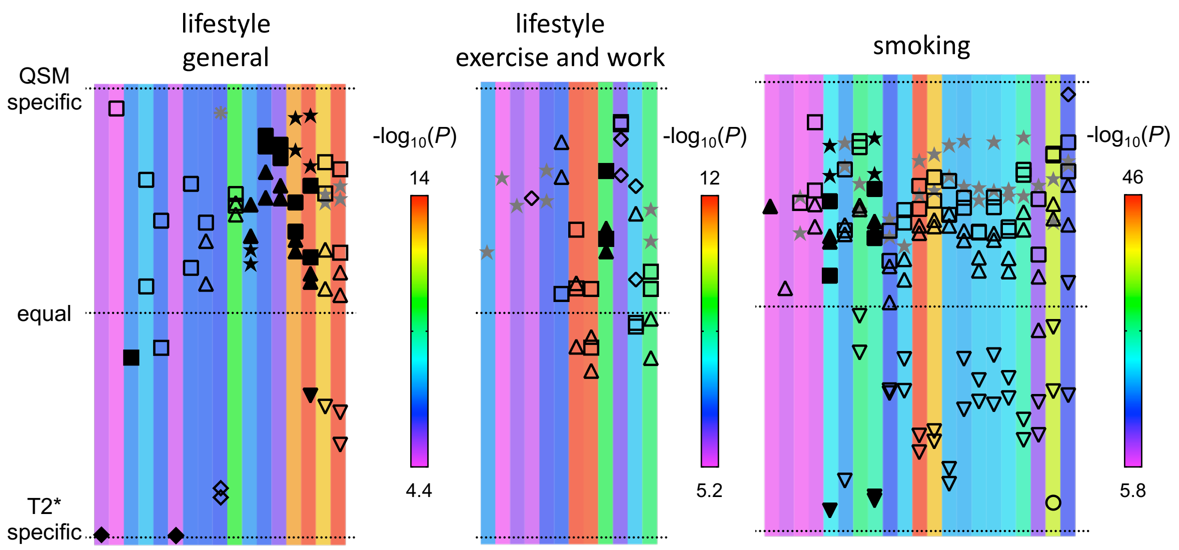

Supplementary Figure 21 Transformed Bland-Altman plot for lifestyle general, lifestyle exercise and work, smoking categories.

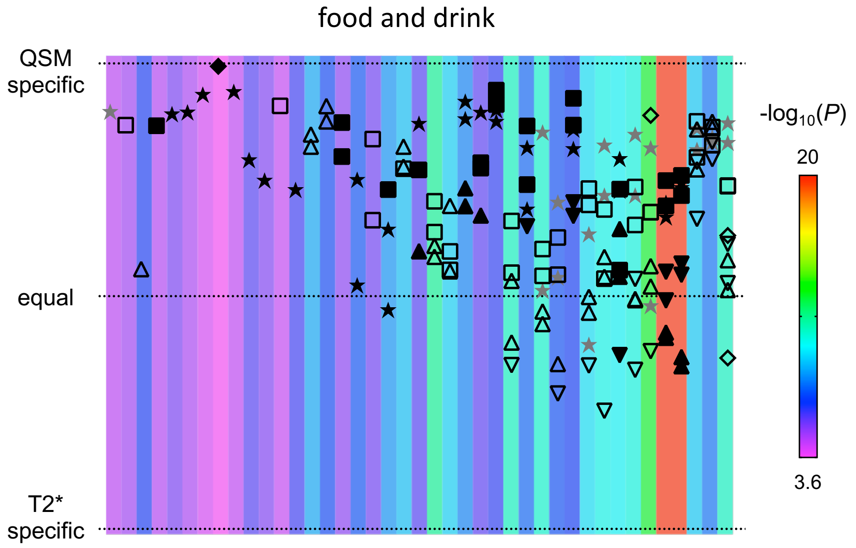

Supplementary Figure 22 Transformed Bland-Altman plot for the food and drink category.

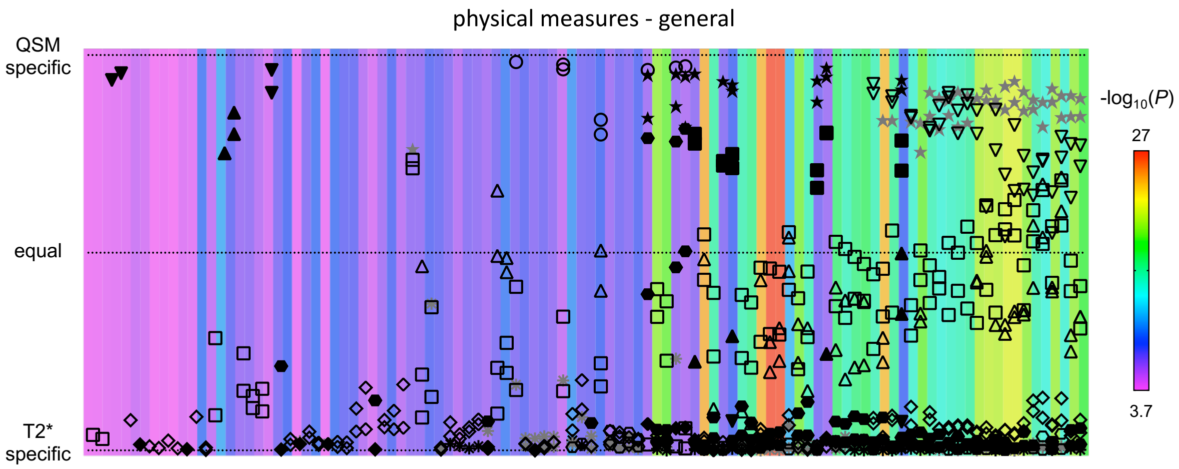

Supplementary Figure 23 Transformed Bland-Altman plot for the physical measures (general) category.

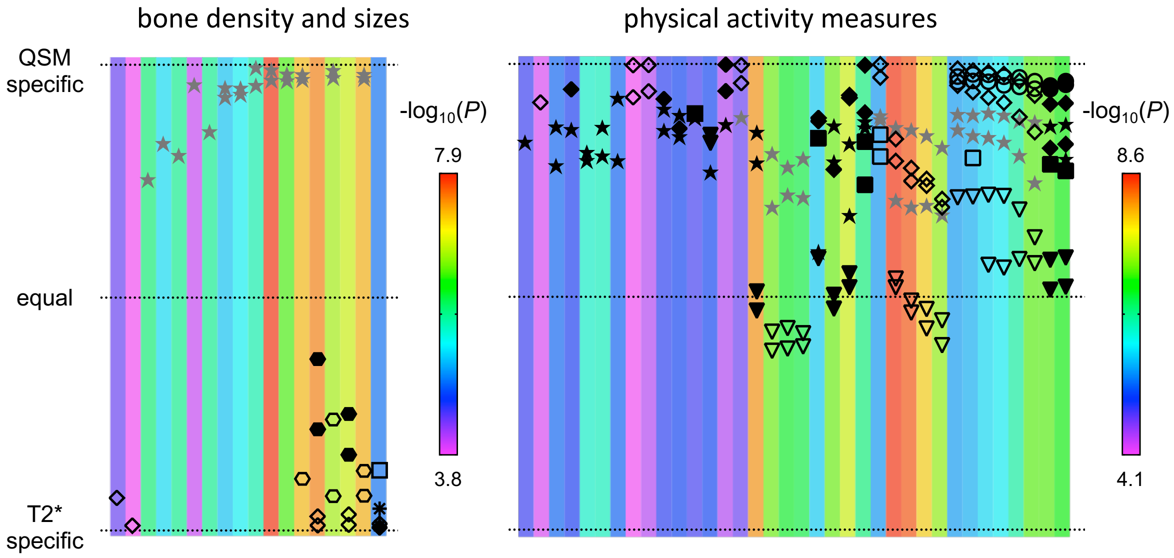

Supplementary Figure 24 Transformed Bland-Altman plot for bone density and physical activity categories.

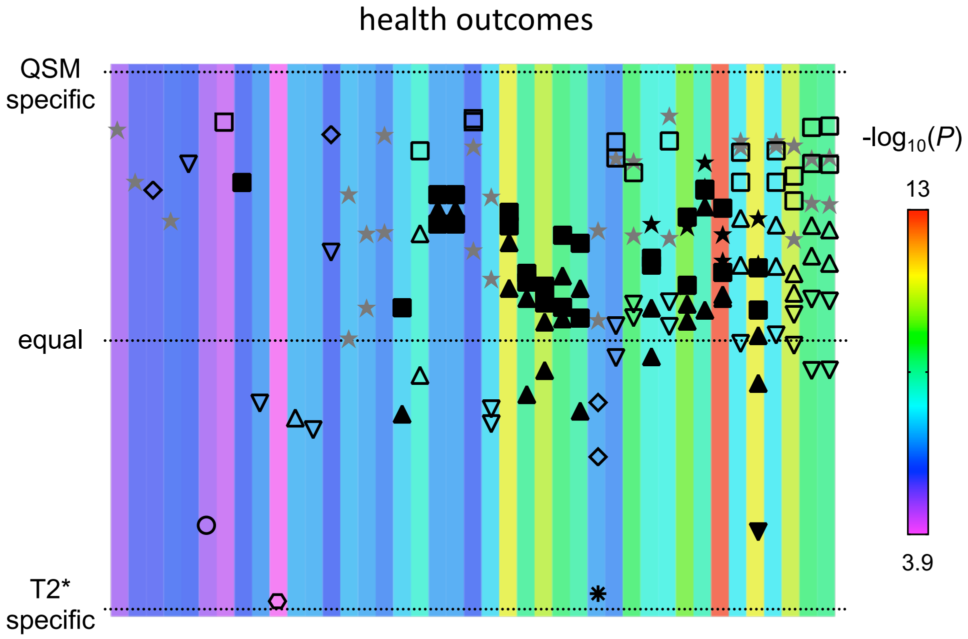

Supplementary Figure 25 Transformed Bland-Altman plot for the health outcomes category.

Section 4: χ voxel-wise association maps for example associations in each phenotype category

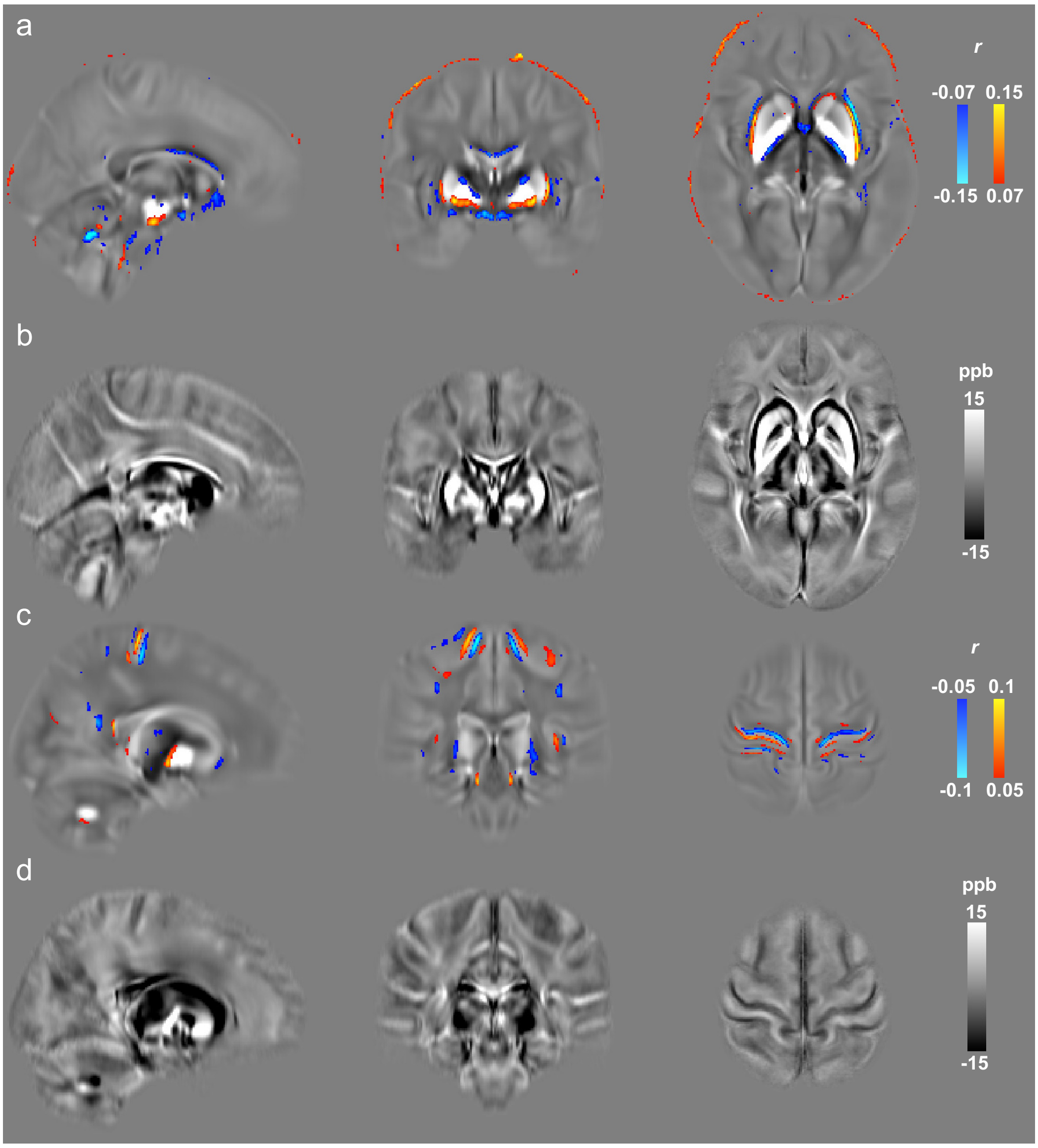

Supplementary Figure 26 (a) Voxel-wise correlation map for *Total BMD*, with *r* values overlaid onto the susceptibility atlas. **(b)** Susceptibility aging atlas displaying the same slice as (a), generated by taking the difference between χ maps from the youngest (< 52yo) and oldest (> 75yo) age groups in UK Biobank (each group had around 2000 subjects). Here, differences due to aging are largely driven by atrophy, displaying similar contrast to the regions of high *r* values in (a). As brains were skull-stripped in this study, clusters of associated voxels at the brain boundary might arise due to atrophy of the whole brain. Note that (b) and (d) do not show χ maps themselves, but rather the spatial differences in χ between two age groups. For example, the optic radiation appears bright in (b), indicating changes in χ (possibly due to atrophy) in this white matter fibre region. **(c)** Voxel-wise correlations maps of *BMI* also demonstrate similar contrast to regions of brain atrophy, with **(d)** displaying the aging atlas in the same slices as (c).

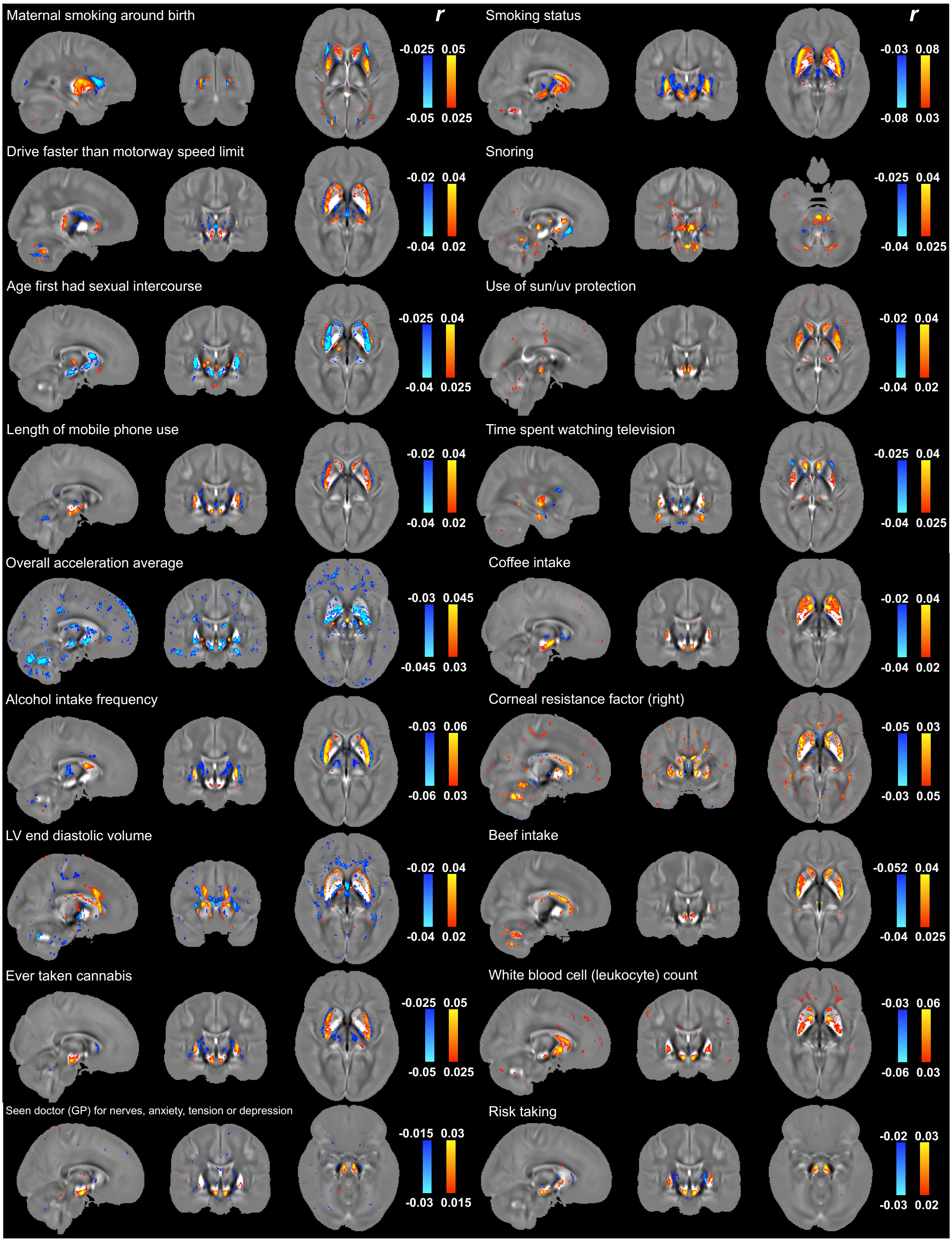

Supplementary Figure 27 Voxel-wise association maps of 6 example phenotypes with χ maps (aligned in MNI space) from 35,273 subjects. Pearson correlation r is shown as color overlay (red-yellow for positive r and blue for negative r) on the population-average χ map.

Section 5: Additional GWAS results for QSM and T2* IDPs

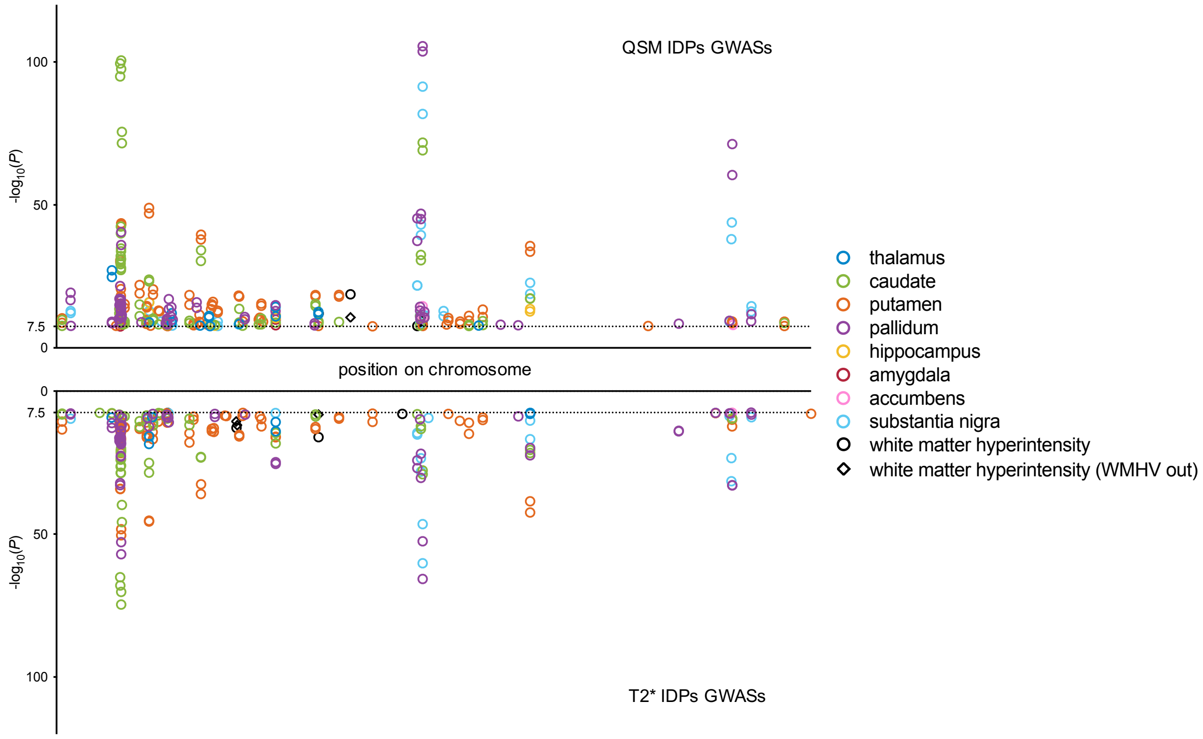

Supplementary Figure 28 Comparison of all lead genetic associations identified in GWASs of every QSM/T2* IDP (showing two-sided, unadjusted -$\log_{10}P$ values for the discovery cohort n=19,720). Each circle represents an association between a IDP and a lead genetic variant where x-axis showing variant's position on chromosome and y-axis gives its -$\log_{10}P$ in discovery cohort. QSM IDPs generally showed more genetic associations and higher -$\log_{10}P$ values compared to T2* IDPs.

Manhattan plots (showing two-sided, unadjusted -log10P values for the discovery cohort n=19,720) of every GWAS performed for QSM and T2* IDPs are shown below.

QSM left thalamus

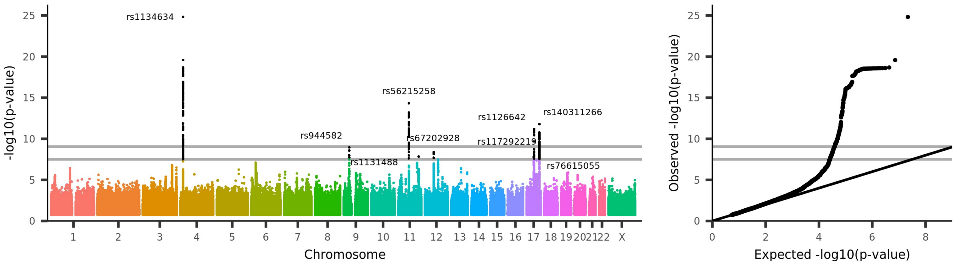

QSM right thalamus

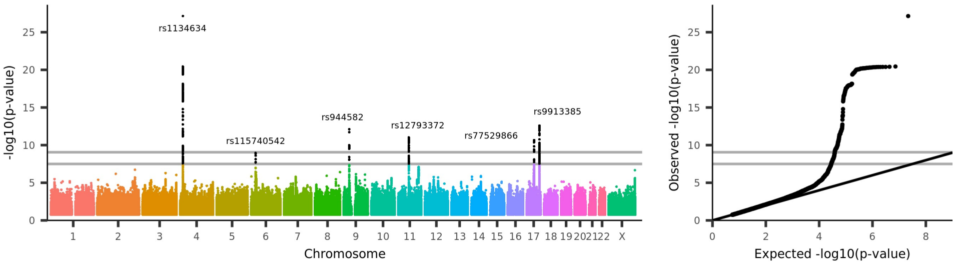

QSM left caudate

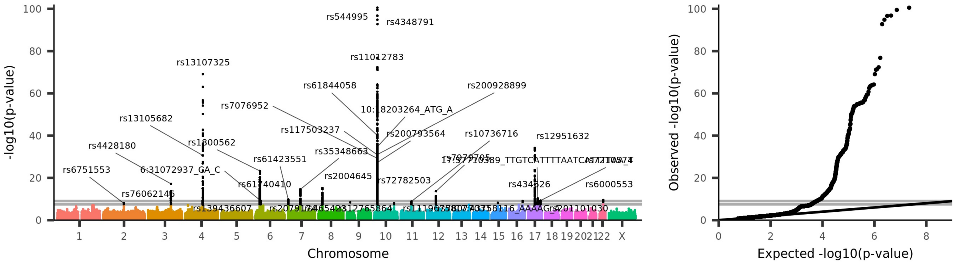

QSM right caudate

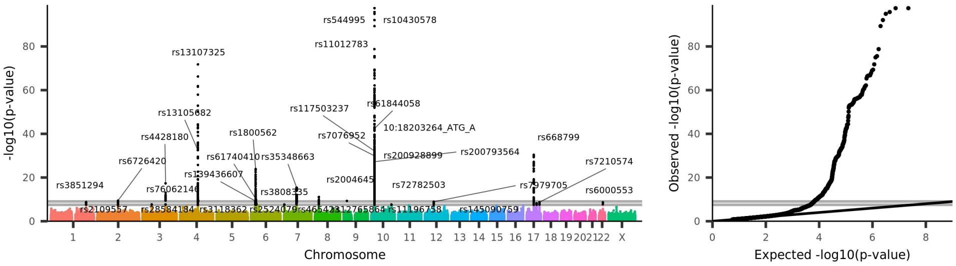

QSM left putamen

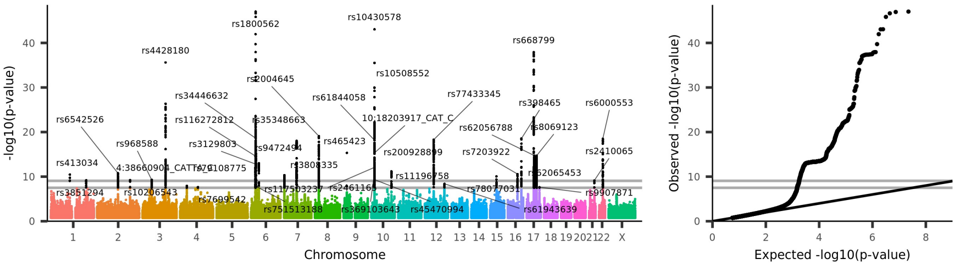

QSM right putamen

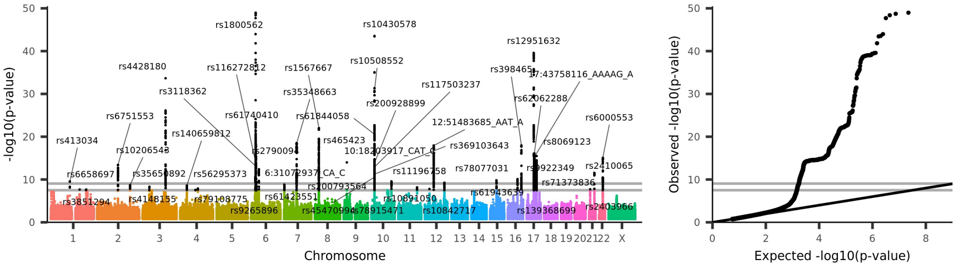

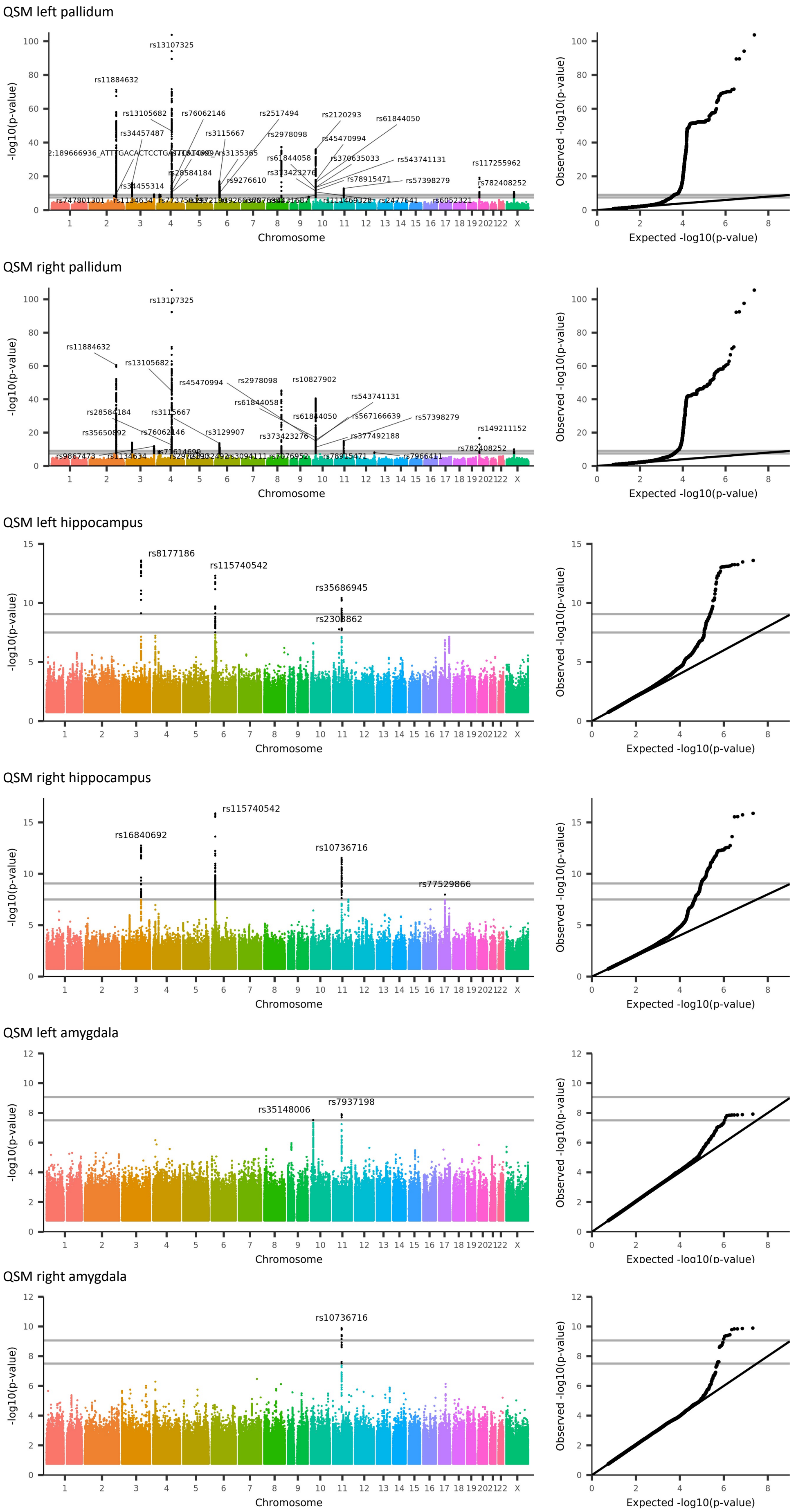
QSM left pallidum
rs13107325
rs11884632
rs13105682
rs76062146
rs2517494
rs2120293
rs61844050
rs34457487
rs3115667
rs2978098
rs45470994
rs3135365
rs61844058
rs370635033
rs543741131
rs117255962
rs28584184
rs373423276
rs9276610
rs78915471
rs57398279
rs782408252
rs34455314
rs747801301
rs1134634
rs111469328
rs2477641
rs6052321
QSM right pallidum
rs13107325
rs11884632
rs13105682
rs45470994
rs2978098
rs10827902
rs543741131
rs61844058
rs28584184
rs3115667
rs61844050
rs567166639
rs57398279
rs35650892
rs76062146
rs3129907
rs373423276
rs377492188
rs149211152
rs782408252
rs9867473
rs1134634
rs3094111
rs7076952
rs78915471
rs7966411
QSM left hippocampus
rs8177186
rs115740542
rs35686945
QSM right hippocampus
rs115740542
rs16840692
rs10736716
rs77529866
QSM left amygdala
rs35148006
rs7937198
QSM right amygdala
rs10736716
-log10(p-value)
Chromosome
Observed -log10(p-value)
Expected -log10(p-value)

QSM left accumbens

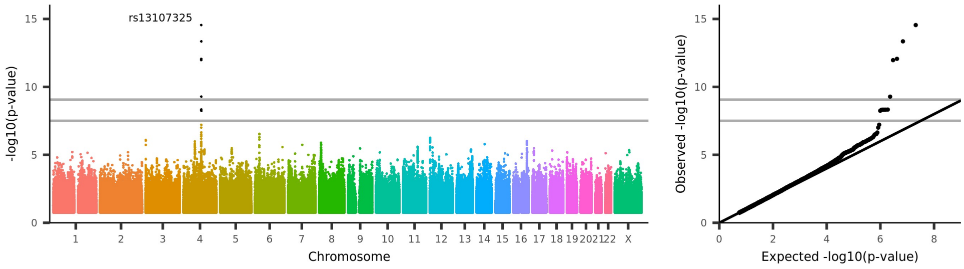

QSM right accumbens

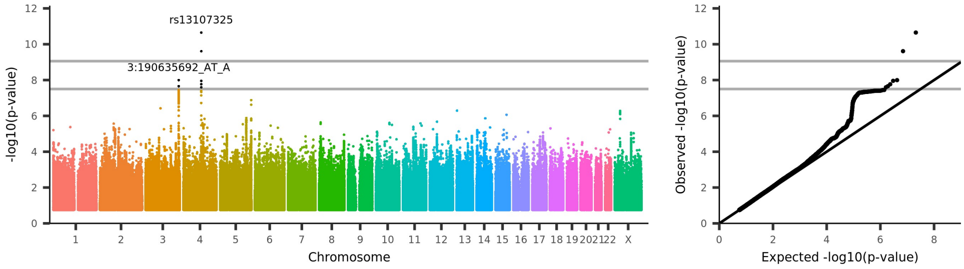

QSM left SN

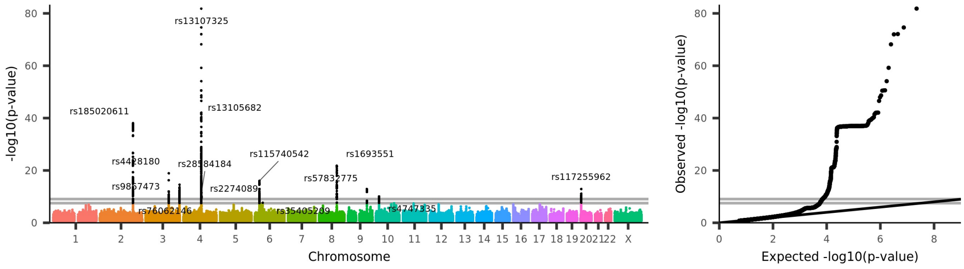

QSM right SN

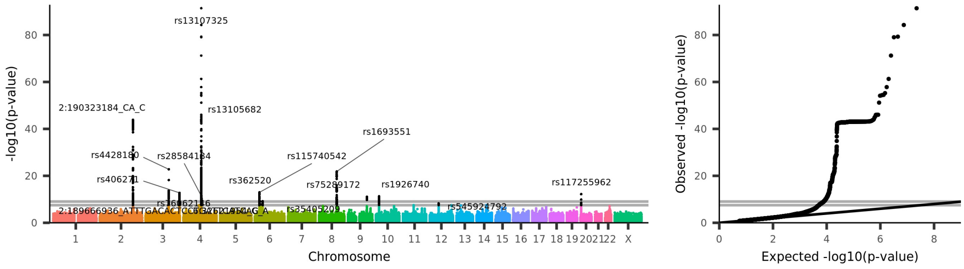

QSM WMH (without regressing out WMH volume)

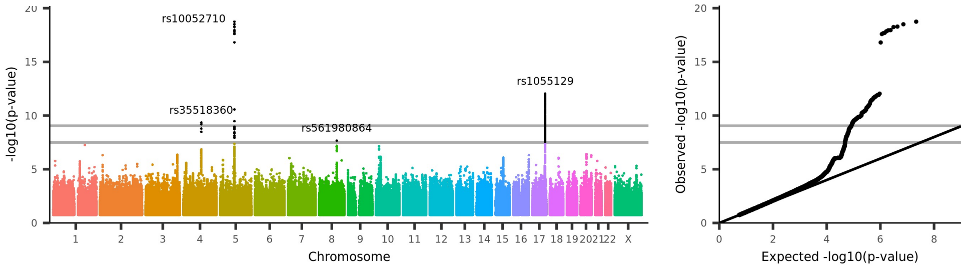

QSM WMH (after regressing out WMH volume)

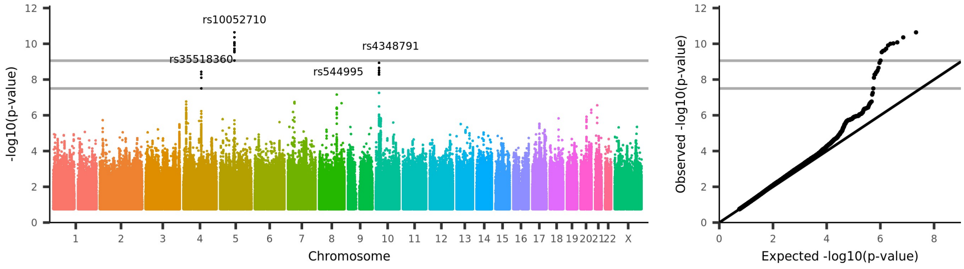

T2* left thalamus

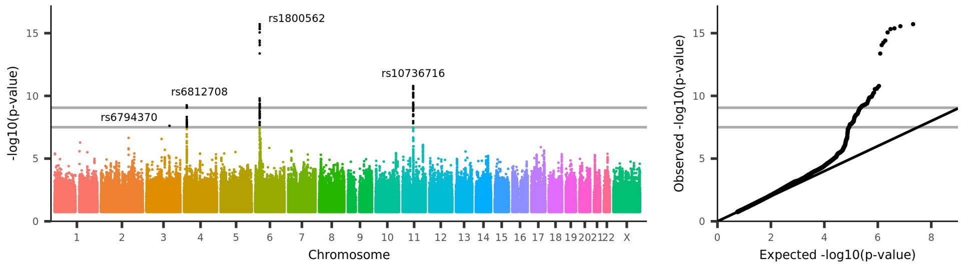

T2* right thalamus

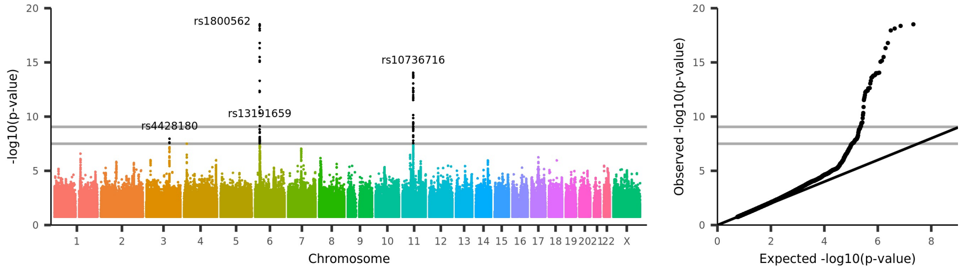

T2* left caudate

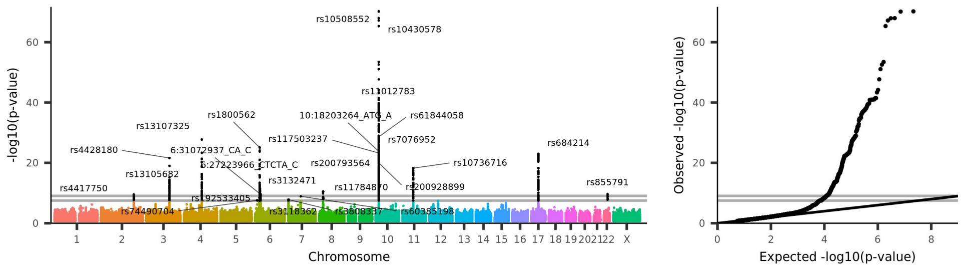

T2* right caudate

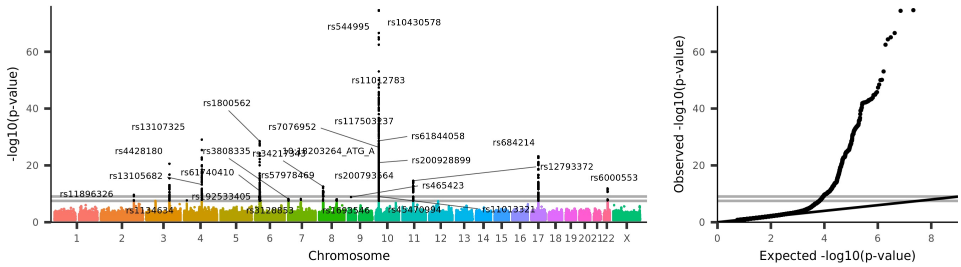

T2* left putamen

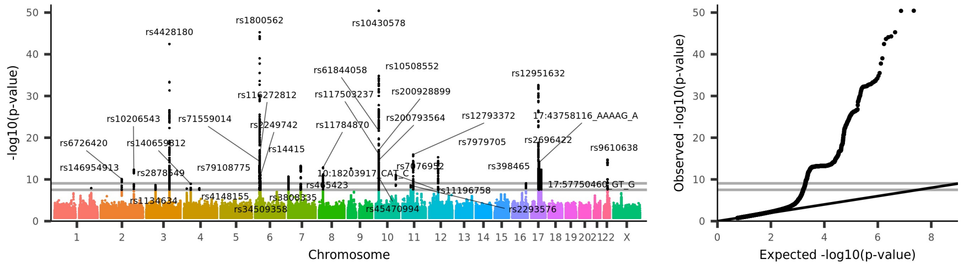

T2* right putamen

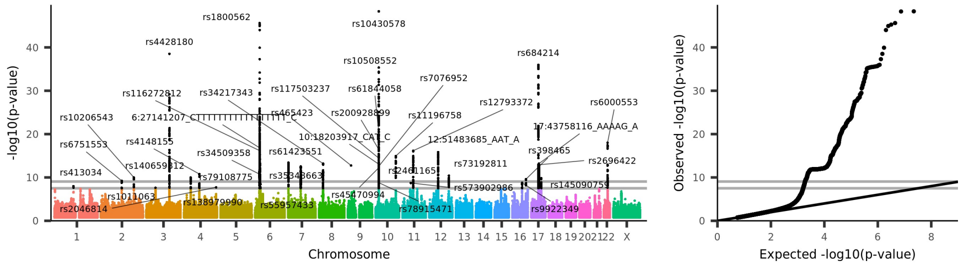

T2* left pallidum

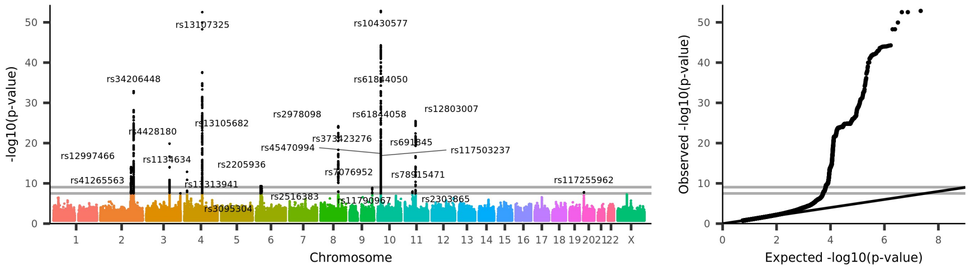

T2* right pallidum

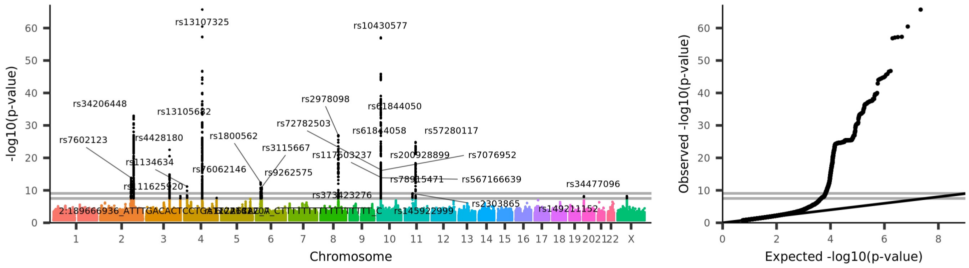

T2* left hippocampus

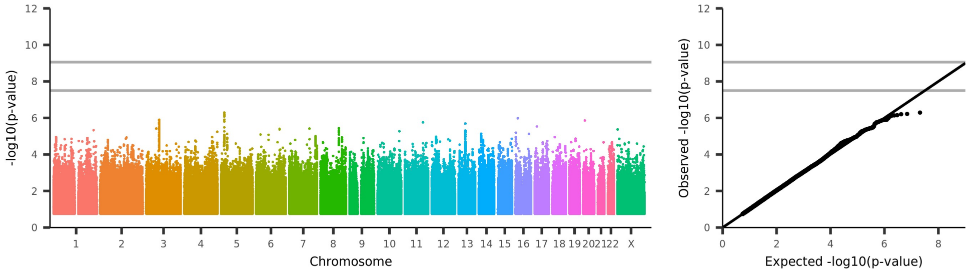

T2* right hippocampus

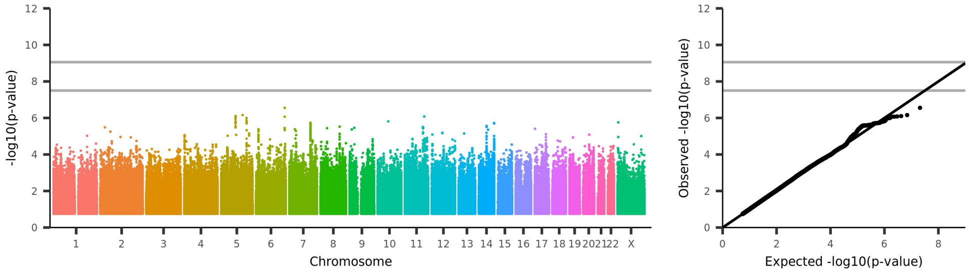

T2* left amygdala

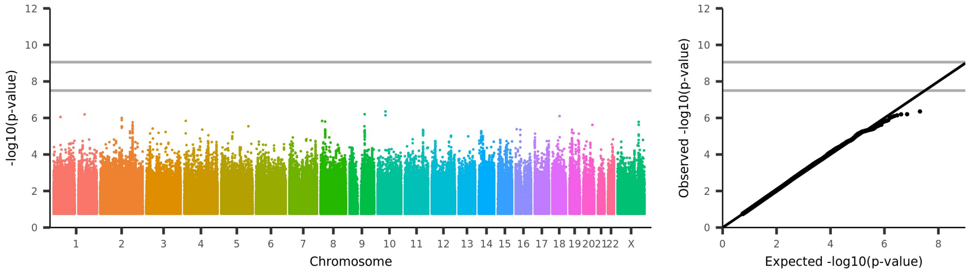

T2* right amygdala

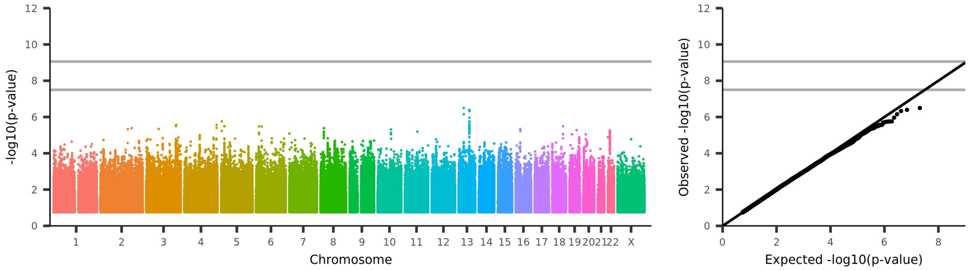

T2* left accumbens

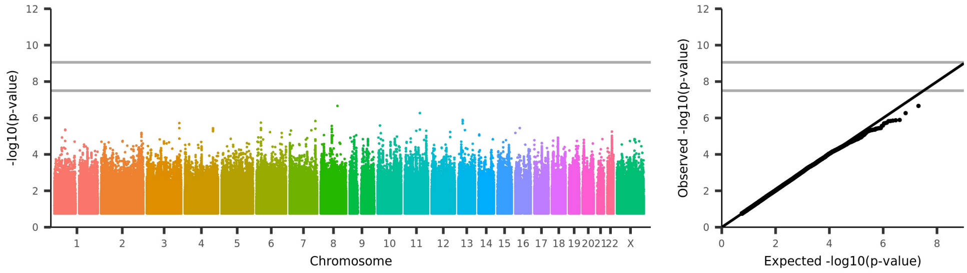

T2* right accumbens

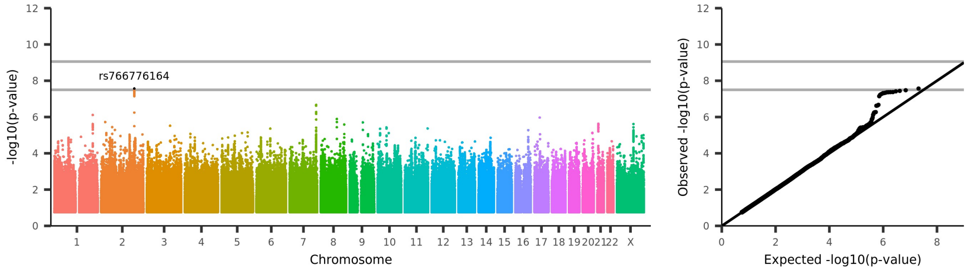

T2* left SN

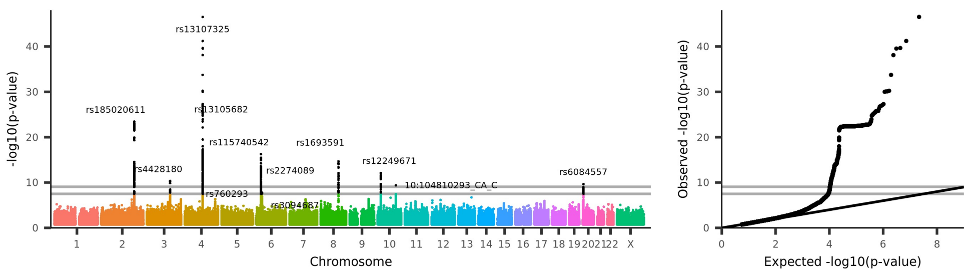

T2* right SN

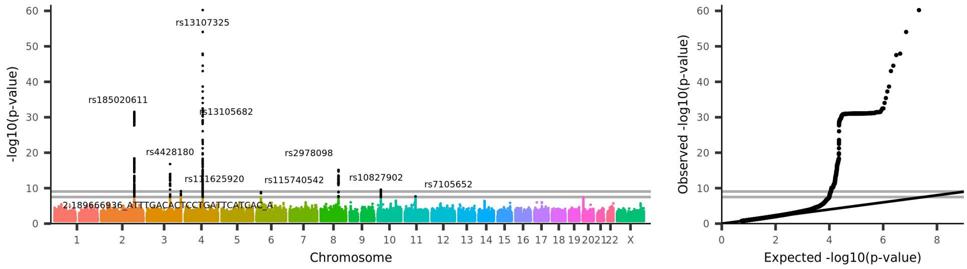

T2* WMH (without regressing out WMH volume)

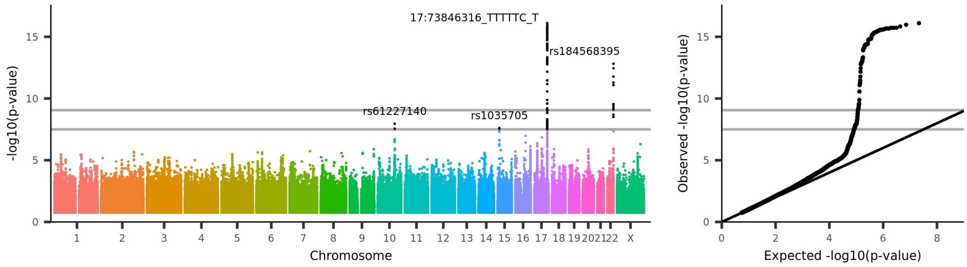

T2* WMH (after regressing out WMH volume)

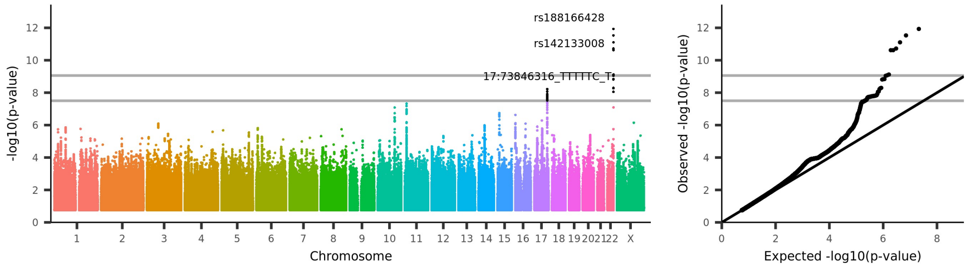

Section 6: Associations between χ or T2* and genetic variants not directly related to myelin, iron and calcium homeostasis

Associations between QSM and variants in genes related to myelin, iron and calcium were expected, since they are all known to affect brain tissue constituents[21]. However, many of the associations identified in this study could not be directly related to none of these pathways. A few notable examples were associations with genes encoding extracellular matrix proteins, transcription factors and proteins related to immune response.

We observed associations with two genes related to the extracellular matrix, *COL3A1* and *VCAN*. Both QSM and T2* in the globus pallidum and substantia nigra were associated with variants related to the *COL3A1* gene (cluster 9, peak variant 2:189666936_ATTTGACACTCCTGATTCATCAC_A, P=3.07*E-10). COL3A1 encodes the type III collagen, a fibrillar-forming collagen that is a major component of the extracellular matrix in a variety of organs in adults. It's also expressed throughout embryogenesis, and is considered to play a central role in cerebral cortex development. Patients with mutations in COL3A1 have a variety of connective tissue anomalies and can also present profound brain anomalies in both grey and white matter[22].

T2* in the globus palidus and QSM in the hippocampus were also associated with variants in the *CD82* gene, including a synonymous exonic variant (cluster 62, rs2303865, P=9.42*E-10). *CD82* encodes a membrane glycoprotein highly expressed in myelinating oligodendrocytes with multiple roles, such as metastasis suppression, immune response, and in the development of oligodendrocytes and maturation of oligodendrocyte precursors. It may regulate myelin proteins gene transcription or stabilize protein levels[23,24]. This same variant has been previously associated to white matter microstructure measurements [25]. Interestingly, in the projected maps, genetic variants related to both *GFAP* and *CD82* were associated with QSM in most of the white matter tracts.

Surprisingly, T2* and QSM IDPs were associated to many genetic variants related to transcription factors. Transcription factors regulate the transcription of DNA to RNA and can modulate the expression of multiple genes. It is challenging to anticipate how variants in transcription factors would lead to changes in T2* or QSM in the brain. As an example, QSM in the putamen and in substantia nigra were associated with a variant in the *RUNX2* gene (cluster 33, rs9472494, P=1.02*E-13). This gene encodes a transcription factor that plays a major role in osteoblastic differentiation and skeletal morphogenesis, but that is expressed in many tissues including the brain[26]. In the nervous system, RUNX2 acts on its development, regeneration and repair, regulating multiple mechanisms in neurons and glia[27,28], but it can also promote ectopic vascular biomineralization[29]. Down-regulation of RUNX2 signalling has been reported in the dorsolateral prefrontal cortex of patients with schizophrenia[30], as well as reduced expression of *RUNX2* in the hippocampus of patients with bipolar disorder[31]. In our study, the association between QSM and variants in *RUNX2* could be a consequence of alterations in multiple pathways, such as an increase in vascular calcifications or differences in brain development leading to changes in tissue microstructure.

Finally, a high number of associations, including associations with very low p-values, were related to the gene *MRC1* (or *MRC1L1*), with seven clusters and 78 associations being related to this gene (T2* and QSM in the globus pallidum, caudate, putamen, in addition to QSM in the amygdala and WMH; strongest associations in cluster 48, rs544995, P=3.18*E-100). *MRC1* encodes a molecular scavenger protein, also known as CD206, that mediates the endocytosis

of glycoproteins and is primarily related to immune response. It acts by clearing harmful glycoconjugates, enzymes, hormones, cell membranes, extracellular matrix components, and micro-organisms through recognition of their carbohydrate structures[32,33]. It mediates many roles, including clearance of inflammatory molecules[33], remodelling of the extracellular matrix, immune response and cavity and scar formation after injury in the CNS[34], and is known to be expressed by astrocytes and microglia in the brain[35]. In autism spectrum disorder, *MRC1* is overexpressed in the white matter[36], and the variant rs544995 has been related to sarcoidosis, a chronic inflammatory disease[37].

How variants in these genes could affect tissue magnetic susceptibility and T2* is not clear. Indirect effects via regulation of iron or calcium homeostasis, brain development, plasticity and myelination, immune response, regulation of cell cycle, and scavenging of harmful molecules, could result in the accumulation of iron and/or calcium or in differences in the brain microstructure, for example.

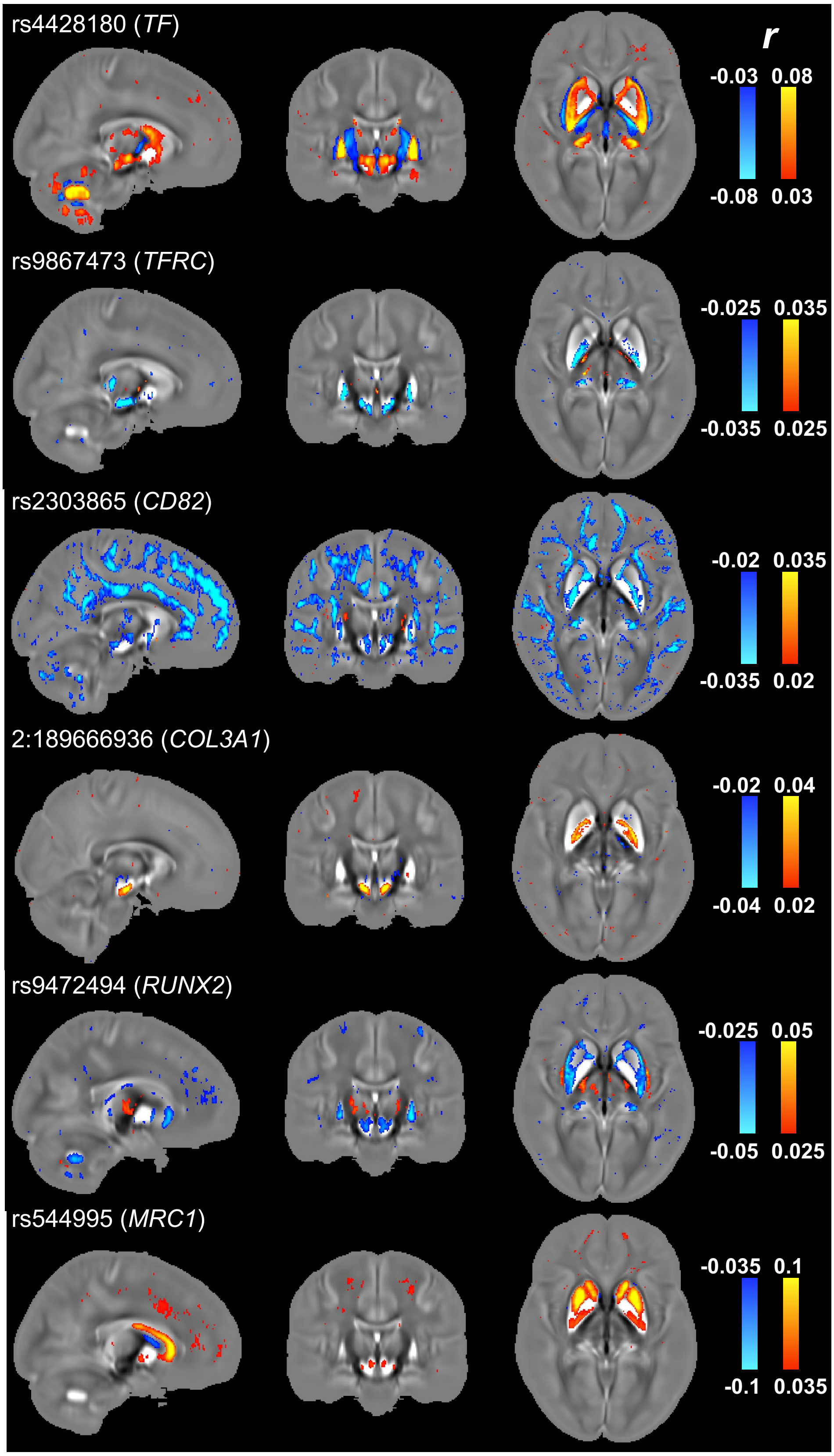

Supplementary Figure 29 Voxel-wise correlation maps of 6 top genetic variants using n=35,273 subjects, with *r* values overlaid onto the susceptibility atlas.

Reference

1. Eckstein, K. *et al.* Computationally Efficient Combination of Multi-channel Phase Data From Multi-echo Acquisitions (ASPIRE). *Magnetic Resonance in Medicine* **79**, 2996–3006 (2018).
2. Jenkinson, M. Fast, automated, N-dimensional phase-unwrapping algorithm. *Magnetic Resonance in Medicine* **49**, 193–197 (2003).
3. Schofield, M. A. & Zhu, Y. Fast phase unwrapping algorithm for interferometric applications. *Opt. Lett.* **28**, 1194–1196 (2003).
4. Wu, B., Li, W., Avram, A. V., Gho, S. M. & Liu, C. Fast and tissue-optimized mapping of magnetic susceptibility and T2* with multi-echo and multi-shot spirals. *NeuroImage* **59**, 297–305 (2012).
5. Lindemeyer, J., Worthoff, W. A., Shymanskaya, A. & Shah, N. J. Iterative Restoration of the Fringe Phase (REFRASE) for QSM. *Frontiers in Neuroscience* **15**, 474 (2021).
6. Schweser, F., Deistung, A., Lehr, B. W. & Reichenbach, J. R. Quantitative imaging of intrinsic magnetic tissue properties using MRI signal phase: An approach to in vivo brain iron metabolism? *NeuroImage* **54**, 2789–2807 (2011).
7. Liu, T. *et al.* A novel background field removal method for MRI using projection onto dipole fields (PDF). *NMR in Biomedicine* **24**, 1129–1136 (2011).
8. Li, W., Wu, B. & Liu, C. iHARPERELLA: an improved method for integrated 3D phase unwrapping and background phase removal. in *Proc Intl Soc Mag Reson Med, p3313* (2015).
9. Sun, H. & Wilman, A. H. Background field removal using spherical mean value filtering and Tikhonov regularization. *Magnetic Resonance in Medicine* **71**, 1151–1157 (2014).
10. Zhou, D., Liu, T., Spincemaille, P. & Wang, Y. Background field removal by solving the Laplacian boundary value problem. *NMR in Biomedicine* **27**, 312–319 (2014).
11. Li, W. *et al.* A method for estimating and removing streaking artifacts in quantitative susceptibility mapping. *NeuroImage* **108**, 111–122 (2015).
12. Wei, H. *et al.* Streaking artifact reduction for quantitative susceptibility mapping of sources with large dynamic range. *NMR in Biomedicine* **28**, 1294–1303 (2015).
13. Liu, J. *et al.* Morphology enabled dipole inversion for quantitative susceptibility mapping using structural consistency between the magnitude image and the susceptibility map. *NeuroImage* **59**, 2560–2568 (2012).
14. Zhang, L. *et al.* Fast quantitative susceptibility reconstruction via total field inversion with improved weighted L0 norm approximation. *NMR in Biomedicine* **32**, e4067 (2019).
15. Wang, Y. & Liu, T. Quantitative susceptibility mapping (QSM): Decoding MRI data for a tissue magnetic biomarker. *Magnetic Resonance in Medicine* **73**, 82–101 (2015).
16. Griffanti, L. *et al.* BIANCA (Brain Intensity AbNormality Classification Algorithm): A new tool for automated segmentation of white matter hyperintensities. *NeuroImage* **141**, 191–205 (2016).
17. de Groot, M. *et al.* Improving alignment in Tract-based spatial statistics: Evaluation and optimization of image registration. *NeuroImage* **76**, 400–411 (2013).
18. Llera, A., Vidaurre, D., Pruim, R. H. R. & Beckmann, C. F. Variational Mixture Models with Gamma or inverse-Gamma components. (2016).
19. Persson, N. *et al.* Age and sex related differences in subcortical brain iron concentrations among healthy adults. *NeuroImage* **122**, 385–398 (2015).

20. Hernando, D., Vigen, K. K., Shimakawa, A. & Reeder, S. B. R mapping in the presence of macroscopic B0 field variations. *Magnetic Resonance in Medicine* **68**, 830–840 (2012).
21. Liu, C., Li, W., Tong, K. A., Yeom, K. W. & Kuzminski, S. Susceptibility-weighted imaging and quantitative susceptibility mapping in the brain. *Journal of Magnetic Resonance Imaging* **42**, 23–41 (2015).
22. Horn, D. *et al.* Biallelic COL3A1 mutations result in a clinical spectrum of specific structural brain anomalies and connective tissue abnormalities. *American Journal of Medical Genetics Part A* **173**, 2534–2538 (2017).
23. Mela, A. & Goldman, J. E. CD82 Blocks cMet Activation and Overcomes Hepatocyte Growth Factor Effects on Oligodendrocyte Precursor Differentiation. *Journal of Neuroscience* **33**, 7952–7960 (2013).
24. Mela, A. & Goldman, J. E. The Tetraspanin KAI1/CD82 Is Expressed by Late-Lineage Oligodendrocyte Precursors and May Function to Restrict Precursor Migration and Promote Oligodendrocyte Differentiation and Myelination. *Journal of Neuroscience* **29**, 11172–11181 (2009).
25. Zhao, B. *et al.* Large-scale GWAS reveals genetic architecture of brain white matter microstructure and genetic overlap with cognitive and mental health traits (n = 17,706). *Molecular Psychiatry* (2019) doi:10.1038/s41380-019-0569-z.
26. Jeong, J. H. *et al.* Expression of runx2 transcription factor in non-skeletal tissues, sperm and brain. *Journal of Cellular Physiology* **217**, 511–517 (2008).
27. Wang, J. W. & Stifani, S. Roles of Runx Genes in Nervous System Development. in *RUNX Proteins in Development and Cancer* (eds. Groner, Y. et al.) 103–116 (Springer Singapore, 2017). doi:10.1007/978-981-10-3233-2_8.
28. Benítez-Burraco, A. & Boeckx, C. Possible functional links among brain- and skull-related genes selected in modern humans. *Frontiers in Psychology* **6**, 1–19 (2015).
29. Cobb, A. M. *et al.* Runx2 (Runt-Related Transcription Factor 2) Links the DNA Damage Response to Osteogenic Reprogramming and Apoptosis of Vascular Smooth Muscle Cells. *Arteriosclerosis, Thrombosis, and Vascular Biology* **41**, 1339–1357 (2021).
30. Karpiński, P., Samochowiec, J., Sąsiadek, M. M., Łaczmański, Ł. & Misiak, B. Analysis of global gene expression at seven brain regions of patients with schizophrenia. *Schizophrenia Research* **223**, 119–127 (2020).
31. Benes, F. M. *et al.* Regulation of the GABA cell phenotype in hippocampus of schizophrenics and bipolars. *Proceedings of the National Academy of Sciences of the United States of America* **104**, 10164–10169 (2007).
32. Napper, C. E., Drickamer, K. & Taylor, M. E. Collagen binding by the mannose receptor mediated through the fibronectin type II domain. *Biochemical Journal* **395**, 579–586 (2006).
33. Allavena, P., Chieppa, M., Monti, P. & Piemonti, L. From Pattern Recognition Receptor to Regulator of Homeostasis: The Double-Faced Macrophage Mannose Receptor. *Critical Reviews in Immunology* **24**, 179–192 (2004).
34. Fitch, M. T., Doller, C., Combs, C. K., Landreth, G. E. & Silver, J. Cellular and molecular mechanisms of glial scarring and progressive cavitation: in vivo and in vitro analysis of inflammation-induced secondary injury after CNS trauma. *The Journal of neuroscience : the official journal of the Society for Neuroscience* **19**, 8182–98 (1999).
35. Burudi, E. M. E., Riese, S., Stahl, P. D. & R□gnier-Vigouroux, A. Identification and functional characterization of the mannose receptor in astrocytes. *Glia* **25**, 44–55 (1999).

36. Sciara, A. N. *et al.* Neuroinflammatory Gene Expression Alterations in Anterior Cingulate Cortical White and Gray Matter of Males With Autism Spectrum Disorder. *Autism Research* **13**, 870–884 (2020).
37. Hattori, T. *et al.* Genetic variants in mannose receptor gene (MRC1) confer susceptibility to increased risk of sarcoidosis. *BMC Medical Genetics* **11**, 151 (2010).
