# Supplementary material for: Phenotypic and genetic associations of quantitative magnetic susceptibility in UK Biobank brain imaging
Source: Nat Neurosci. 2022 May 23;25(6):818–31. doi: 10.1038/s41593-022-01074-w (PMC9174052; doi:10.1038/s41593-022-01074-w)
Supplement: Supplementary file 2 — Reporting Summary [file 41593_2022_1074_MOESM2_ESM.pdf]

nature portfolio

Corresponding author(s): Chaoyue Wang
Karla L. Miller

Last updated by author(s): Apr 3, 2022

Reporting Summary

Nature Portfolio wishes to improve the reproducibility of the work that we publish. This form provides structure for consistency and transparency in reporting. For further information on Nature Portfolio policies, see our Editorial Policies and the Editorial Policy Checklist.

Statistics

For all statistical analyses, confirm that the following items are present in the figure legend, table legend, main text, or Methods section.

n/a	Confirmed	
☐	☒	The exact sample size (*n*) for each experimental group/condition, given as a discrete number and unit of measurement
☐	☒	A statement on whether measurements were taken from distinct samples or whether the same sample was measured repeatedly
☐	☒	The statistical test(s) used AND whether they are one- or two-sided<br>*Only common tests should be described solely by name; describe more complex techniques in the Methods section.*
☐	☒	A description of all covariates tested
☐	☒	A description of any assumptions or corrections, such as tests of normality and adjustment for multiple comparisons
☐	☒	A full description of the statistical parameters including central tendency (e.g. means) or other basic estimates (e.g. regression coefficient) AND variation (e.g. standard deviation) or associated estimates of uncertainty (e.g. confidence intervals)
☐	☒	For null hypothesis testing, the test statistic (e.g. *F*, *t*, *r*) with confidence intervals, effect sizes, degrees of freedom and *P* value noted<br>*Give P values as exact values whenever suitable.*
☒	☐	For Bayesian analysis, information on the choice of priors and Markov chain Monte Carlo settings
☒	☐	For hierarchical and complex designs, identification of the appropriate level for tests and full reporting of outcomes
☐	☒	Estimates of effect sizes (e.g. Cohen's *d*, Pearson's *r*), indicating how they were calculated

Our web collection on statistics for biologists contains articles on many of the points above.

Software and code

Policy information about availability of computer code

Data collection	Data was not explicitly acquired in this study, but originated from the UK Biobank project.<br>Genotype data was collected by Affymetrix using a highly customised version of the Affymetrix software suite Affymetrix Genotyping Console Software (GTC), Affymetrix Power Tools (APT) and SNPolisher R package (Bycroft et al 2018).<br>All MRI data were acquired using 3T Siemens Skyra with software platform VD13 (Alfaro-Almagro et al., 2018 and Miller et al., 2016).
Data analysis	The following software packages were used in this work:<br>• MATLAB R2019a<br>• FMRIB Software Library (FSL) v6.0, https://fsl.fmrib.ox.ac.uk/fsl/fslwiki<br>• FSLNets v0.6, https://fsl.fmrib.ox.ac.uk/fsl/fslwiki/FSLNets<br>• STI Suite v3.0 (including Laplacian phase unwrapping, V-SHARP, iLSQR algorithms), https://people.eecs.berkeley.edu/~chunlei.liu/software.html<br>• MCPC-3D-S v1.6, https://github.com/korbinian90/ASPIRE<br>• MEDI toolbox v2020, http://pre.weill.cornell.edu/mri/pages/qsm.html<br>• Mixture modelling v1.0, https://github.com/allera/One_Dim_Mixture_Models<br>• FUNPACK v1.0, https://git.fmrib.ox.ac.uk/fsl/funpack<br>• Peaks v1.0, novel software for extracting clusters from multi-phenotype GWASs: https://github.com/wnfldchen/peaks<br>• bgenie v1.3, software for efficient GWASs on high-dimensional phenotype data: https://jmarchini.org/bgenie/<br>• LDSC v1.0.1, software for heritability analysis from summary statistics (linkage score regression): https://github.com/bulik/ldsc/<br>The image processing pipelines of the MRI data in the UK Biobank project can be found at https://git.fmrib.ox.ac.uk/falmagro/UK_biobank_pipeline_v_1. Custom-written MATLAB code for quantitative susceptibility mapping (QSM) processing is available at https://git.fmrib.ox.ac.uk/cwang/uk_biobank_qsm_pipeline and will be added to the core UK Biobank brain imaging processing pipeline.

For manuscripts utilizing custom algorithms or software that are central to the research but not yet described in published literature, software must be made available to editors and reviewers. We strongly encourage code deposition in a community repository (e.g. GitHub). See the Nature Portfolio guidelines for submitting code & software for further information.

March 2021

Data

Policy information about availability of data

All manuscripts must include a data availability statement. This statement should provide the following information, where applicable:

- Accession codes, unique identifiers, or web links for publicly available datasets
- A description of any restrictions on data availability
- For clinical datasets or third party data, please ensure that the statement adheres to our policy

The UK Biobank brain imaging data from the early-2020 release of 35,885 participants (and 1,368 participants' repeat imaging data) were used in this study. Permission to use the UK Biobank Resource was obtained via Material Transfer Agreement (https://www.ukbiobank.ac.uk/media/yfob3gln/access_031_f-applicant-mta-data-only-v1-1.pdf) as part of Data Access Application 8107. All imaging data (including raw images, derived maps and IDPs), phenotypes and genetics data are made available by UK Biobank via their standard data access procedure (see http://www.ukbiobank.ac.uk/register-apply). Information on average time from application submission to data release can be found at https://www.ukbiobank.ac.uk/enable-your-research/apply-for-access. The full set of GWAS summary statistics generated in this study can be downloaded from https://www.fmrib.ox.ac.uk/ukbiobank/gwas_resources/index.html. Linkage disequilibrium measurements used in this study were obtained from the 1000 Genomes Project (https://data.broadinstitute.org/alkesgroup/LDSCORE/).

Field-specific reporting

Please select the one below that is the best fit for your research. If you are not sure, read the appropriate sections before making your selection.

- [x] Life sciences
- [ ] Behavioural & social sciences
- [ ] Ecological, evolutionary & environmental sciences

For a reference copy of the document with all sections, see nature.com/documents/nr-reporting-summary-flat.pdf

Life sciences study design

All studies must disclose on these points even when the disclosure is negative.

Sample size

No sample-size calculation was performed, as large epidemiological studies like the UK Biobank project (and others) aim to maximize sample sizes to detect small variations across subjects. We used raw brain swMRI data from the UK Biobank early-2020 release of 35,885 participants. After removal of unusable data for quantitative susceptibility mapping (QSM) processing, we performed QSM processing in 35,273 subjects. In addition, after remove of subjects as part of the genetic processing, we used data from 29,579 subjects for the genome-wide association study where they were randomly split into a discovery sample of 19,720 subjects and a replication sample of 9,859 subjects.
We therefore used the maximum sample size available from this data source. This is by definition "sufficient" given that we are reporting associations that are statistically significant given this maximally available dataset.

Data exclusions

Exclusion criteria were pre-established. With regards to the MRI data, a subject can be excluded based on the T1-weighted scan if registration to standard space fails, likely due to excessive head motion, atypical structure and/or anatomical abnormalities (e.g., large ventricles). Subjects can additionally be excluded from quantitative susceptibility mapping (QSM) analyses if their T2-weighted data are unusable, i.e., no usable ventricle masks (derived from the BIANCA processing of T2-weighted data) to calculate magnetic susceptibility of CSF for QSM referencing. This results in a total of 35,273 subjects with QSM spatial maps and IDPs generated.
Similarly, subjects were selected on usable genetics data. As in Elliott et al., 2018, to avoid confounding effects that may arise from population structure or environmental effects, we selected unrelated subjects with recent British ancestry. Ancestry was determined using sample quality control information provided by UK Biobank. This results in a total of 29,579 subjects for genome-wide association study.

Replication

Full replication analysis carried out once for the genome-wide association study, as described in full detail in Methods. At a significance threshold of -log10(P)>7.5, 292 peak associations were identified with QSM IDPs using the discovery cohort, where 265 replicated at the 5% significance level using the replication cohort; 225 peak associations were identified with T2* IDPs using the discovery cohort, where 199 replicated at the 5% significance level using the replication cohort. Following the approaches from previous UK Biobank brain imaging papers (Miller et al. 2016, Smith et al. 2020), replication analysis was not performed in the phenotypic association study.

Randomization

There is nothing in this study that pertains to randomization. We are using existing data released by UK Biobank. UK Biobank is an observational prospective epidemiological study, and our study use all available subjects that fulfill the criteria described above. Hence there is no equivalent process of randomization that comes into this analysis (this is not a controlled randomised study).

Blinding

There is nothing in this study that pertains to blinding. We are using existing data released by UK Biobank. For exactly the same reasons (this is not a controlled randomised study), there is no step equivalent to blinding involved.

Reporting for specific materials, systems and methods

We require information from authors about some types of materials, experimental systems and methods used in many studies. Here, indicate whether each material, system or method listed is relevant to your study. If you are not sure if a list item applies to your research, read the appropriate section before selecting a response.

Materials & experimental systems

n/a	Involved in the study
☒	☐ Antibodies
☒	☐ Eukaryotic cell lines
☒	☐ Palaeontology and archaeology
☒	☐ Animals and other organisms
☐	☒ Human research participants
☒	☐ Clinical data
☒	☐ Dual use research of concern

Methods

n/a	Involved in the study
☒	☐ ChIP-seq
☒	☐ Flow cytometry
☐	☒ MRI-based neuroimaging

Human research participants

Policy information about studies involving human research participants

Population characteristics

We used data from 35,885 participants in the UK Biobank early-2020 release who had susceptibility-weighted MRI data collected. Participants were 53.11% female and aged 45-82yo (64.04±7.5yo) at time of imaging. Of these participants, 1,447 were recruited for a repeat scan approximately 2 years (2.25±0.12y) after the first imaging session.

Recruitment

We used existing Open data from UK Biobank and were not involved in recruitment. Recruitment was through the UK National Health Service, as described in:
Sudlow, C. et al. UK Biobank: an open access resource for identifying the causes of a wide range of complex diseases of middle and old age. PLoS Med. 12, e1001779 (2015).

Ethics oversight

UK Biobank has approval from the North West Multi-centre Research Ethics Committee (MREC) to obtain and disseminate data and samples from the participants (https://www.ukbiobank.ac.uk/ethics/), and these ethical regulations cover the work in this study. Written informed consent was obtained from all participants.

Note that full information on the approval of the study protocol must also be provided in the manuscript.

Magnetic resonance imaging

Experimental design

Design type

Please see "Methods" for full details. Our analyses include data from structural MRI (T1 and T2 FLAIR) and susceptibility-weighted MRI.

Design specifications

Not applicable, as our analyses did not use any functional MRI data.

Behavioral performance measures

Behavioral performance in the MRI scanner was not used in this study.

Acquisition

Imaging type(s)

T1-weighted MRI, T2-weighted MRI and susceptibility-weighted MRI.

Field strength

3T

Sequence & imaging parameters

Susceptibility-weighted MRI: 3D dual-echo gradient echo (GRE) sequence
TE1=9.4ms, TE2=20ms, TE=27ms and an in-plane acceleration factor of 2
Field of view: 256x288x48 matrix
Resolution: 0.8x0.8x3.0 mm

T1-weighted MRI: 3D MPRAGE protocol
TI = 880 ms, TR = 2000 ms and an in-plane acceleration factor of 2
Field of view 208x256x256 matrix
Resolution 1.0x1.0x1.0 mm

T2-weighted MRI: Fluid-attenuated inversion recovery (FLAIR) protocol (3D SPACE)
TI = 1800ms, TR = 5000 ms and an in-plane acceleration factor of 2
Field of view: 192x256x56 matrix
Resolution: 1.05x1.0x1.0 mm

Area of acquisition

Whole brain

Diffusion MRI ☐ Used ☒ Not used

Preprocessing

Preprocessing software

Preprocessing of T1- and T2- weighted MRI data were described in Alfaro-Almagro et al., 2018 and Miller et al., 2016.

Preprocessing software	The QSM processing pipeline of susceptibility-weighted MRI data was developed as part of this work, using the STI Suite toolbox (https://people.eecs.berkeley.edu/~chunlei.liu/software.html) and custom scripts (https://git.fmrib.ox.ac.uk/cwang/uk_biobank_qsm_pipeline) as described in the paper.
Normalization	Quantitative susceptibility mapping (QSM) spatial maps were transformed using FNIRT (part of FSL) to a 1-mm T1 template in MNI-space, transformations were provided by the UK Biobank brain processing pipeline (as described in Alfaro-Almagro et al., 2018).
Normalization template	MNI152_T1_1mm included in the FSL v6.0 software package.
Noise and artifact removal	No noise or artifact removal was performed to susceptibility-weighted MRI data.
Volume censoring	No volume censoring was performed to susceptibility-weighted MRI data.

Statistical modeling & inference

Model type and settings	Standard association studies with multiple comparison correction (Bonferroni, FDR) in deep gray matter and white matter hyperintensity ROIs.
Effect(s) tested	Not applicable

Specify type of analysis: ☐ Whole brain ☒ ROI-based ☐ Both

Anatomical location(s)	Deep gray structures (accumbens, amygdala, caudate, hippocampus, pallidum, putamen and thalamus) segmented using FIRST (part of FSL), substantia nigra segmented using an atlas in MNI space (https://doi.org/10.17605/OSF.IO/JKZWP), and white matter hyperintensities segmented using BIANCA (part of FSL).
Statistic type for inference (See Eklund et al. 2016)	Not applicable
Correction	Bonferroni correction for family-wise error (FWE) control at Pcorrected<0.05; and 5% false discovery rate (FDR).

Models & analysis

n/a	Involved in the study
☒	☐ Functional and/or effective connectivity
☒	☐ Graph analysis
☒	☐ Multivariate modeling or predictive analysis
